# Supplementary material for: Two duplicated GhMML3 genes coordinately control development of lint and fuzz fibers in cotton
Source: Plant Commun. 2025 Feb 12;6(4):101281. doi: 10.1016/j.xplc.2025.101281 (PMC12010375; doi:10.1016/j.xplc.2025.101281)
Supplement: Document S1. Figures S1–S21 [file mmc1.pdf]

Plant Communications, Volume *6*

Supplemental information

Two duplicated *GhMML3* genes coordinately control development of lint and fuzz fibers in cotton

Rui Chen, Jun Zhang, Jun Li, Jinwen Chen, Fan Dai, Yue Tian, Yan Hu, Qian-Hao Zhu, and Tianzhen Zhang

Supplemental information for

Duplicated *GhMML3s* coordinately control cotton lint and fuzz fiber development

Rui Chen[1,2#], Jun Zhang[1,3#], Jun Li[2], Jinwen Chen[1], Fan Dai[1], Yue Tian[4], Yan Hu[1,2], Qian-Hao Zhu[5], Tianzhen Zhang[1,2*]

1. Zhejiang Provincial Key Laboratory of Crop Genetic Resources, Institute of Crop Science, Plant Precision Breeding Academy, College of Agriculture and Biotechnology, Zhejiang University, Hangzhou, China
2. Hainan Institute of Zhejiang University, Sanya, Hainan, 572025, China
3. Institute of Horticulture, Zhejiang Academy of Agricultural Sciences, Hangzhou, Zhejiang, China.
4. College of Biotechnology, Jiangsu University of Science and Technology, Zhenjiang, China.
5. CSIRO Agriculture and Food, GPO Box 1700, Canberra 2601, Australia

[#] These authors contributed equally to this work.

[*]Correspondence and requests for materials should be addressed to Dr. Tianzhen Zhang (cotton@zju.edu.cn)

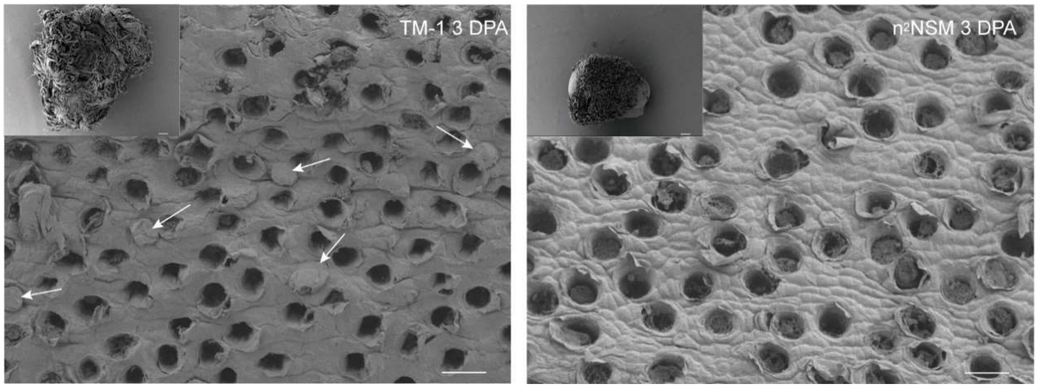

Supplemental Figure 1. Scanning electronic micrographs of TM-1 and n_2NSM at 3 DPA.

Arrows indicate fuzz cells. Scale bar = 20 μm.

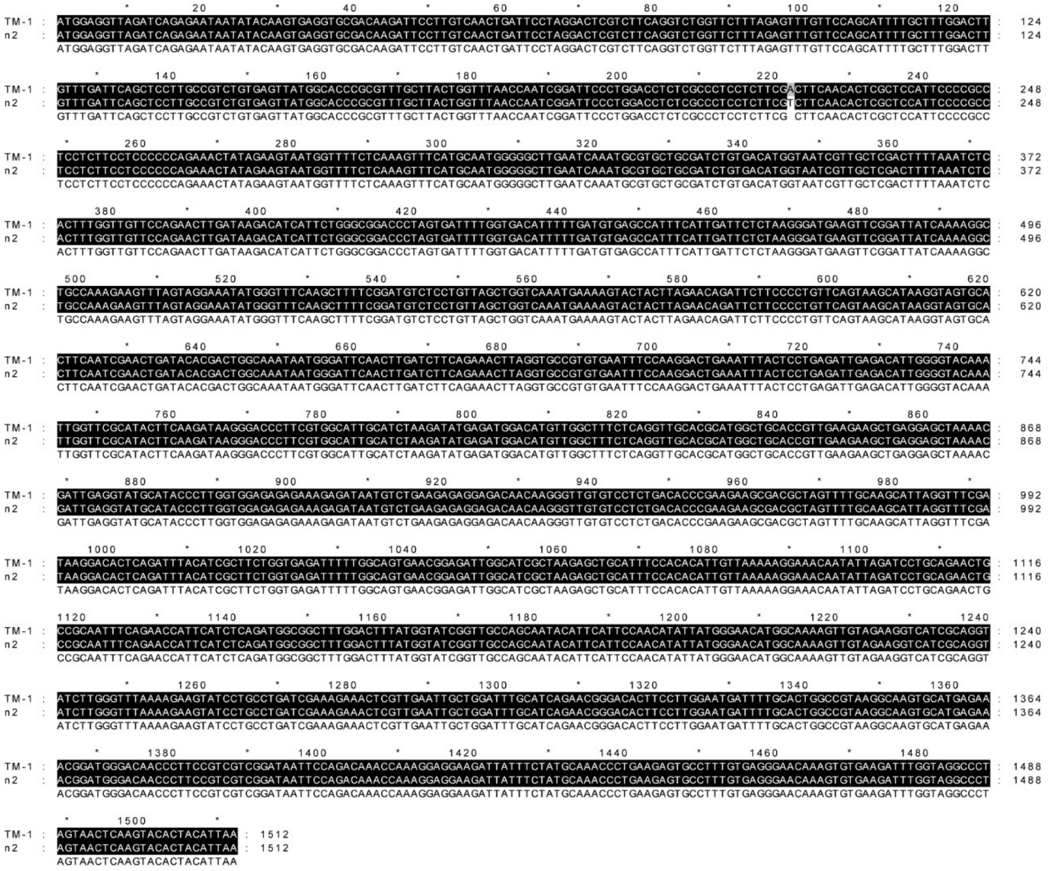

Supplemental Figure 2. Alignment of *ORF1* coding sequences from the accessions used in this study.

Black shading indicates coding sequences from n_2NSM and TM-1. On the gray backdrop, there is a single-base difference at 222 bp, but this difference has no effect on amino acid sequence.

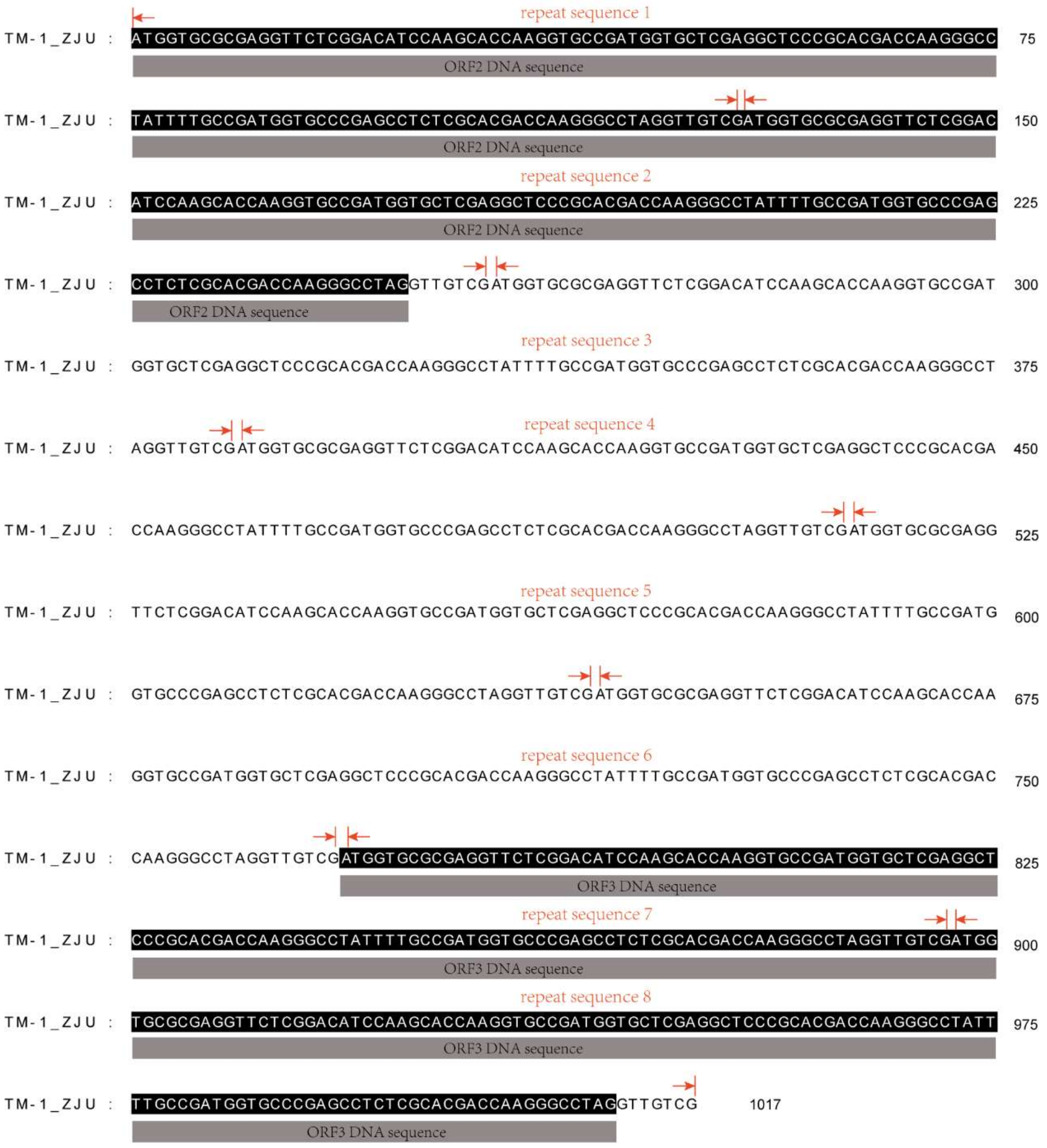

Supplemental Figure 3. The genome sequences of *ORF2* and *ORF3* from the accessions used in this study.

Black shading indicates DNA sequences from *Gossypium hirsutum* (ZJU assembly). The eight red rounded arrows in this region indicate the same repeated sequence (ATGGTG….TTGTCG).

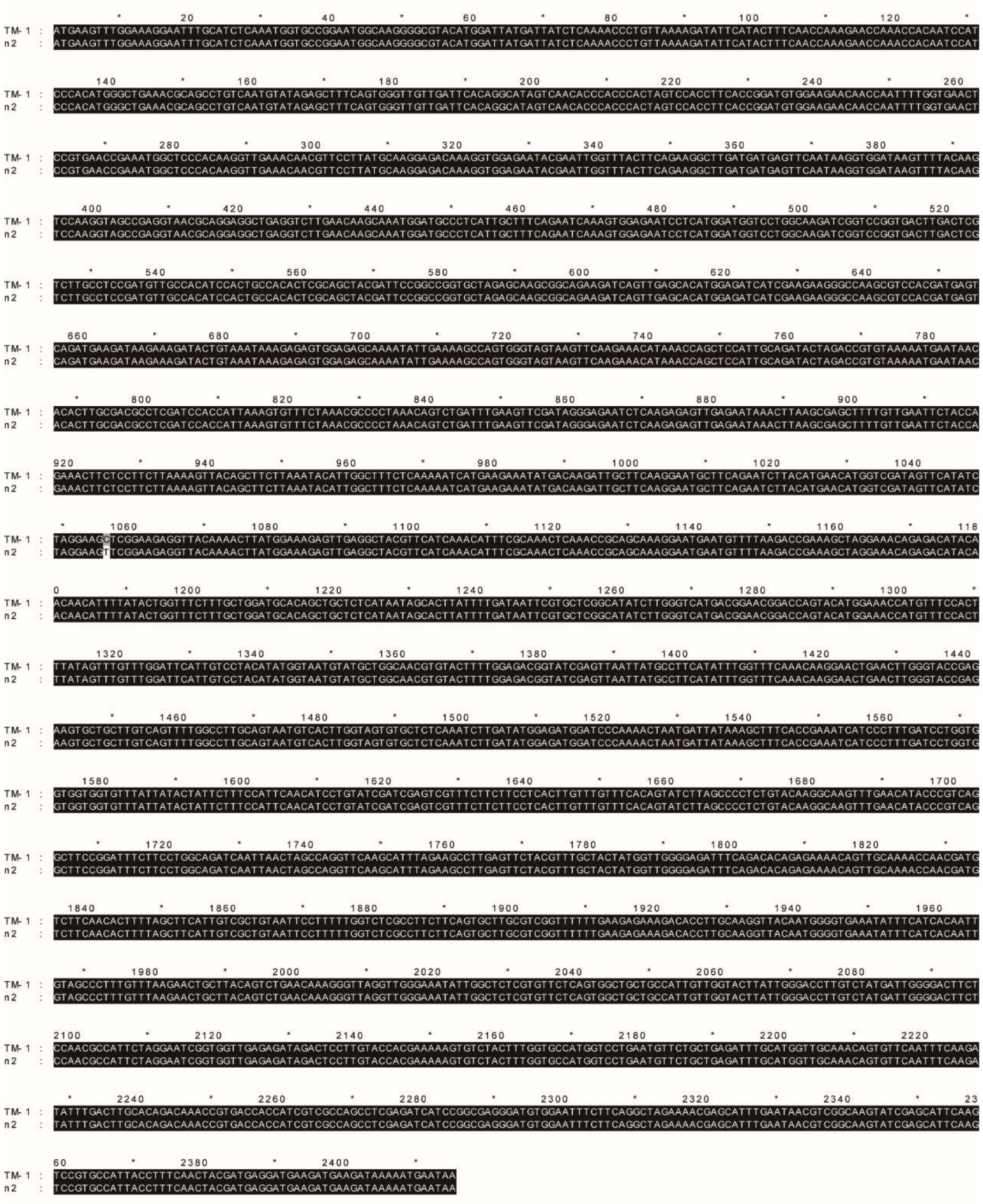

Supplemental Figure 4. Alignment of *ORF4* coding sequences from the accessions used in this study.

Black shading indicates coding sequences from TM-1 and n_2NSM. On the gray backdrop, there is a single-base difference at 1056 bp, but this difference has no effect on amino acid sequence.

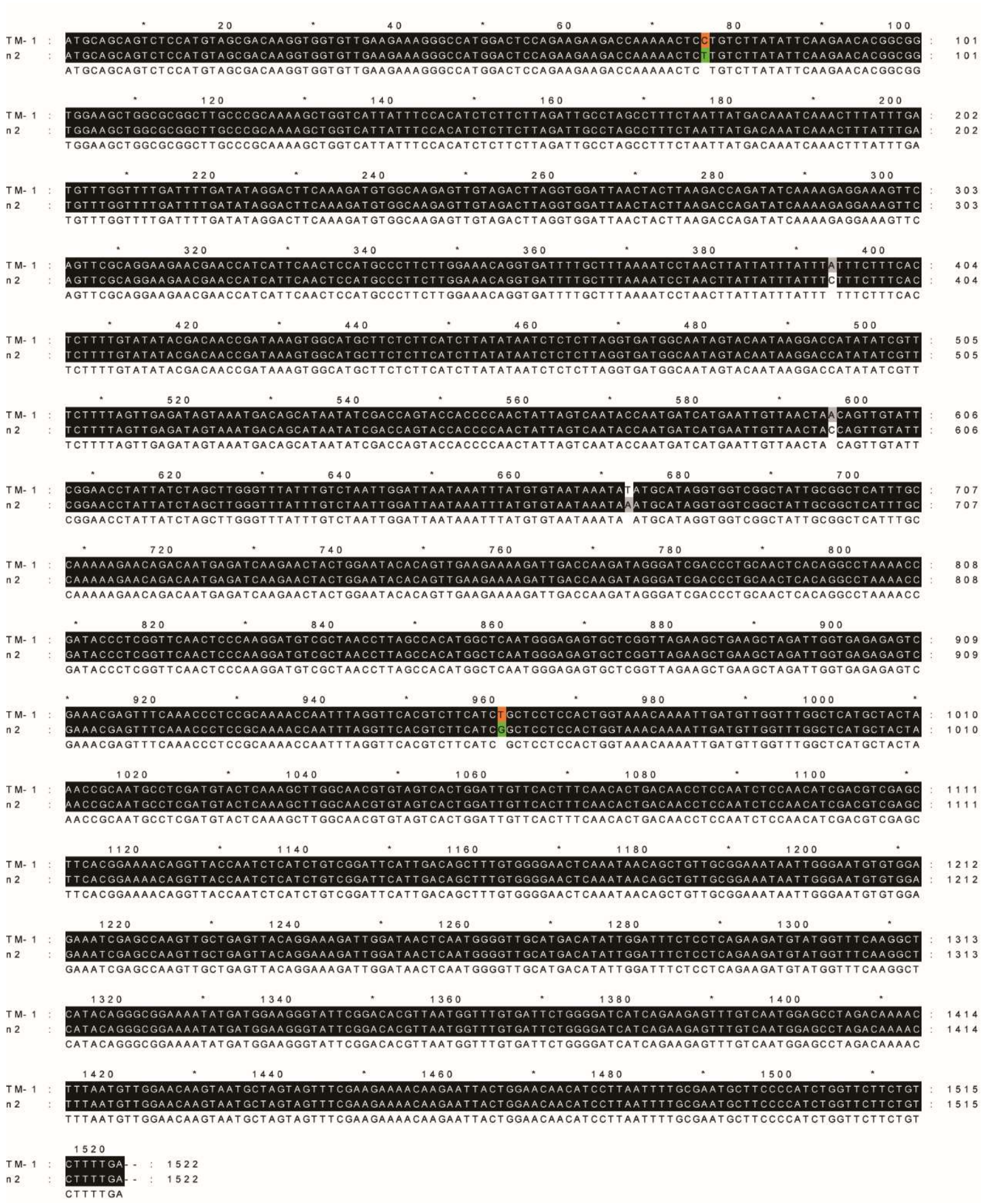

Supplemental Figure 5. Alignment of *GhMML3_D12* genome sequences from the accessions used in this study.

Black shading indicates coding sequences from TM-1 and n_2NSM. Orange and green backgrounds indicate SNP differences. There are two SNPs in the exons, at 76 bp and 961 bp respectively, but neither causes an amino acid difference.

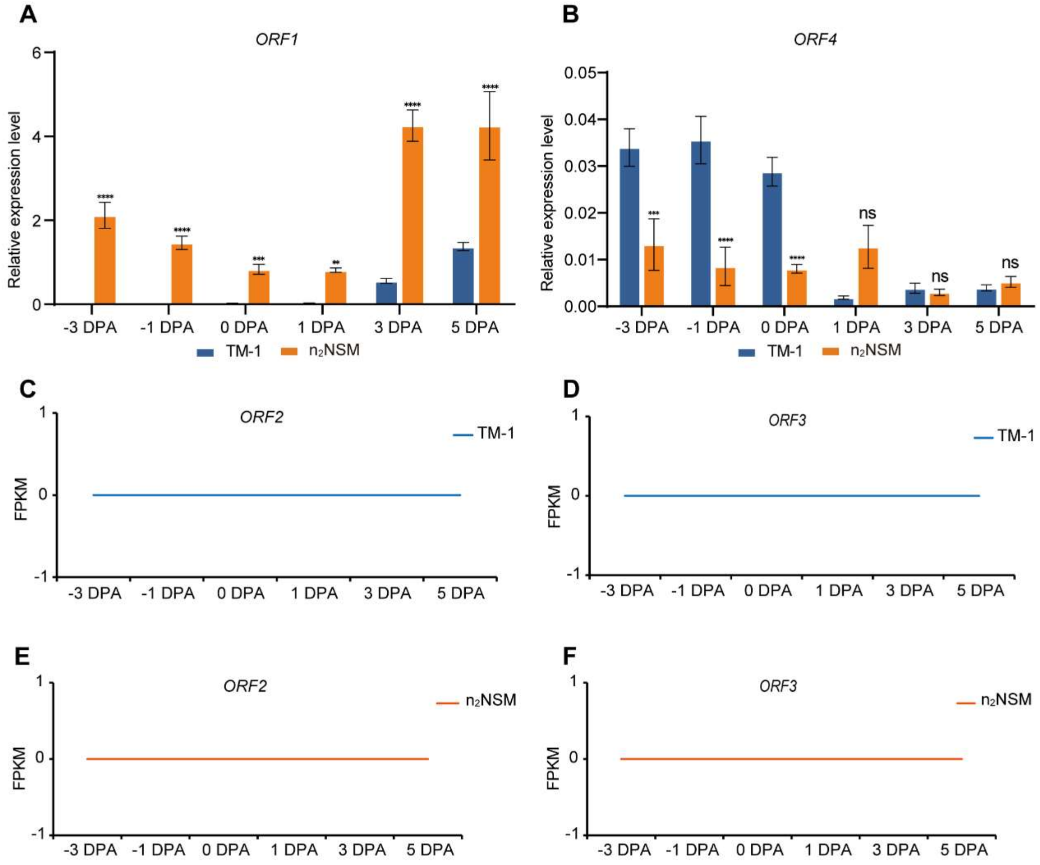

Supplemental Figure 6. Expression of *ORF1-4* in TM-1 and n_2NSM.
(A–B) Quantitative reverse transcription PCR of *ORF1* and *ORF4* in ovules of TM-1 and n_2NSM. Statistical significance was determined using one-way ANOVA. Data are represented as means ± SEM of three biological replicates. ****, $P < 0.0001$; ***, $P < 0.001$; **, $P < 0.01$. **(C–D)** Expression of *ORF2* and *ORF3* (as FPKM) in RNA-seq data from different developmental stages (including ovules at -3, -1, 0, 1, 3, 5 DPA) of TM-1 and n_2NSM.

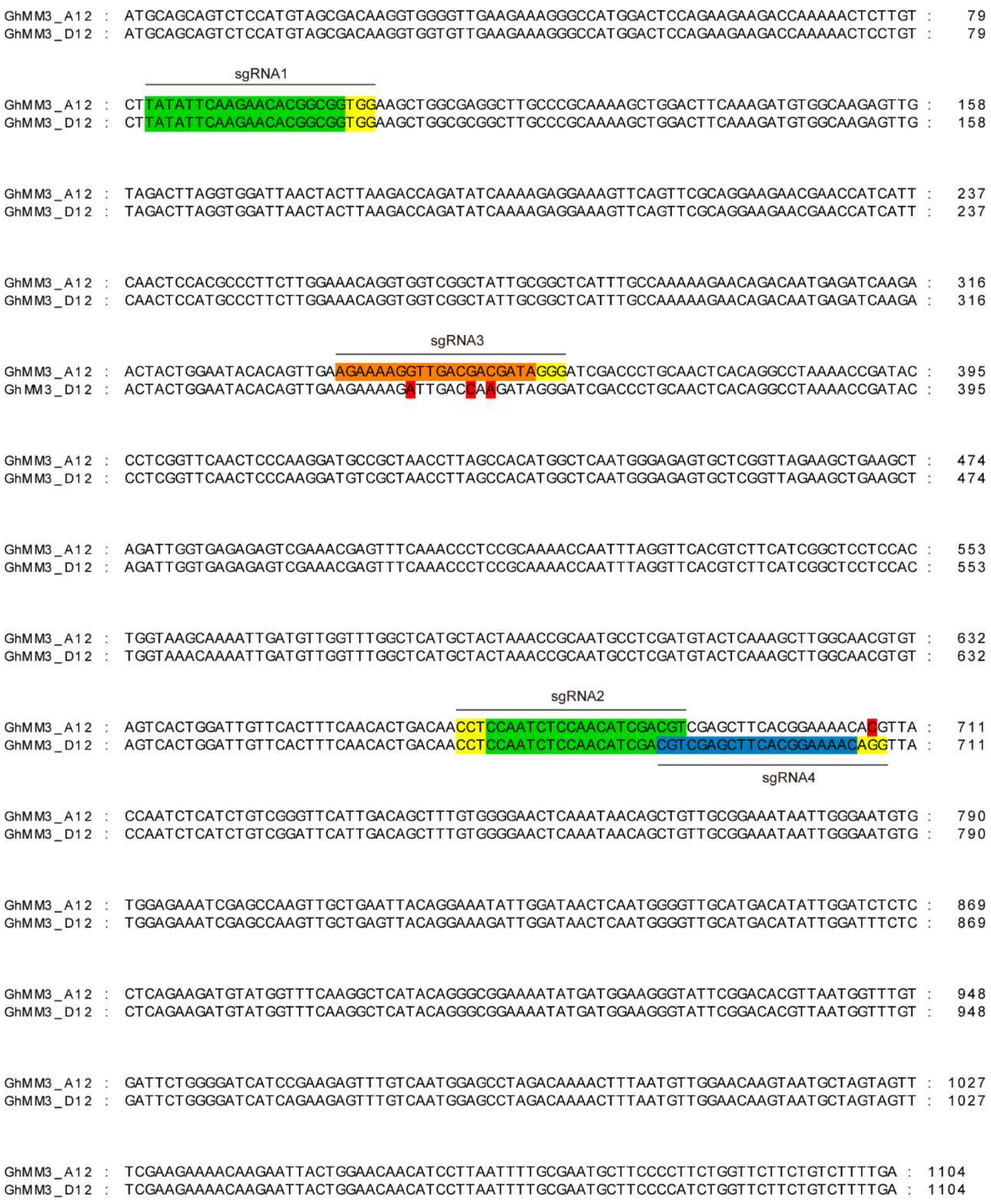

Supplemental Figure 7. Location of sgRNAs in *GhMML3* genes.

sgRNA1 and sgRNA2 were used for simultaneous editing of *GhMML3_A12* and *GhMML3_D12* (*#mml3s*), while sgRNA3 targeted *GhMML3_A12* alone (*#mml3-A12*) and sgRNA4 *GhMML3_D12* alone (*#mml3-D12*).

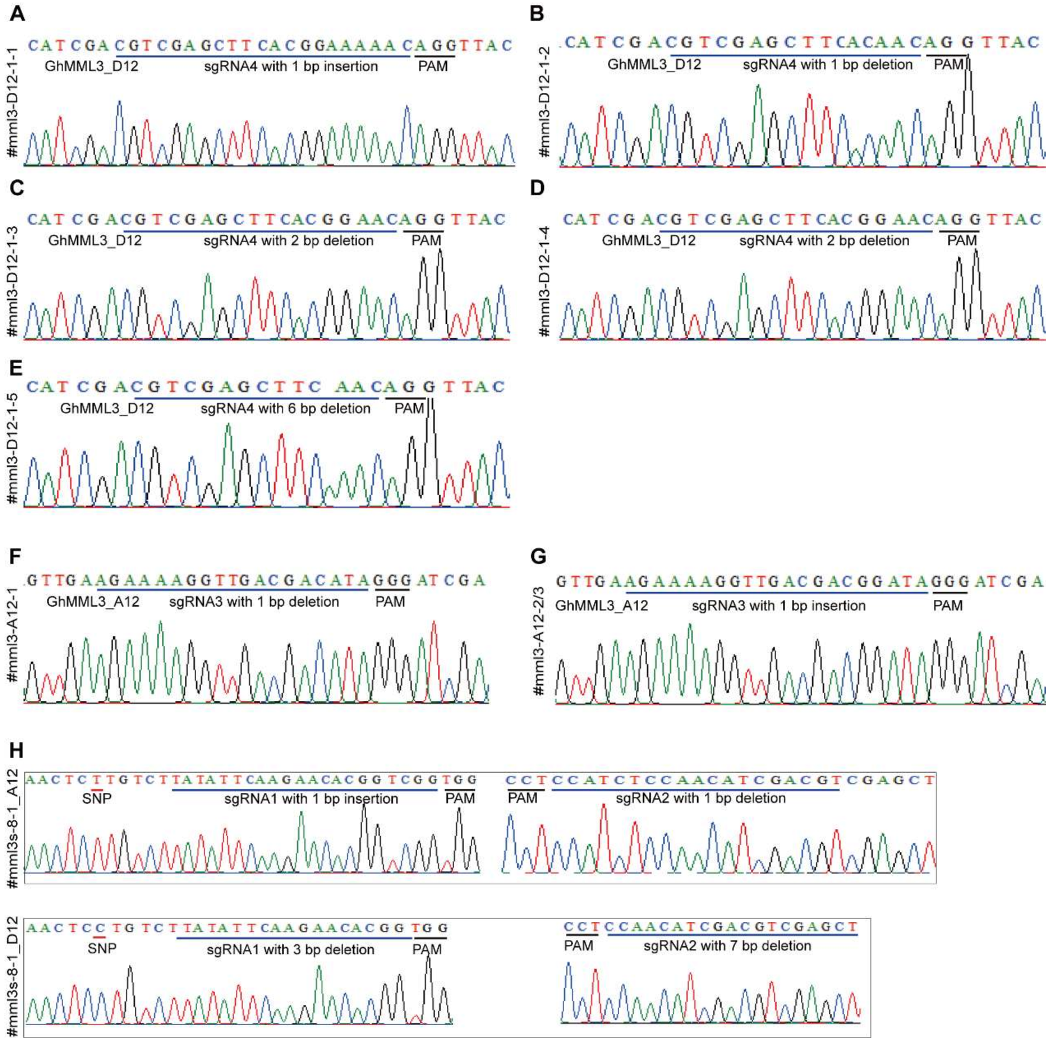

Supplemental Figure 8. Sequencing peak traces at sgRNA target sites in transgenic plants.

(A–E) Five sequencing traces showing *GhMML3_D12* gene editing status in *#mml3-D12* plants. **(F–G)** Two sequencing traces showing *GhMML3_A12* status in *#mml3-A12* plants. **(H)** Sequencing traces showing *GhMML3_A12* and *GhMML3_D12* status in *#mml3s-8-1* plants. PAM regions and sgRNAs sites are underlined with black and blue lines, respectively.

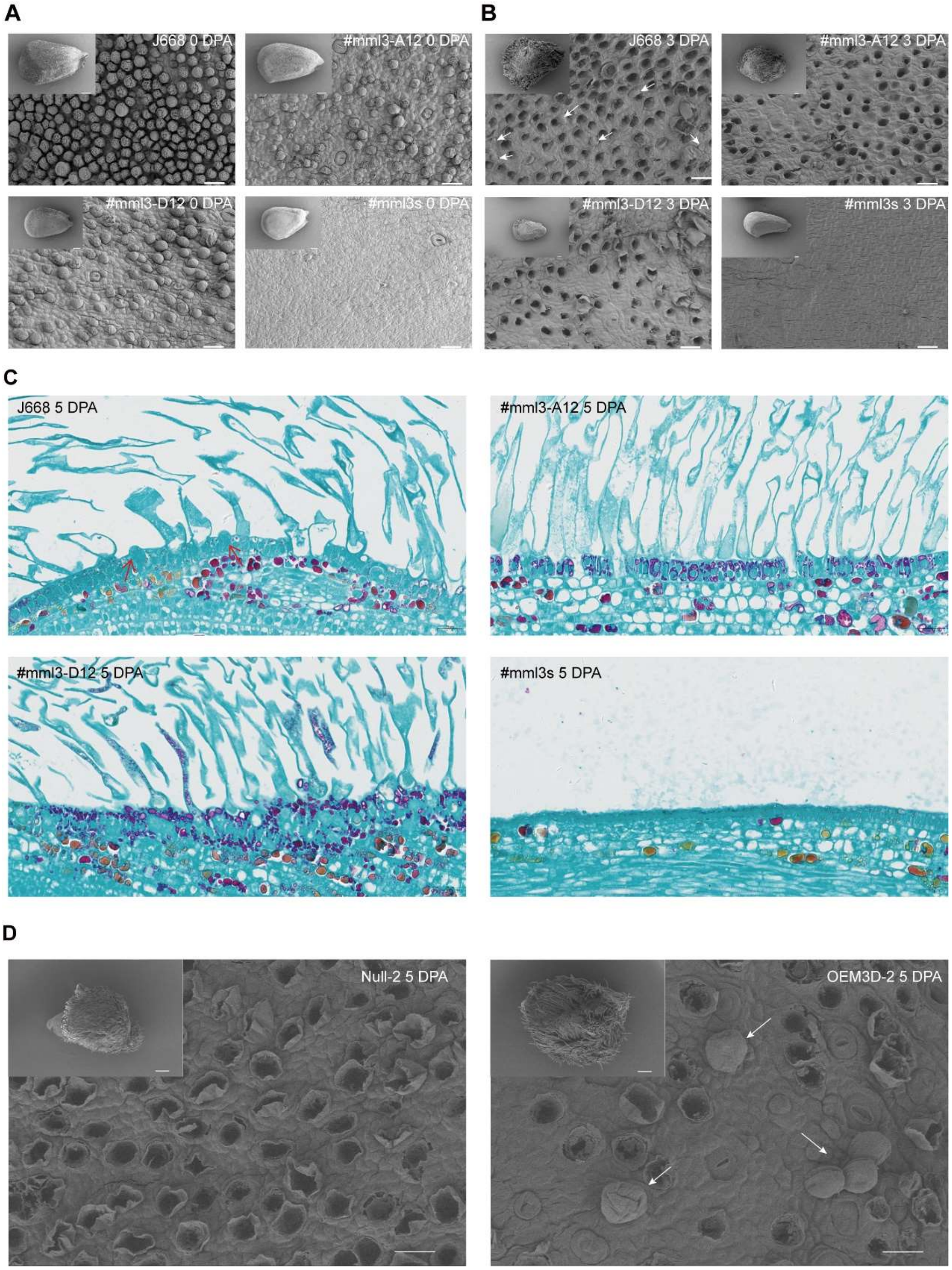

Supplemental Figure 9. Scanning electron micrographs and paraffin sections of *GhMML3* transgenic plants.

(A–B) Scanning electronic micrographs of *#mml3-A12*, *#mml3-D12*, and *#mml3-8-1* of ovules at 0 and 3 DPA. Arrows indicate initiating fuzz fiber cells. Scale bar = 20 μm (bottom), 200 μm (top). **(C)** paraffin sections of ovules of transgenic plants at 5 DPA. Red arrows indicate fuzz cells. Scale bar = 25 μm. **(D)** Scanning electronic micrographs of Null-2 and *OEM3D-2* seed epidermises at 5 DPA. Arrows indicate initiating fuzz fiber cells. Scale bar = 20 μm (bottom), 200 μm (top).

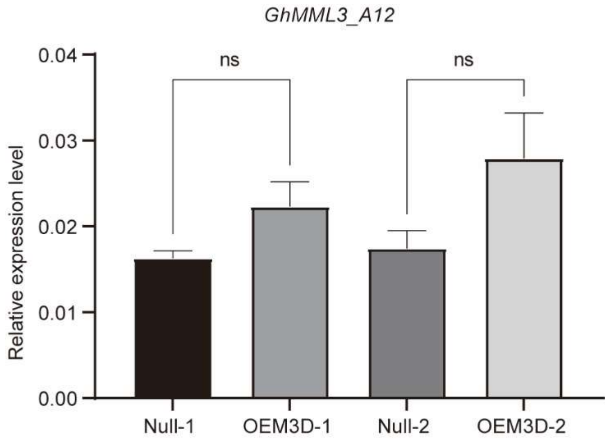

Supplemental Figure 10. Quantitative reverse transcription PCR of *GhMML3_A12* in plants over-expressing *GhMML3_D12*.

Statistical significance was determined using one-way ANOVA. Data are represented as means ± SEM of three biological replicates.

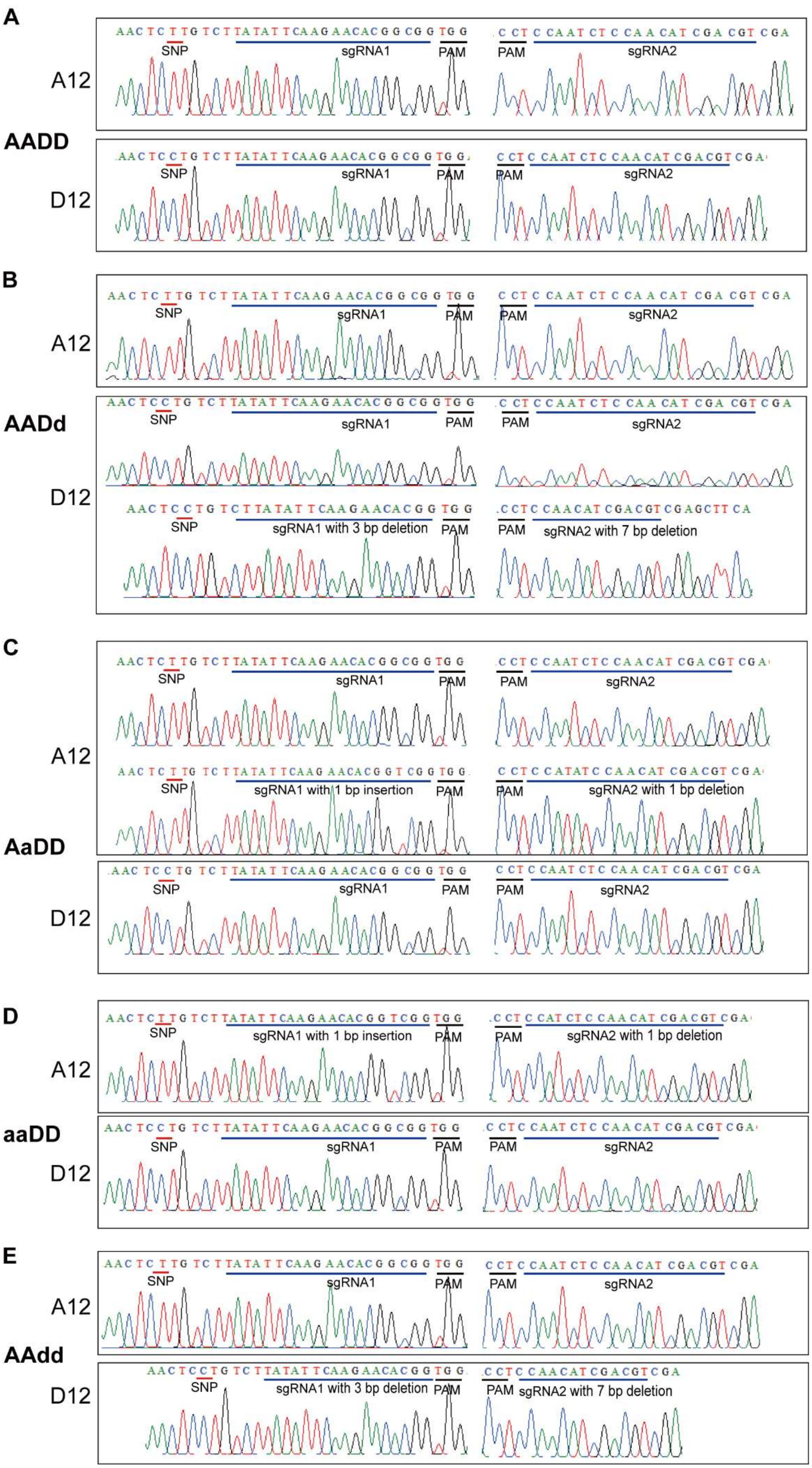
A
A12
AADD
D12
SNP
sgRNA1
PAM
sgRNA2
B
A12
AADd
D12
sgRNA1 with 3 bp deletion
sgRNA2 with 7 bp deletion
C
A12
sgRNA1 with 1 bp insertion
sgRNA2 with 1 bp deletion
AaDD
D12
D
A12
sgRNA1 with 1 bp insertion
sgRNA2 with 1 bp deletion
aaDD
D12
E
A12
AAdd
D12
sgRNA1 with 3 bp deletion
sgRNA2 with 7 bp deletion

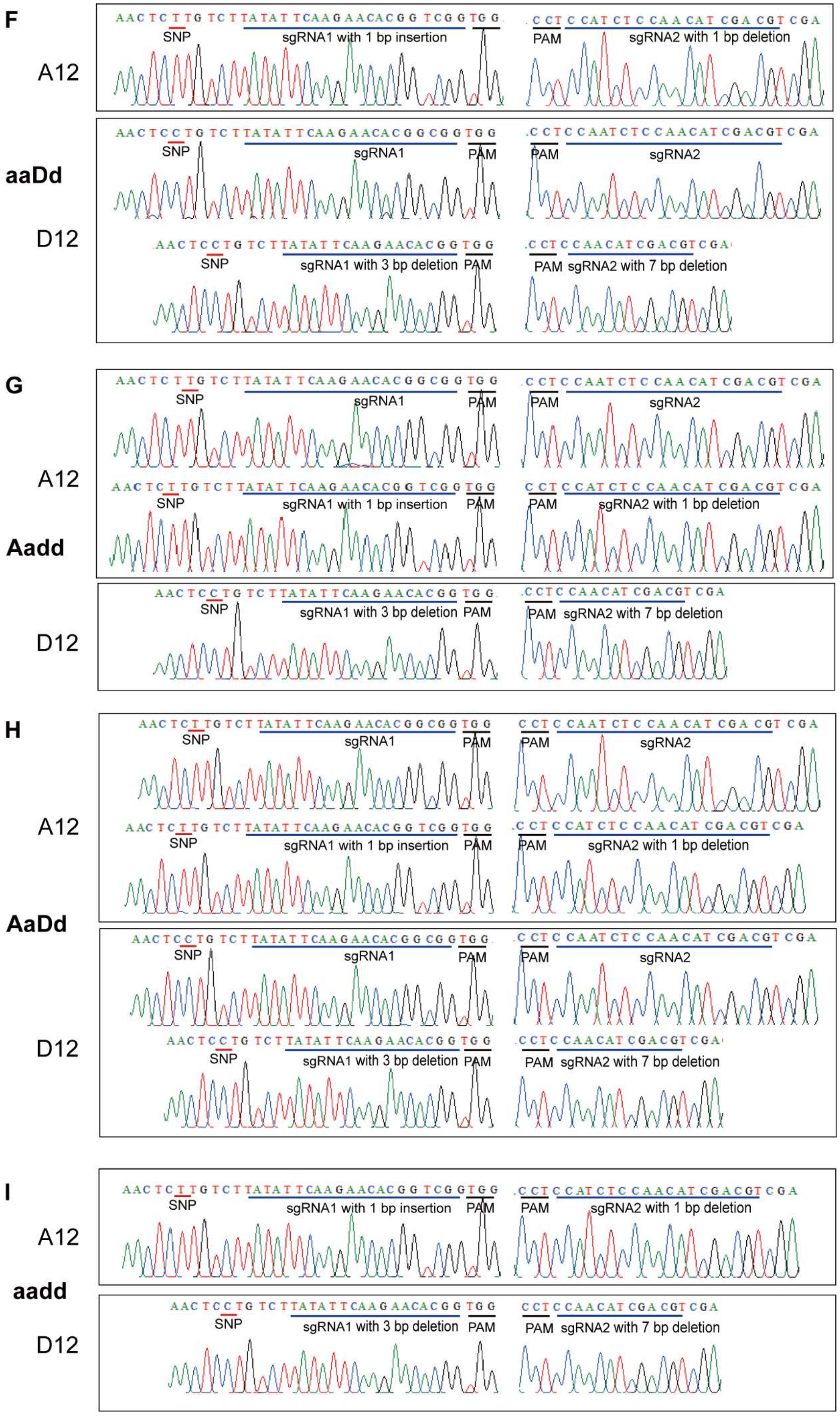

F
A12
SNP
sgRNA1 with 1 bp insertion
PAM
sgRNA2 with 1 bp deletion
aaDd
SNP
sgRNA1
PAM
PAM
sgRNA2
D12
SNP
sgRNA1 with 3 bp deletion
PAM
PAM
sgRNA2 with 7 bp deletion
G
SNP
sgRNA1
PAM
PAM
sgRNA2
A12
SNP
sgRNA1 with 1 bp insertion
PAM
PAM
sgRNA2 with 1 bp deletion
Aadd
SNP
sgRNA1 with 3 bp deletion
PAM
PAM
sgRNA2 with 7 bp deletion
D12
H
SNP
sgRNA1
PAM
PAM
sgRNA2
A12
SNP
sgRNA1 with 1 bp insertion
PAM
PAM
sgRNA2 with 1 bp deletion
AaDd
SNP
sgRNA1
PAM
PAM
sgRNA2
D12
SNP
sgRNA1 with 3 bp deletion
PAM
PAM
sgRNA2 with 7 bp deletion
I
SNP
sgRNA1 with 1 bp insertion
PAM
PAM
sgRNA2 with 1 bp deletion
A12
aadd
SNP
sgRNA1 with 3 bp deletion
PAM
PAM
sgRNA2 with 7 bp deletion
D12

Supplemental Figure 11. Sequencing peak traces of sgRNA target sites in (#*mml3s-8-1* × J668)F_2.

(A–I) Wild-type *GhMML3_A12* and *GhMML3_D12* are denoted as “A” and “D”, and the corresponding edited versions as “a” and “d”. Red lines indicate the SNPs used to distinguish chromosomes A12 and D12. PAM regions and sgRNAs sites are underlined with black and blue lines, respectively.

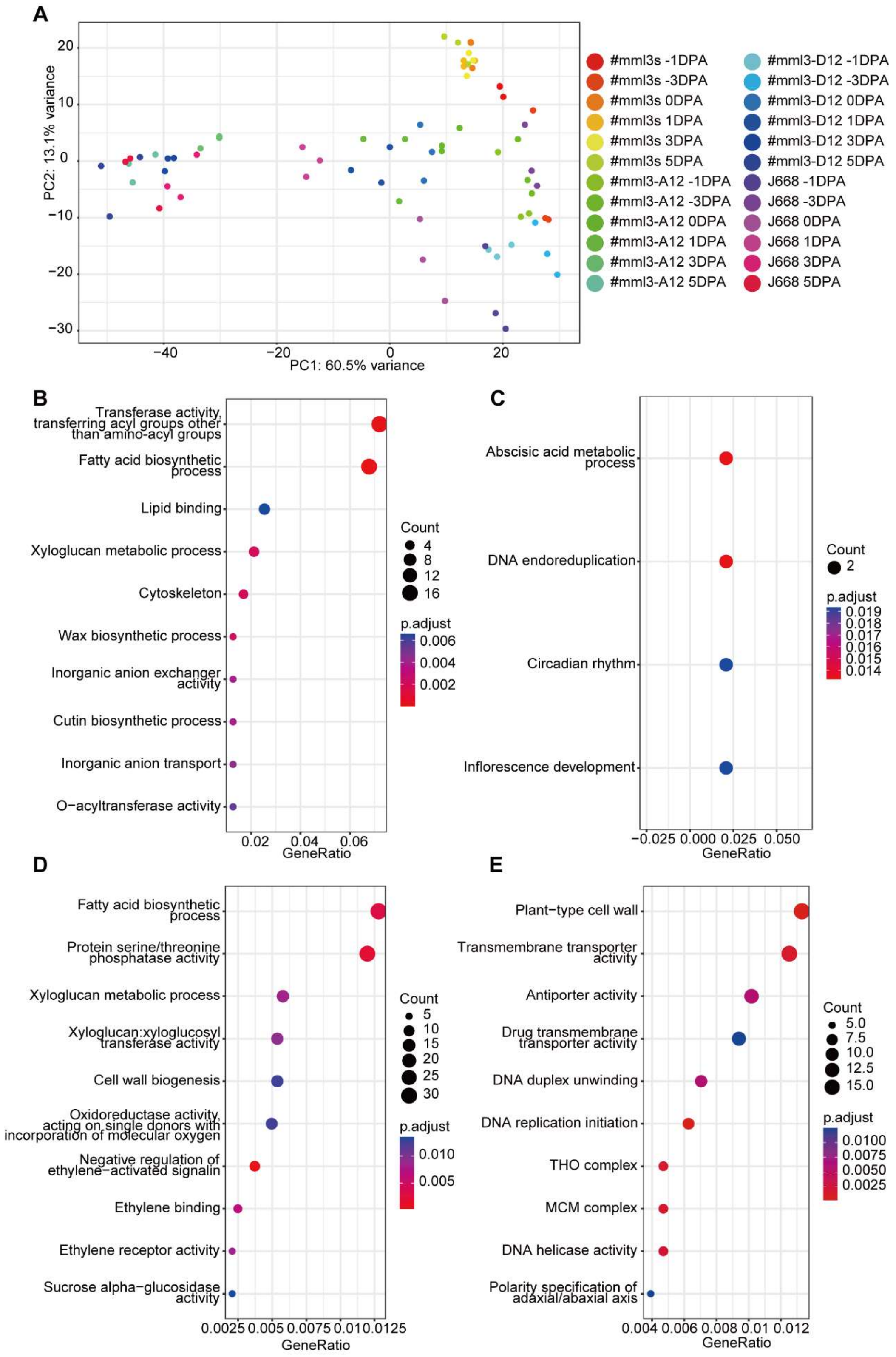

Supplemental Figure 12. RNA-seq analysis of *GhMML3* gene-edited materials. **(A)** PCA analysis of RNA data from J668, *#mml3s*, *#mml3-A12*, and *#mml3-D12* plants. **(B–C)** Gene Ontology (GO) enrichment analysis of down- and up-regulated

DEGs in fuzzless-linted mutants (*#mml3-A12*/*#mml3-D12*) vs J668. **(D–E)** GO enrichment analysis of down- and up-regulated DEGs in *#mml3s* vs J668.

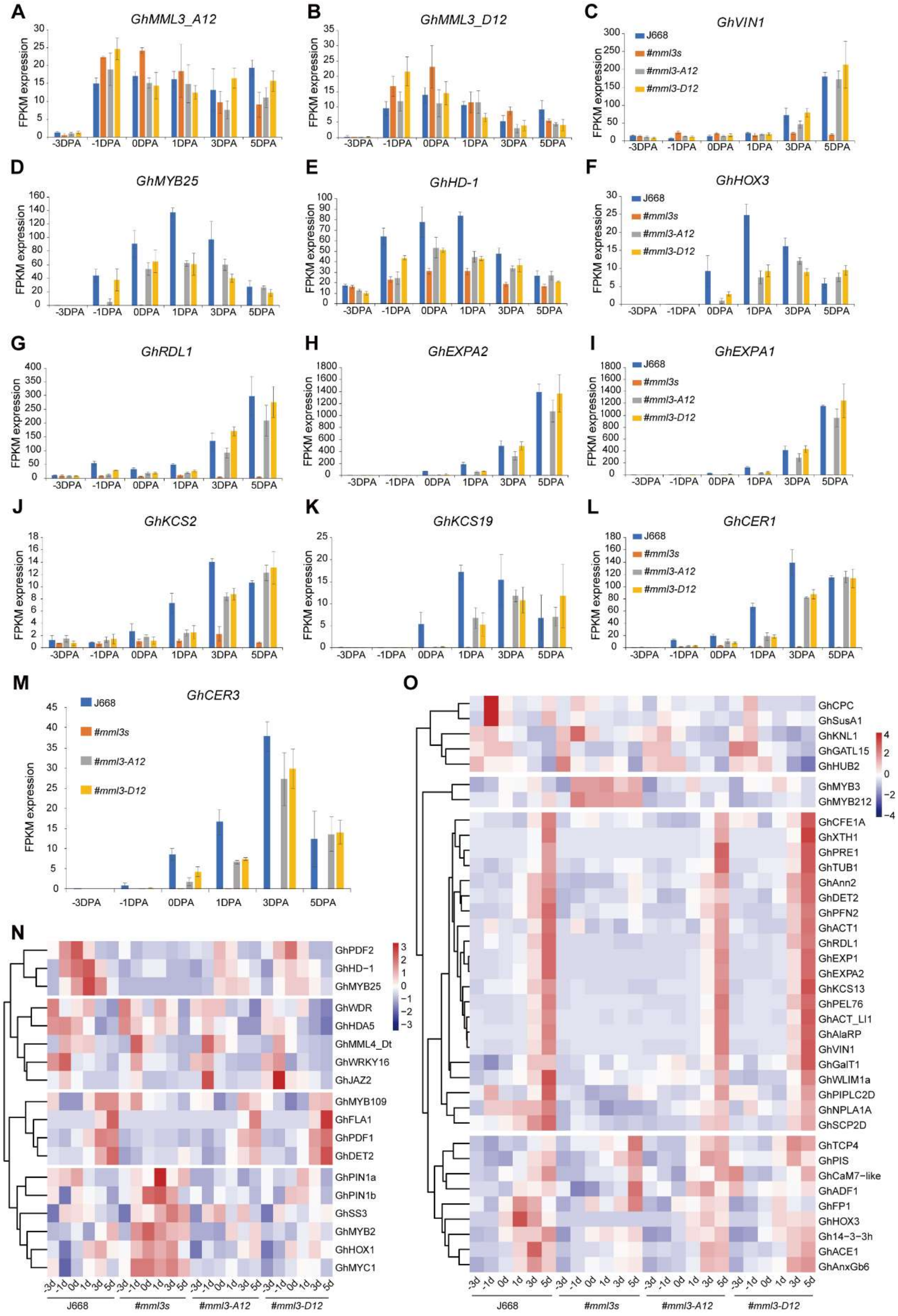

Supplemental Figure 13. Expression profiles of fiber development-related genes in *GhMML3* gene-edited materials.

(A–M) Expression profiles of important fiber development-related genes in J668, *#mml3s*, *#mml3-A12*, and *#mml3-D12*. Expression is presented as FPKM values.

Error bars represent SD of three biological replicates. **(N)** RNA-seq expression heatmap of known fiber initiation-related genes in J668, *#mml3s*, *#mml3-A12*, and *#mml3-D12*. **(O)** RNA-seq expression heatmap of known fiber elongation-related genes in J668, *#mml3s*, *#mml3-A12*, and *#mml3-D12*.

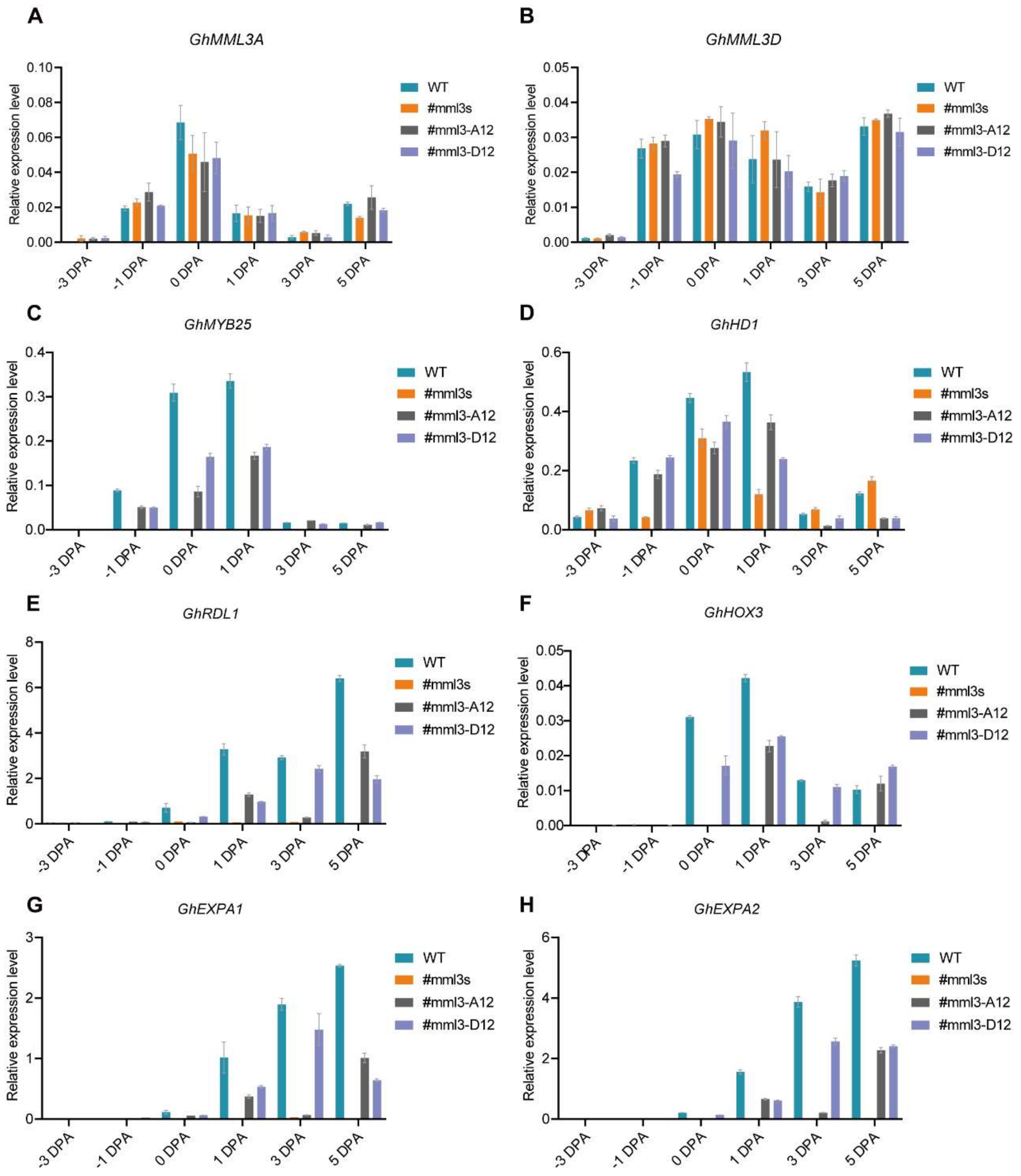

Supplemental Figure 14. Quantitative reverse transcription PCR determination of fiber development-related genes expression in the ovules of gene editing lines. The data are presented as the mean ± SEM of three biological replicates.

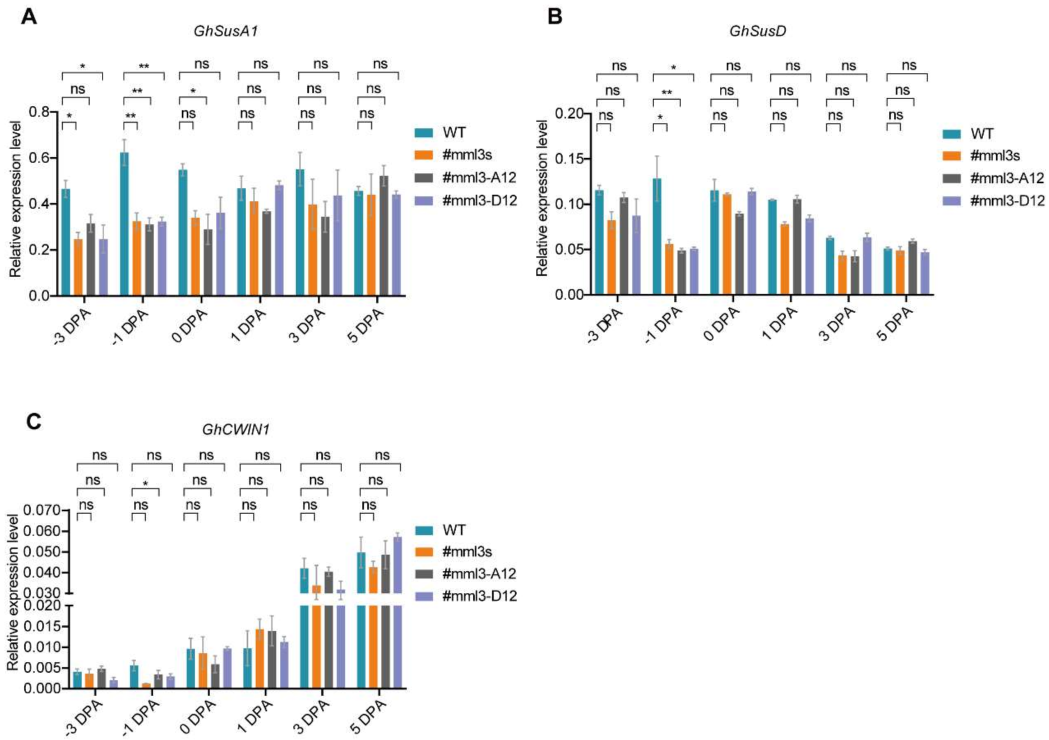

Supplemental Figure 15. Quantitative reverse transcription PCR determination of *GhSusA1*, *GhSusD* and *GhCWIN1* expression in the ovules of gene editing lines.
(A-C) Statistical significance was determined using one-way ANOVA. Data are represented as means ± SEM of three biological replicates.

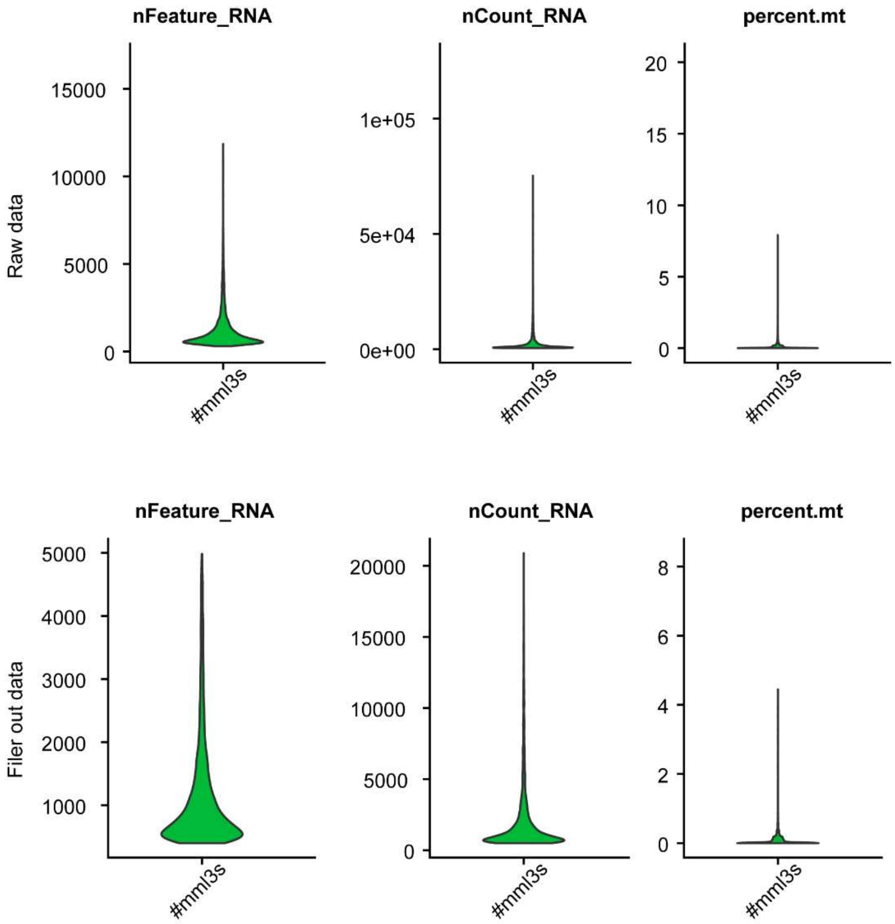

Supplemental Figure 16. Data processing for single-cell.
Raw sequencing data and filter out data of scRNA data of #*mml3s*.

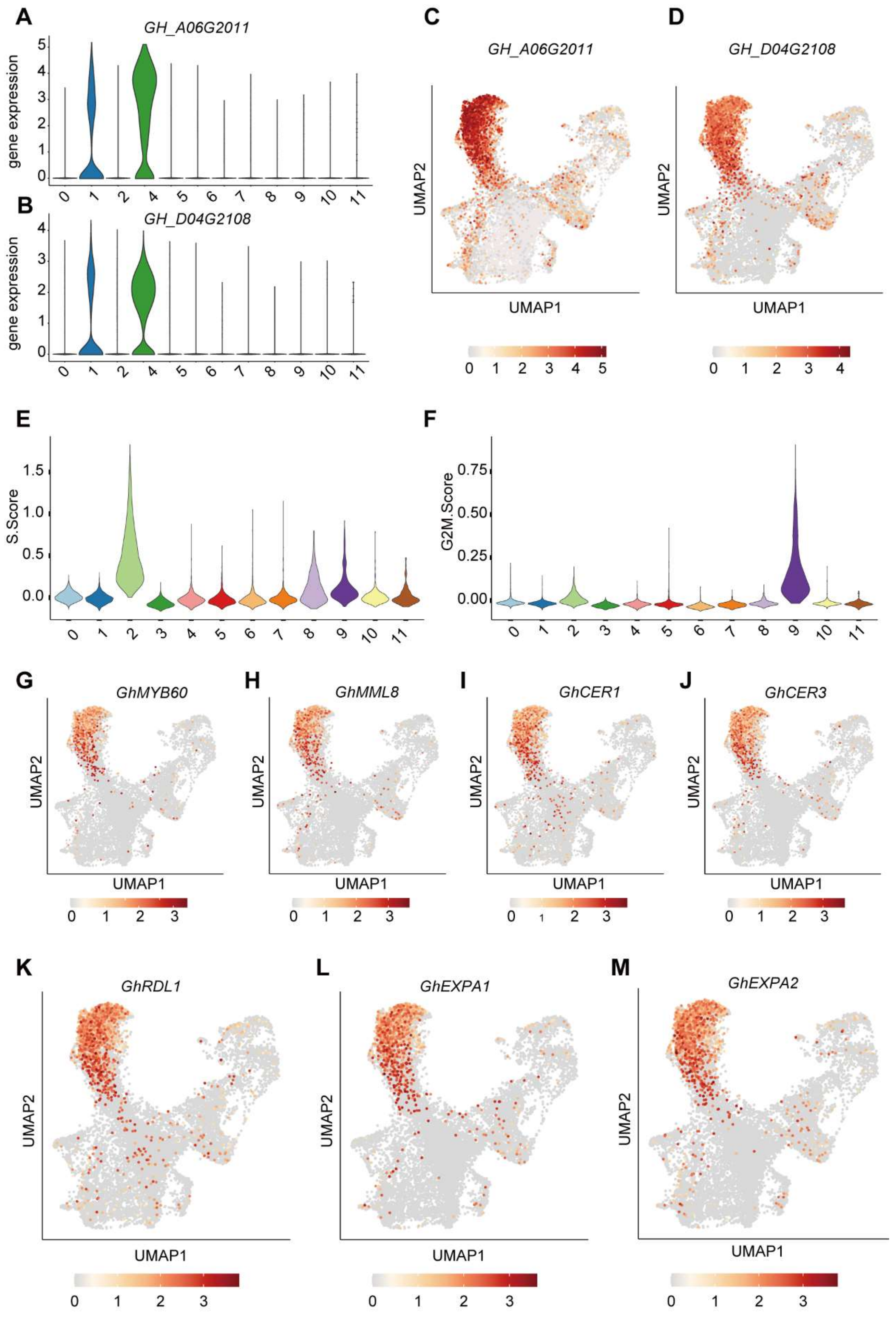

Supplemental Figure 17. Annotation of fiber cells and proliferating cell types. **(A–B)** Violin plots depicting the expression of *GhMYB25* (*GH_D04G2108*) and *GhPPO7* (*GH_A06G2011*). **(C–D)** UMAP plots representing the expression patterns of *GhMYB25* (*GH_D04G2108*) and *GhPPO7* (*GH_A06G2011*). **(E–F)** Plot of cell

cycle fractions suggesting that Cluster 2 and Cluster 9 are respectively associated with the S and G2M phases. **(G–J)** Expression profiles of *GhMML3_D12* in different clusters of #*mml3s* and WT. **(K–N)** Respective UMAP plots representing the expression patterns of *GhMYB25*, *GhHD-1*, *GhVIN1*, and *GhHOX3*.

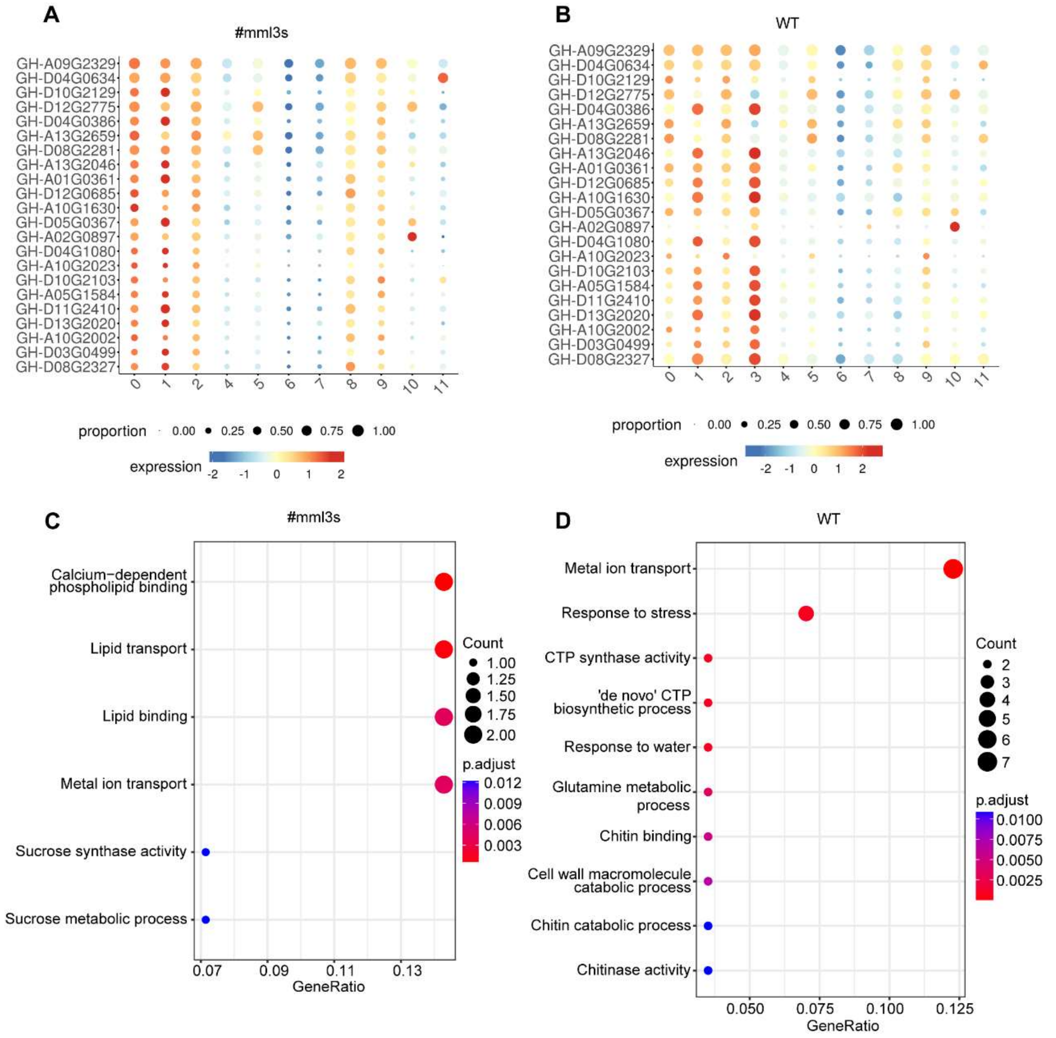

Supplemental Figure 18. Comparative expression of highly-expressed *#mml3s* Cluster 0 genes in *#mml3s* and WT samples.

(A) Expression of highly-expressed *#mml3s* Cluster 0 genes in *#mml3s* clusters. **(B)** Expression of the same genes in WT clusters. **(C)** GO enrichment of highly-expressed *#mml3s* Cluster 0 genes. **(D)** GO enrichment of highly-expressed WT Cluster 0 genes.

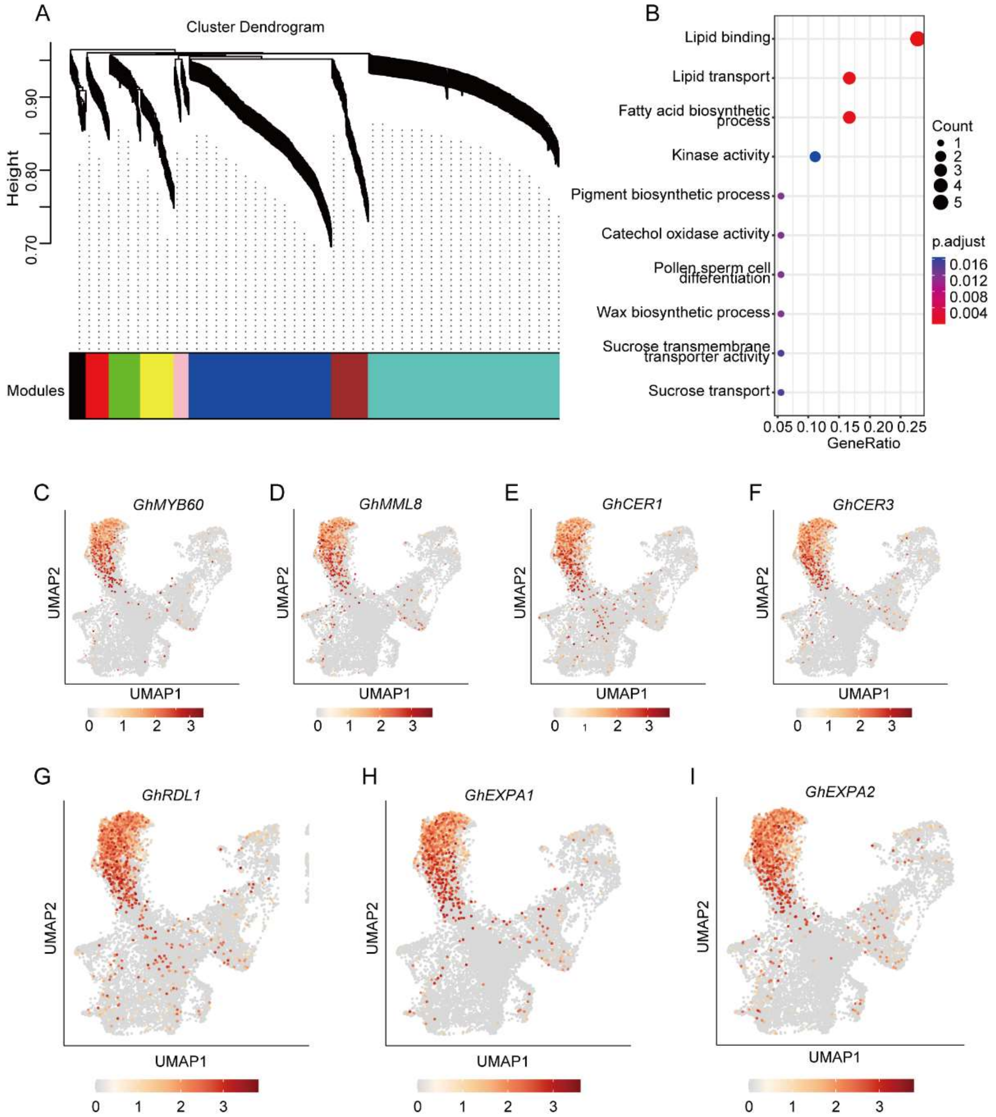

Supplemental Figure 19. Hierarchical cluster tree by WGCNA and expression profiles of fiber-related genes in #*mml3s* and WT samples.

(A) Hierarchical cluster tree showing co-expression modules identified by WGCNA. **(B)** GO enrichment analysis of genes in black module of WGCNA. **(C–F)** UMAP expression patterns of the potential fiber development-related genes *GhMYB60*, *GhMML8*, *GhCER1*, and *GhCER3* in WT and #*mml3s*. **(G–J)** UMAP plots for the fiber elongation-related genes *GhRDL1*, *GhEXPA1*, and *GhEXPA2* in WT and #*mml3s*.

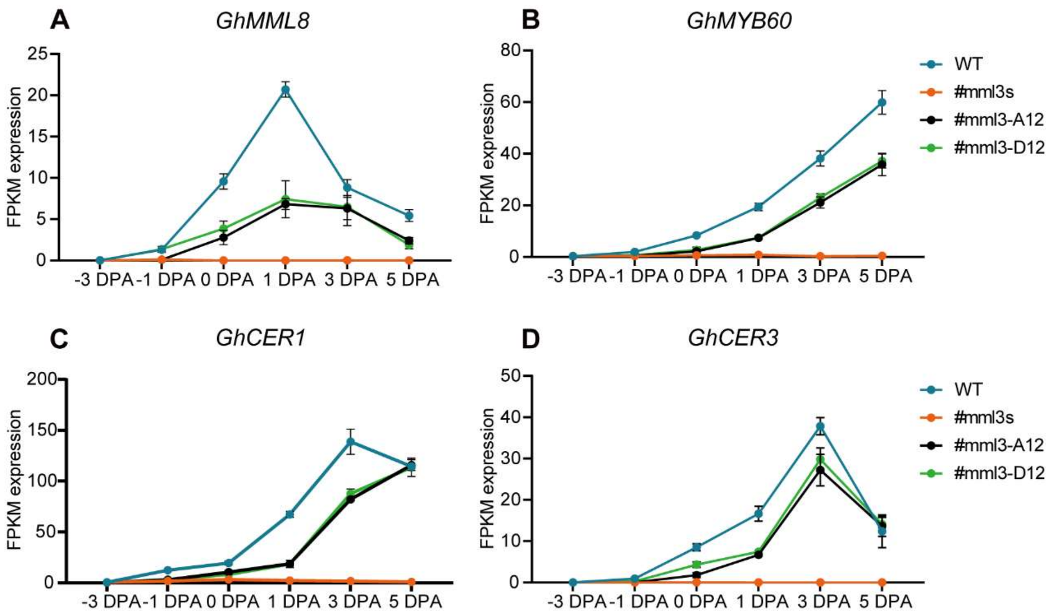

Supplemental Figure 20. Expression trends of potential fiber-related genes during fiber initiation and development.

Data are represented as means ± SEM of three biological replicates.

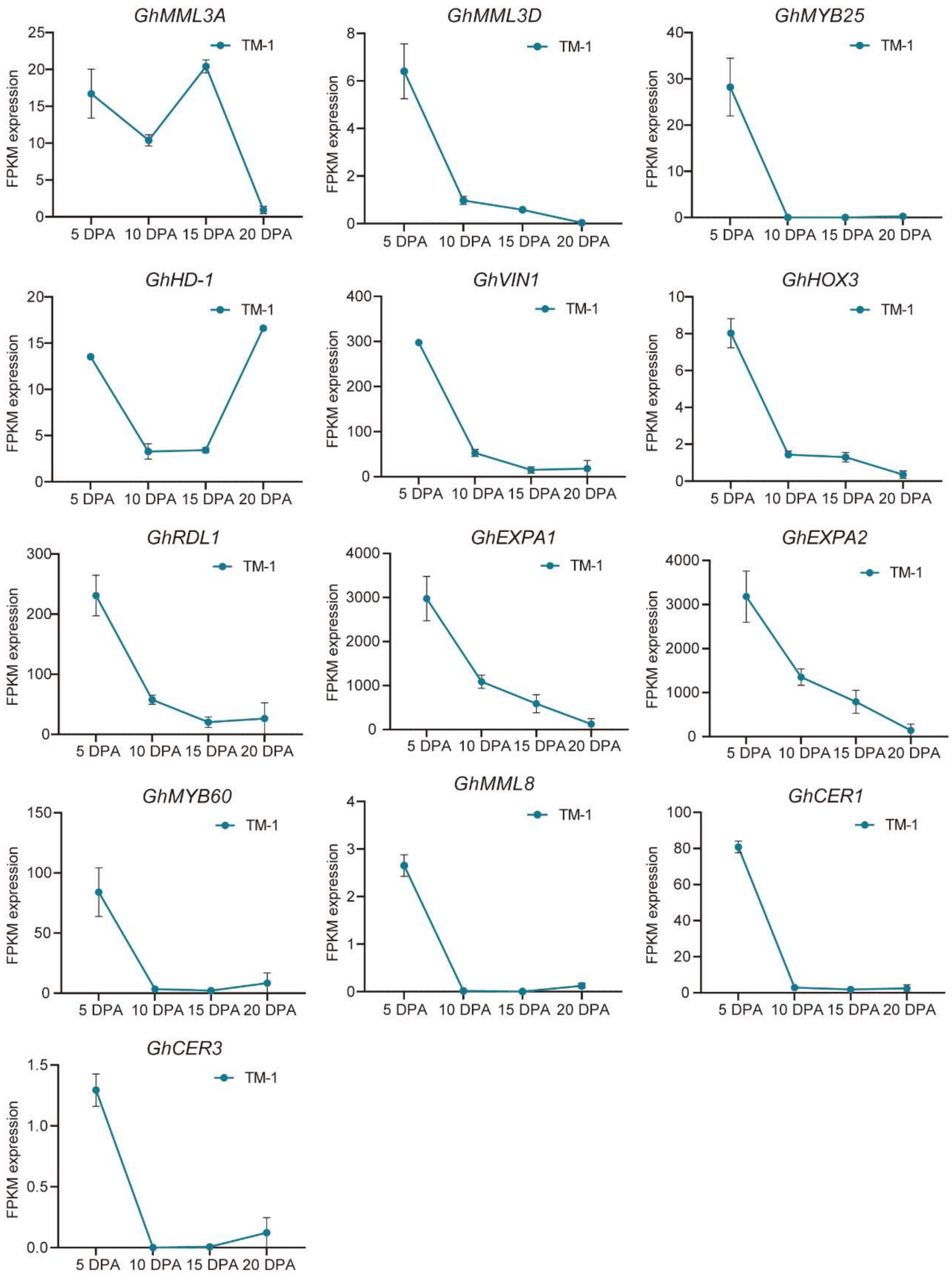

Supplemental Figure 21. Expression trends of fiber elongation-related genes during the rapid elongation period (5~20 DPA).

Data are represented as means ± SEM of three biological replicates.
