# Supplementary material for: Two duplicated GhMML3 genes coordinately control development of lint and fuzz fibers in cotton
Source: Plant Commun. 2025 Feb 12;6(4):101281. doi: 10.1016/j.xplc.2025.101281 (PMC12010375; doi:10.1016/j.xplc.2025.101281)
Supplement: Document S2. Article plus supplemental information [file mmc3.pdf]

Plant Communications
Research article

CellPress
Partner Journal

Two duplicated *GhMML3* genes coordinately control development of lint and fuzz fibers in cotton

Rui Chen[1,2,6], Jun Zhang[1,3,6], Jun Li[2], Jinwen Chen[1], Fan Dai[1], Yue Tian[4], Yan Hu[1,2], Qian-Hao Zhu[5] and Tianzhen Zhang[1,2,*]

[1]Zhejiang Provincial Key Laboratory of Crop Genetic Resources, Institute of Crop Science, Plant Precision Breeding Academy, College of Agriculture and Biotechnology, Zhejiang University, Hangzhou, China

[2]Hainan Institute of Zhejiang University, Sanya, Hainan 572025, China

[3]Institute of Horticulture, Zhejiang Academy of Agricultural Sciences, Hangzhou, Zhejiang, China

[4]College of Biotechnology, Jiangsu University of Science and Technology, Zhenjiang, China

[5]CSIRO Agriculture and Food, GPO Box 1700, Canberra, ACT 2601, Australia

[6]These authors contributed equally to this article.

***Correspondence: Tianzhen Zhang (cotton@zju.edu.cn)**

https://doi.org/10.1016/j.xplc.2025.101281

ABSTRACT

Cotton produces two types of fibers: fuzz and lint. Cotton yield is determined by the number of epidermal cells that develop into lint fibers. Despite numerous studies, the genetic and molecular mechanisms that control lint and fuzz fiber development remain unclear. Here, using the recessive naked-seed or fuzzless-linted mutant (n_2NSM) in combination with gene editing and complementation, we found that the recessive fuzzless gene n_2 encodes the MYBMIXTA-like (MML) transcription factor *GhMML3_D12*. Overexpression of *GhMML3_D12* in n_2NSM restored fuzz fiber development, whereas CRISPR-Cas9 knockout of *GhMML3_D12* in wild-type cotton (J668) resulted in a fuzzless-linted phenotype. Interestingly, simultaneous edits to *GhMML3_D12 and its* duplicate *GhMML3_A12* resulted in plants with a fiberless (fuzzless–lintless) phenotype. Detailed investigation of the seed fiber phenotypes of segregating progeny derived from a cross between J668 and a fiberless gene-edited mutant of *GhMML3* (*#mml3s*) not only identified progeny that mimicked natural fuzzless and fiberless mutants but also revealed that the duplicated *GhMML3_A12* and *GhMML3_D12* regulate the development of fuzz and lint fibers in a dose-dependent manner. Comparative transcriptome analysis and single-cell RNA sequencing identified *GhMML3* as the central hub of the gene network that regulates fiber initiation and early-stage elongation. The gene regulatory network revealed potential candidate genes and key regulators that may contribute to fiber initiation and development, and a model for the control of lint and fuzz fiber development by *GhMML3* was proposed. We also found that the GhMML3_D12 protein can bind directly to the promoters of *GhHD-1* and *GhMYB25*, two key genes involved in fiber initiation, thereby activating their expression. This study provides new insights into the fundamental mechanisms that underlie cotton fiber development.

Key words: cotton, fiber initiation, CRISPR-Cas9, GhMML3

Chen R., Zhang J., Li J., Chen J., Dai F., Tian Y., Hu Y., Zhu Q.-H., and Zhang T. (2025). Two duplicated *GhMML3* genes coordinately control development of lint and fuzz fibers in cotton. Plant Comm. **6**, 101281.

INTRODUCTION

Cotton is the most important fiber crop worldwide. Cotton seeds produce two types of fibers, lint and fuzz, and the final cotton fiber yield is determined by the number of seed epidermal cells that can potentially develop into lint fibers during the fiber initiation stage. Lint fibers originate from seed epidermal cells that differentiate into fiber initials before flowering (about –2 d from the day of flowering). The fiber initials protrude from the seed coat around the day of flowering, i.e., 0 days post anthesis (DPA), and continue to grow up to 2.5 to 3.5 cm. Fuzz fibers are initiated at 3 to 5 DPA and grow to only 5 to 10 mm (Stewart, 1975; Haigler et al., 2012; Wang et al., 2021a).

© 2025 The Author(s). Published by Elsevier Inc. on behalf of CAS Center for Excellence in Molecular Plant Sciences, Chinese Academy of Sciences, and Chinese Society for Plant Biology.
This is an open access article under the CC BY license (http://creativecommons.org/licenses/by/4.0/).

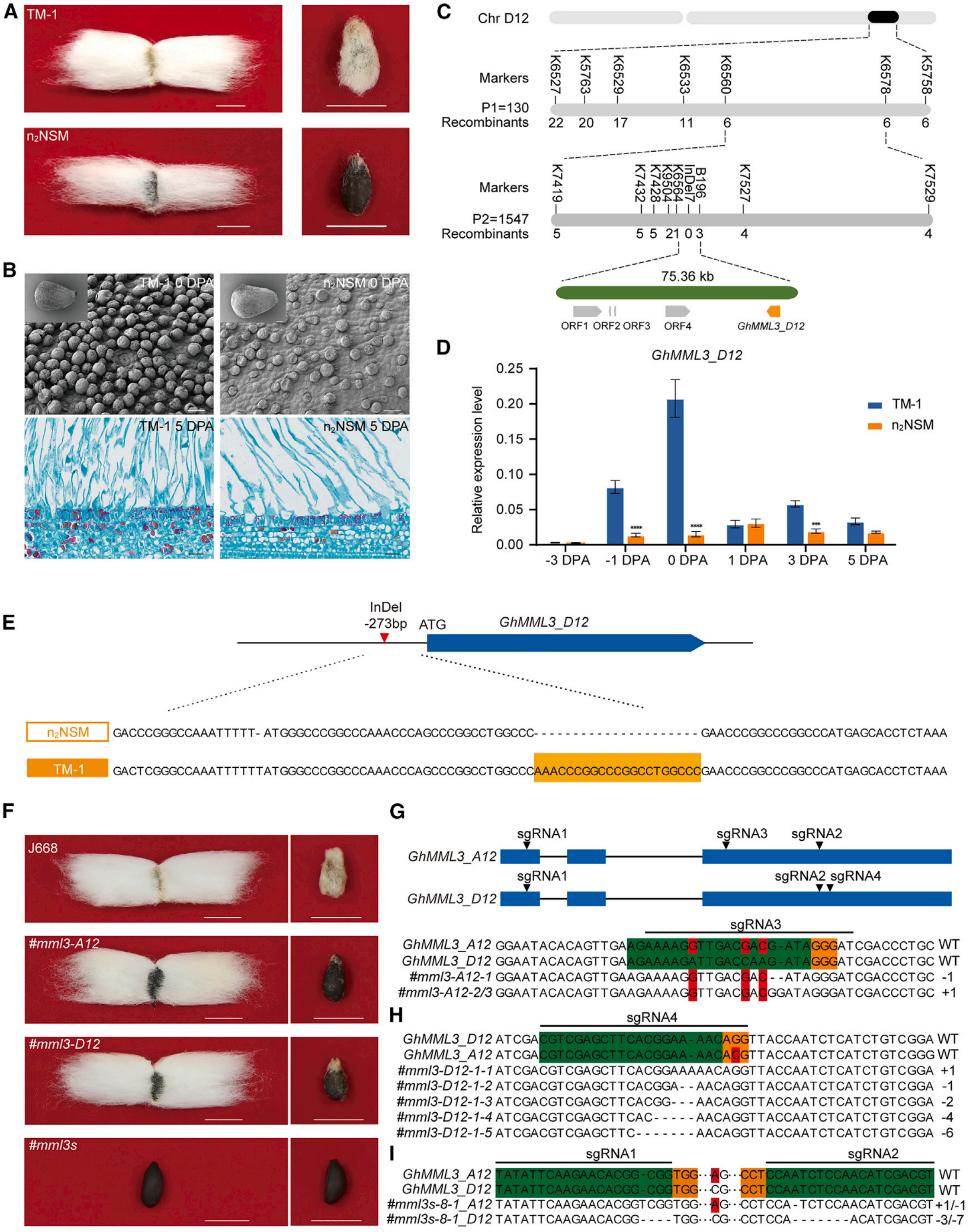

Figure 1. Map-based cloning of the recessive naked-seed gene n_2.

(A) Fuzzy and fuzzless seed phenotypes of TM-1 and n_2NSM. Scale bars, 1 cm.

(B) Scanning electron micrographs and paraffin sections of ovules from TM-1 and n_2NSM at 0 and 5 days post anthesis (DPA). Arrows indicate initiating fuzz fiber cells. Scale bars, 200 μm (top) and 20 μm (bottom).

(C) Fine mapping of the n_2 gene. n_2 was mapped to chromosome D12 between the markers K6564 and B196 using the F_2 and BC_1 populations. The final mapping interval was 75.36 kb and contained five open reading frames.

(D) Quantitative reverse transcription PCR determination of *GhMML3_D12* expression in the ovules of TM-1 and n_2NSM. Statistical significance was determined using Student's *t*-test. Data are presented as means ± SEM of three biological replicates. ****$p < 0.0001$ and ***$p < 0.001$.

(E) Sequence differences in the *GhMML3_D12* promoter between TM-1 and n_2NSM.

(legend continued on next page)

Although cotton fibers (which are seed trichomes) and *Arabidopsis* leaf trichomes are both single-celled plant hairs, cotton has developed a unique transcriptional regulatory network for fiber development (Serna and Martin, 2006; Tian and Zhang, 2021). Epidermal trichome development in *Arabidopsis* is primarily regulated by R2R3-MYB, basic helix-loop-helix (bHLH), and WD40 (WD40-repeat) transcription factors (TFs), which together form an MYB–bHLH–WD40 complex that activates the downstream homeodomain-leucine zipper (HD-ZIP) TF *GL2* to initiate development of epidermal hairs (Rerie et al., 1994; Guan et al., 2008; Zhao et al., 2008; Pattanaik et al., 2014; Wang et al., 2021b). In members of the Malvaceae, including cotton, subgroup 9 of the R2R3-MYB TFs, termed the MYBMIXTA-like (MML) TFs, is expanded and constitutes a Malvaceae-specific family that regulates differentiation of seed epidermal fiber cells (Stracke et al., 2001; Zhang et al., 2015). Transcriptome analysis has revealed stage-specific expression of 10 MML TFs during cotton fiber development, with *GhMML7* (*GhMYB25*) showing specific expression in cotton fibers (Zhang et al., 2015). Overexpression of *GhMYB25* enhances fiber yield, whereas its repression inhibits fiber development (Machado et al., 2009). *GhMYB25-like* (*GhMML3*) is highly expressed in ovules at around 0 DPA and plays a vital role in regulating the gene network that controls fiber initiation; RNA-interference-mediated reduction in *GhMML3* expression results in cotton seeds with a nearly fiberless (fuzzless–lintless) phenotype (Walford et al., 2011; Qin et al., 2022; Zhao et al., 2024). Positional cloning has revealed that *GhMML3_A12* leads to the dominant fuzzless–linted phenotype of the N_1NSM cotton mutant (Wan et al., 2016).

Many HD-ZIP TFs are also specifically expressed in ovules and fibers and have significant effects on fiber development (Zhang et al., 2010; Deng et al., 2012; Ding et al., 2020). *GhHD-1* has been reported to promote fiber development by regulating levels of ethylene and reactive oxygen species. Inhibition of its expression through RNA interference delays fiber initiation, whereas overexpression increases the number of fiber initials (Walford et al., 2012). GhHD-1 forms a complex with GhHOX3, regulating the expression of genes that encode cell-wall-loosening proteins, including *GhRDL1* and *GhEXPA1* (Shan et al., 2014). Previous studies have shown that the fiberless trait of the SL1-7-1 mutant is linked to loci containing dysfunctional *GhMYB25-like_At* (*GhMML3_A12*) and *GhHD-1_At* genes (Sun et al., 2024). As one of the most important genes that regulate fiber elongation, *GhHOX3* significantly affects fiber length when its expression is repressed or enhanced (Shan et al., 2014). In addition, multiple lines of evidence suggest that plant hormones, sugar signaling, and very-long-chain fatty acids all contribute significantly to fiber cell development (Beasley and Ting, 1973; Qin et al., 2007; Liao et al., 2009; Zhang et al., 2011; Wang et al., 2014; Huang et al., 2021; Yang et al., 2023).

It has been suggested that N_1 and n_2 are the key genes that control the fuzzless trait (Kearney and Harrison, 1927; Ware et al., 1947; Kohel, 1973b; Endrizzi et al., 1985). Three additional naked-seed genes, n_3, n_4^t, and N_5, have been proposed to participate in the regulation of fuzz development (Turley and Kloth, 2002; Bechere et al., 2012; Zhu et al., 2021). The dominant fuzzless N_1 gene has been shown to encode the MML transcription factor GhMML3_A12. Small RNAs are generated from *GhMML3_A12* owing to the presence of an antisense transcript at its 3′ region, leading to decreased *GhMML3_A12* expression and the production of naked seeds (Wan et al., 2016). By contrast, the n_2 gene is believed to be responsible for the recessive fuzzless phenotype (Zhu et al., 2018; Chen et al., 2020a). Notably, there is genetic interference between the genes that regulate development of fuzz and lint fibers; in the fiberless mutant XZ142FLM, n_2 has an epistatic effect on the gene li_3, which controls lint fiber (Zhang and Pan, 1991; Wu et al., 2018).

Currently, the identity of the recessive fuzzless n_2 gene remains unknown, despite many genetic mapping studies (Rong et al., 2005; Turley and Kloth, 2008; Song et al., 2010; Zhu et al., 2018; Simin et al., 2019; Chen et al., 2020a). In the present study, we aimed to clarify the identity of the n_2 gene and to investigate the genetic and molecular mechanisms underlying the initiation of lint and fuzz fibers. We demonstrated that the n_2 gene encodes the MML TF GhMML3_D12 and is the corresponding duplicate of *GhMML3_A12*. We showed that the duplicated *GhMML3s* (*GhMML3_A12* and *GhMML3_D12*) coordinately regulate the development of fuzz and lint fibers in a dose-dependent manner and act as hub genes regulating fiber initiation and coordinating early-stage fiber elongation.

RESULTS

Fine mapping of the n_2 recessive naked-seed gene

To fine-map the n_2 gene, we produced (n_2NSM × TM-1)F_2 (n = 4352) and (n_2NSM × TM-1) × n_2NSM BC_1 (n = 1005) populations by crossing the n_2NSM mutant with TM-1 (Supplemental Tables 1 and 2). Using these two mapping populations, we fine-mapped the fuzzless n_2 gene to a 75.36-kb region on chromosome D12 (between 49 398 271 and 49 475 436), anchored by two insertion/deletion (InDel) markers, K6564 and B196. This region contained five open reading frames, encoding an uncharacterized protein (*ORF1*), two tRNA methyltransferases (*ORF2* and *ORF3*), a phosphate transporter (*ORF4*), and an MML TF (*GhMML3_D12*, *ORF5*) (Hu et al., 2019) (Figure 1A–1C; Supplemental Figure 1). We then isolated the coding sequences of these *ORFs* from TM-1 and n_2NSM and performed a sequence comparison, which revealed no differences in the amino acids encoded by these genes between the two genotypes (Supplemental Figures 2–5). *ORF2* and *ORF3* were uniquely annotated in the ZJU assembly (Zhang et al., 2015; Wang et al., 2019; Yang et al., 2019; Chen et al., 2020b; Huang et al., 2020; Ma et al., 2021) and showed no expression in the transcriptomes of n_2NSM and TM-1 (Supplemental Figure 6; Supplemental Table 3). Quantitative reverse transcription PCR revealed different expression levels of

(F) Seed fiber phenotypes of individual gene-edited plants with mutations in *GhMML3-A12* alone (#*mml3-A12*), *GhMML3-D12* alone (#*mml3-D12*), or both *GhMML3-A12* and *GhMML3-D12* (#*mml3s*). Both of the #*mml3-A12* and #*mml3-D12* single mutants exhibited a fuzzless seed phenotype. Double-mutant plants (#*mml3s*) showed a fuzzless–lintless phenotype. Scale bars, 1 cm.

(G–I) Editing events in #*mml3-A12*, #*mml3-D12*, and #*mml3s-8-1*. The sgRNA target sites and protospacer adjacent motif (PAM) sequences are highlighted with green and orange backgrounds, respectively. Polymorphic nucleotides between *GhMML3-A12* and *GhMML3-D12* are highlighted in red. The numbers of nucleotide deletions or insertions caused by gene editing are shown on the right.

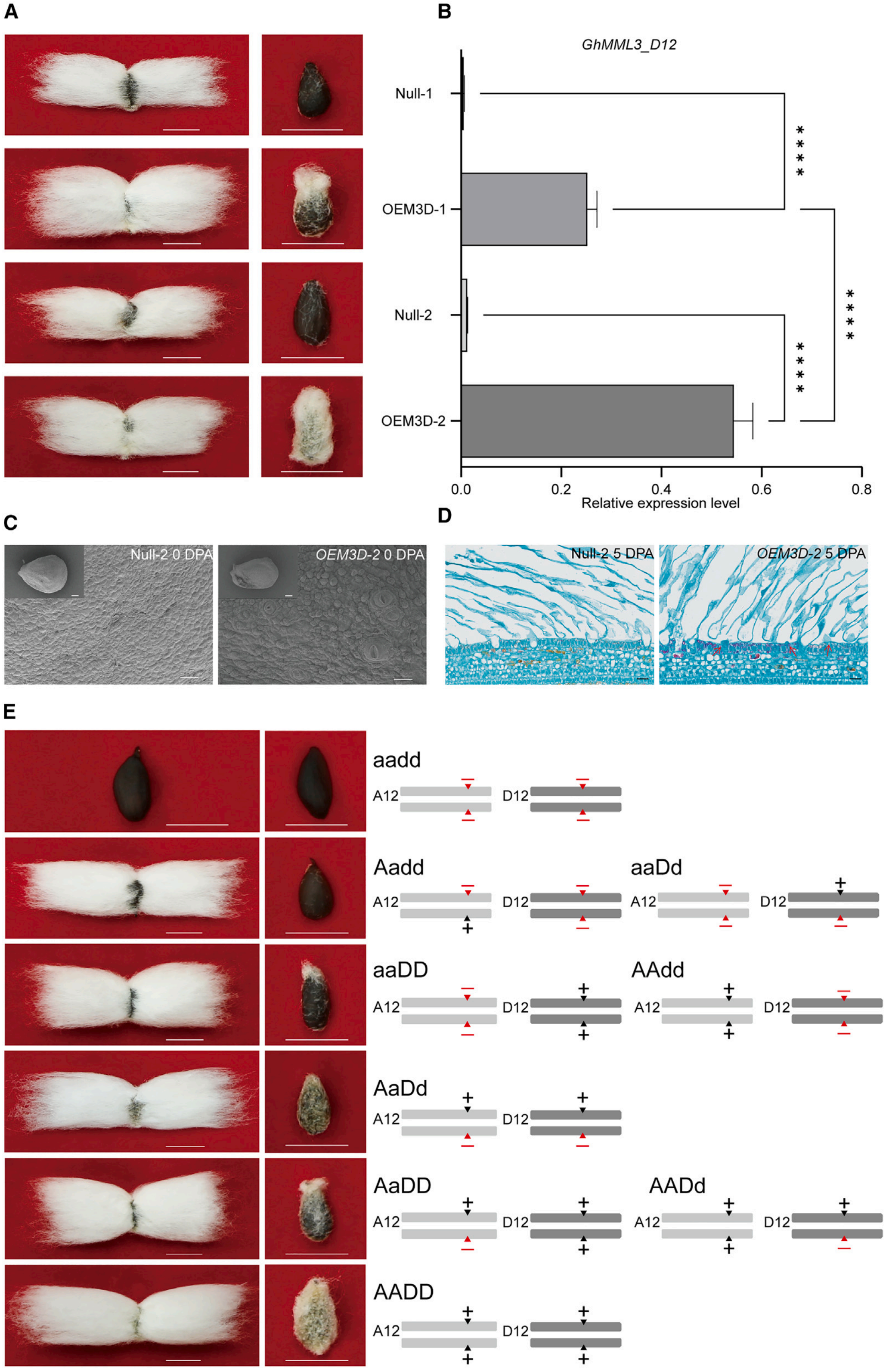

Figure 2. Functional characterization of the n_2 gene.
(A) Fiber phenotypes of transgenic overexpression lines with different levels of *GhMML3_D12*. Null denotes an individual negative for the transgene of the corresponding overexpression line. Scale bars, 1 cm.

(legend continued on next page)

ORF1 and *GhMML3_D12* in TM-1 and n_2NSM (Figure 1D; Supplemental Figure 6). An InDel between n_2NSM and TM-1 was identified in the promoter of *GhMML3_D12*, with the n_2NSM mutant lacking a 21-bp fragment at position −273 bp. This deletion co-segregated with the naked-seed phenotype observed in all 112 fuzzless individuals of the (n_2NSM × TM-1)F_2 population (Figure 1E; Supplemental Table 4). Given that our previous study reported that *GhMML3_A12*, the duplicated gene of *GhMML3_D12*, is responsible for fuzz fiber development (Wan et al., 2016), we deduced that *GhMML3_D12* is also involved in fuzz development and that the n_2 gene is a mutated allele of *GhMML3_D12* that causes the recessive naked-seed phenotype.

Knockout of *GhMML3_D12* produced a naked-seed phenotype

To confirm that *GhMML3_D12* was the causative gene for the naked-seed phenotype, we designed four single-guide RNAs (sgRNAs) targeting exons of the *GhMML3s* for CRISPR-Cas9-mediated gene editing. First, sgRNA1 and sgRNA2 were designed to target both of the duplicated *GhMML3s* (*GhMML3_A12* and *GhMML3_D12*) and generate the double mutant *#mml3s*. By contrast, sgRNA3 and sgRNA4 were designed to specifically target *GhMML3_A12* and *GhMML3_D12* to generate the single mutants *#mml3-A12* and *#mml3-D12*, respectively (Figure 1G; Supplemental Figure 7). The editing events in these mutants (*#mml3-A12*, *#mml3-D12*, and *#mml3s*) were characterized by high-throughput sequencing (Figure 1G–1I; Supplemental Table 5). All T0-generation *#mml3-A12* and *#mml3-D12* lines were repeatedly self-pollinated until the corresponding homozygous lines were obtained (Supplemental Figure 8). All *#mml3-D12* plants produced naked seeds, and the naked-seed phenotype was also observed in *#mml3-A12* plants (Figure 1F). These results indicated that *GhMML3_A12* and *GhMML3_D12* are each individually involved in fuzz fiber development. Scanning electron microscopy revealed significant differences in the number of fiber initials between *#mml3-D12 plants and the transgene recipient J668 at 0 DPA*. In addition, distinct fuzz fiber cell protrusions were observed in J668 but not in *#mml3-D12* or *#mml3-A12* at 3–5 DPA (Supplemental Figure 9A–9C). Taken together, these results indicate that loss of *GhMML3_D12* function leads to loss of fuzz fiber on the seed surface.

Overexpression of *GhMML3_D12* restored normal fuzz fiber development in n_2NSM

To confirm the regulatory function of *GhMML3_D12* in fuzz fiber development, we constructed an overexpression vector for *GhMML3_D12* under the control of the *CaMV*35S promoter and transformed it into the n_2NSM mutant to perform a complementation test. Of the 10 transgenic overexpression lines developed, two independent lines (*OEM3D-1* and *OEM3D-2*) were chosen for subsequent analysis. Expression of *GhMML3_D12* in the transgenic plants restored normal fuzz fiber development, in contrast to the corresponding null segregants (Null-1 and Null-2) of each line. Furthermore, the fuzzy phenotype of the two overexpression lines was restored to various degrees and was correlated with expression of the transgene (Figure 2A and 2B). In particular, although *GhMML3_A12* expression did not differ significantly in *OEM3D-1* and *OEM3D-2* (Supplemental Figure 10), line *OEM3D-2*, which showed higher expression of the transgene (*GhMML3_D12*) than line *OEM3D-1*, produced much more fuzz (Figure 2A). *OEM3D-2* also had significantly more lint fiber cells at 0 DPA than the corresponding null (Null-2), as well as detectable projections of fuzz fiber cells at 5 DPA, which were not observed in the null (Figure 2C and 2D; Supplemental Figure 9D). This complementation test demonstrated that *GhMML3_D12* is responsible for fuzz fiber development.

GhMML3 double-knockout mutants mimic natural fiberless mutants

To knock out the duplicated *GhMML3s* simultaneously, we developed seven transgenic *#mml3s* lines (*#mml3s*-1 to -8) by *Agrobacterium*-mediated transformation. All T0 generation *#mml3s* lines were repeatedly self-pollinated until the corresponding homozygous lines were obtained. We selected a marker-free and homozygous line, #mml3s-8-1, for subsequent analysis. High-throughput sequencing revealed that sgRNA1 produced a base insertion and sgRNA2 a base deletion in the A12 homeolog, whereas sgRNA1 produced three base deletions and sgRNA2 seven base deletions in the D12 homeolog (Figure 1I).

The *#mml3s* lines all exhibited a fiberless phenotype from the T1 to the T6 homozygous lines. Interestingly, transgenic plants showed the fuzzless–linted phenotype when either *GhMML3_A12* or *GhMML3_D12* was knocked out but the fiberless phenotype when both genes were knocked out (Figure 1F). At 0 DPA, the number of epidermal cell protrusions was significantly reduced in *#mml3-A12* and *#mml3-D12*, and protrusions were almost completely absent in *#mml3s*. At 3–5 DPA, WT (wild-type) plants showed some epidermal cell protrusions that would later develop into fuzz fiber, but there were none in the gene-edited materials (*#mml3s*, *#mml3-A12*, and *#mml3-D12*) (Supplemental Figure 9). These results correspond to the fuzz and lint fiber phenotypes of the gene-edited materials and provide further evidence that *GhMML3_A12* and *GhMML3_D12* are jointly involved in fuzz and lint fiber development. Moreover, these findings provide a genetic explanation for why the cross between N_1 and n_2 naked-seed mutants gave rise to the MD17 fiberless mutant (Turley and Kloth, 2002).

GhMML3 regulates fiber initiation in a dose-dependent manner

To explore how *GhMML3* coordinately regulates lint and fuzz fiber development, we examined the seed fiber phenotypes of

(B) Quantitative reverse transcription PCR of *GhMML3_D12* expression in the ovules of overexpression plants. Statistical significance was determined using one-way ANOVA. Data are presented as means ± SEM of three biological replicates. ****p < 0.0001.

(C and D) Scanning electron micrographs and paraffin sections of ovules from Null-2 and *OEM3D-2* at 0 and 5 DPA. Arrows indicate initiating fuzz fiber cells. Scale bars, 20 μm (bottom) and 200 μm (top).

(E) Fiber phenotypes of segregants from the (*#mml3s-8-1* × J668)F_2 population, with their corresponding genotypes at the duplicated *GhMML3s* shown on the right. For brevity, the wild-type alleles of *GhMML3_A12* and *GhMML3_D12* are denoted as "A" and "D", respectively, and their corresponding edited alleles are denoted as "a" and "d", respectively. Red triangles with a "–" on top indicate CRISPR/Cas9-induced mutations, and gray triangles with a "+" on top indicate the wild type. Scale bars, 1 cm.

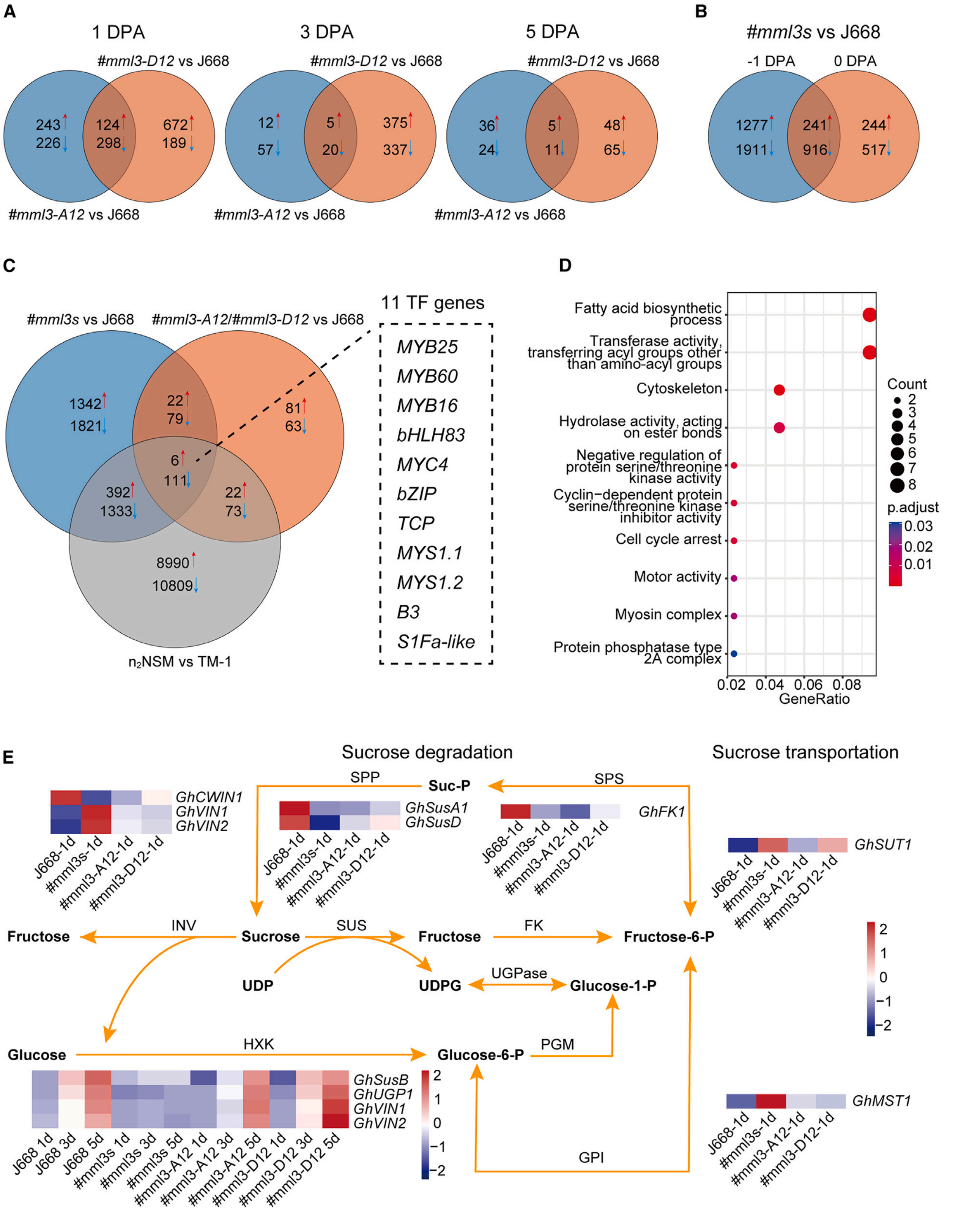

Figure 3. Bulk RNA-seq analysis.

(A) Venn diagrams of differentially expressed genes (DEGs) in *#mml3-A12* vs. J668 and *#mml3-D12* vs. J668 at 1, 3, and 5 DPA, $|\log_2(\text{FoldChange})| \geq 1$. The red arrows indicate upregulated DEGs, and the blue arrows indicate downregulated DEGs.

(B) Venn diagram of DEGs in *#mml3s* vs. J668 at −1 and 0 DPA, $|\log_2(\text{FoldChange})| \geq 1$.

(legend continued on next page)

the F_2 progeny from a cross between the gene-edited fiberless mutant (*#mml3s-8-1*) and the transgene recipient J668. For lint, the F_2 progeny could be separated into linted and lintless lines, but for fuzz, the F_2 progeny exhibited a complex and continuous segregation, from completely fuzzless to normal fuzzy. The overall seed fiber phenotypes of the F_2 progeny could be broadly grouped into normal fuzzy–linted, fuzzless–linted (with variable fuzz and tufts), and fuzzless–lintless (fiberless), but there were no fuzzy–lintless progeny. We investigated the genotypes of each representative type of phenotype by sequencing the duplicated *GhMML3s*. The fuzzy–linted individuals contained unmutated *GhMML3_A12* and *GhMML3_D12*, and the fiberless individuals had homozygous mutations in both *GhMML3_A12* and *GhMML3_D12* (designated *Ghmml3s*). The fuzzless–linted individuals contained at least one functional WT copy of either *GhMML3_A12* or *GhMML3_D12*, and the amount of fuzz appeared to be positively correlated with the number of functional WT copies of the duplicated *GhMML3s*. One interesting observation was that the tufted-seed phenotype seemed to be visible only in individuals with mutations in one or two of the four *GhMML3* copies but not in individuals with mutations in three or four of the copies (Figure 2E; Supplemental Figure 11; Supplemental Table 6). Together, these results suggest that the development of lint and fuzz fiber is determined by *GhMML3* through a dose-dependent mechanism.

GhMML3 regulates a network of genes responsible for fiber initiation

To further investigate the molecular mechanism by which *GhMML3* regulates lint and fuzz fiber initiation, we performed comparative transcriptome analysis of ovules (−1 to 5 DPA) from the gene-edited *GhMML3* mutants *#mml3-A12*, *#mml3-D12*, and *#mml3s* and the natural naked-seed mutant n_2NSM. The receptor line, J668, was used as the control. We identified 326 downregulated and 131 upregulated differentially expressed genes (DEGs) at 1–5 DPA in the two fuzzless–linted transgenic lines (*#mml3-A12* and *#mml3-D12*) (Figure 3A; Supplemental Table 7). In particular, these lines showed reduced expression of the fiber-initiation-related genes *GhHD-1* and *GhMYB25* (Supplemental Figure 13A and 13B). The results of quantitative reverse transcription PCR were consistent with the transcriptome data (Supplemental Figure 14). We further identified 3344 downregulated DEGs and 1762 upregulated DEGs at −1–0 DPA in *#mml3s* (Figure 3B; Supplemental Table 8). All of the downregulated DEGs identified at 1–5 DPA and −1–0 DPA were enriched for members of the fatty acid synthesis pathway and xyloglucan metabolism (Supplemental Figure 12B and 12D). There were 218 overlapping DEGs between 1–5 DPA and −1–0 DPA (Figure 3C). A comparison of these DEGs with the DEGs between n_2NSM and TM-1 identified in the 1–5-DPA ovules (Supplemental Table 9) yielded 117 overlapping DEGs (111 downregulated and 6 upregulated), which we considered to be candidate genes regulated by *GhMML3.* Eleven of these DEGs encoded TFs from seven families: MYB, bHLH, TCP, bZIP, G2-like, B3, and S1Fa-like (Figure 3C). These 117 DEGs were enriched in the Gene Ontology terms "fatty acid biosynthetic process," "transferase activity," and "cytoskeleton." The DEGs involved in fatty acid synthesis mainly encoded 3-ketoacyl-CoA synthases and fatty acid hydroxylases; they included *GhKCS2* (3-ketoacyl-CoA synthase 2, *GH_A10G1273/GH_D10G1602*), *GhKCS4* (3-ketoacyl-CoA synthase 4, *GH_D01G0047*), *GhKCS19* (3-ketoacyl-CoA synthase 19, *GH_A06G1931/GH_D06G1958*), *GhCER1* (ECERIFERUM1, *GH_A05G3496*), and *GhCER3* (ECERIFERUM3, *GH_A13G2356/GH_D08G2683*) (Figure 3D; Supplemental Table 10).

Genes associated with sugar metabolism are also reported to play a crucial role in the regulation of cotton fiber initiation (Wang et al., 2021a), and sugar signal transduction mediated by *GhVIN1* is clearly essential, because silencing *GhVIN1* by RNA interference results in a fiberless phenotype (Wang et al., 2014). Accordingly, we examined changes in the expression of genes associated with sucrose metabolism. At −1–0 DPA, the expression of *GhCWIN*, *GhSusA1*, *GhSusD*, and *GhFK1* decreased in *#mml3s*, whereas the expression of *GhVIN1*, *GhVIN2*, *GhMST*, and *GhSUT1* increased (Figure 3E). At 3–5 DPA, the expression of both *GhVIN1* and *GhVIN2* decreased in *#mml3s*, but there were no apparent differences in the expression of *GhCWIN*, *GhSusA1*, or *GhSusD* (Figure 3E; Supplemental Figure 15). Reduced expression of *GhVIN* may lead to a decrease in glucose, thus affecting carbohydrate metabolism and potentially influencing the subsequent development of ovule epidermal cells. Transcriptomic analysis further revealed a complex regulatory relationship between *GhVIN1* and *GhMML3*. Many of the genes differentially expressed in *#mml3s* at −1–0 DPA were related to fiber elongation, including *GhHOX3*, *GhRDL1*, *GhEXPA1*, and *GhEXPA2* (Figure 3B; Supplemental Tables 11 and 12). This result is consistent with the fact that, although fiber elongation follows fiber initiation, there is some overlap between the initiation and early elongation periods. Notably, *GhHOX3* is a core gene that controls early fiber elongation, and its expression was significantly reduced in *#mml3s* (Supplemental Figures 13C and 14F). These findings suggest that genes related to early fiber elongation function downstream of the *GhMML3s*.

Single-cell landscape of the cotton ovule epidermis

To further characterize the transcriptomic landscape at 0 DPA, a critical time point for fiber initiation, we performed a comparative single-cell RNA sequencing (scRNA-seq) experiment using 0-DPA ovules from the fiberless mutant (*#mml3s*). The 10× Genomics Chromium platform was used to capture and build the cDNA library. A total of 6222 cells were captured, with an average of 1259 genes per cell. After filtration, 6057 cells remained, accounting for 97.35% of the original cells (Supplemental Figure 16). Notably, the epidermal cells of *#mml3s* ovules exhibited no protrusions at 0 DPA. We integrated these single-cell data with line data from the WT, in this case the fuzzy-linted transgenic receptor line J668. After data integration, cell transcriptomic

(C) Venn diagrams of DEGs in *#mml3-A12/#mml3-D12* vs. J668 (1–5 DPA), *#mml3s* vs. J668 (−1–0 DPA), and n_2NSM vs. TM-1 (1–5 DPA). Transcription factors were identified among the 117 DEGs common to all three comparisons.

(D) Gene Ontology enrichment analysis of the 117 common DEGs.

(E) Expression profiles of genes related to sugar metabolism in different *GhMML3* gene-edited materials and the WT (J668).

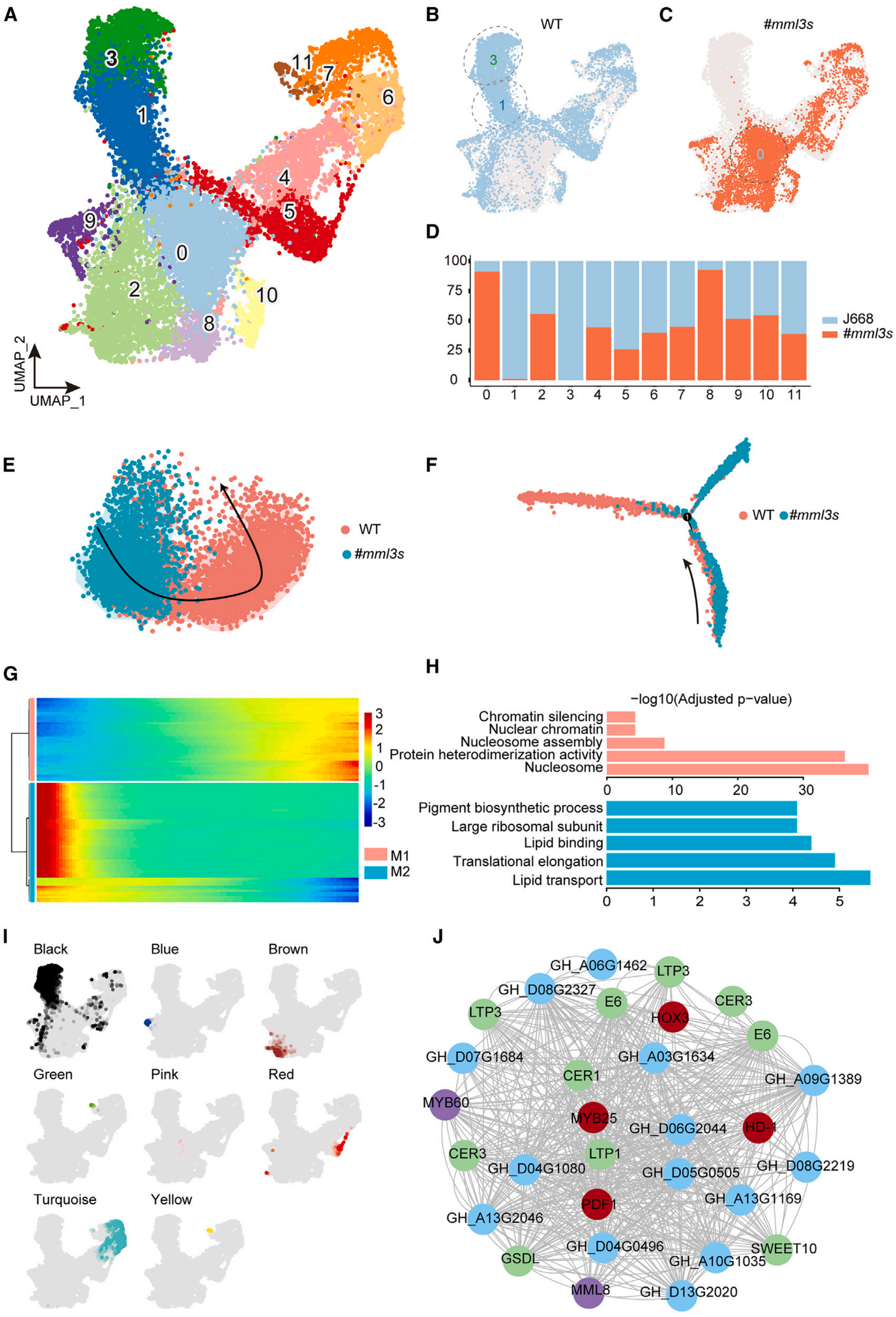

Figure 4. Single-cell landscape of developing ovules from WT and *#mml3s*.

(A) UMAP visualization of 12 clusters derived from the integrated analysis of 13 613 cells: 7566 from the WT and 6057 from *#mml3s*. Each dot denotes a single cell.

(B and C) Separate UMAP visualizations of clustering of the WT and *#mml3s* cells. Each dot denotes a single cell.

(legend continued on next page)

profiles were predicted in an unsupervised manner in the absence of marker genes, yielding 12 clusters, which were then visualized using the uniform manifold approximation and projection (UMAP) algorithm (Figure 4A).

Using marker-gene annotation results from previous studies, we annotated Clusters 1 and 3 as fiber cells and Clusters 0 and 8 as epidermal cells (Supplemental Figure 17A–17D). The numbers of cells in these clusters in WT and #*mml3s* were consistent with their fiber-initial phenotypes in the ovule epidermis at 0 DPA (Supplemental Figure 9). Compared with the WT, the fiberless mutant #*mml3s* had significantly fewer cells in Clusters 1 and 3 and significantly more cells in Clusters 0 and 8 (Figure 4B–4D). Further scoring based on cell-cycle genes identified Clusters 2 and 9 as proliferating cells (Supplemental Figure 17E and 17F). Specific examination of *GhMML3_D12* showed that it was differentially expressed between the WT and #*mml3s* in these clusters: the WT had higher *GhMML3_D12* expression in fiber cell Clusters 1 and 3, and #*mml3s* had higher *GhMML3_D12* expression in epidermal cell Cluster 0 (Supplemental Figure 17G–17J). Furthermore, the genes highly expressed in Cluster 0 of #*mml3s* were generally highly expressed in fiber cell clusters of the WT (Supplemental Figure 18; Supplemental Tables 13 and 14). We next used SCORPIUS and Monocle2 to analyze the developmental trajectories of cells in Clusters 0, 1, and 2 in both WT and #*mml3s* and of cells in Cluster 3 in the WT only. The developmental trajectory of #*mml3s* cells was arrested at the intermediate stage, unlike that of WT cells (Figure 4E and 4F). Enrichment analysis of biological process GO terms in #*mml3s* Cluster 0 highlighted terms linked to fiber growth, including "lipid transport," "lipid binding," "sucrose synthase activity", and "sucrose metabolic process," whereas a corresponding analysis of WT Cluster 0 revealed enrichment of the term "response to stress" (Supplemental Figure 18C and 18D; Supplemental Tables 15 and 16). Therefore, we postulate that a specific group of epidermal cells may begin differentiating into fiber cells in the 0-DPA ovules of #*mml3s* but experience disruptions during protrusion caused by loss of GhMML3 function. We constructed a gene-expression heatmap for cells at different differentiation states using fiber cell Clusters 1 and 3 as well as the early state of Cluster 2. These were divided into two modules, M1 and M2 (Figure 4G; Supplemental Table 17). GO enrichment analysis revealed that the M1 module, representing early proliferative cells, was enriched in pathways such as "nucleosome," "protein heterodimerization activity," and "nucleosome assembly," indicating that the cells were undergoing rapid division (Figure 4H). Genes from the M2 module were highly expressed in fiber cell clusters, showing early expression of genes associated with fiber initiation. Genes in the M2 module were primarily enriched in processes related to "lipid binding and transport," "translation elongation," and "large ribosomal subunit," and they included lipid transfer proteins (*GhLTP1* and *GhLTP3*) and lipid metabolism-related enzymes (*GhE6*), indicating a series of transcriptional activations (Figure 4H). This suggests an increase in protein translation/synthesis in fiber cells that prepares them for protrusion and elongation. The TF genes *GhMYB25* and *GhPDF1* were also found in the M2 module, and both have been found to play roles in fiber initiation and development (Machado et al., 2009; Deng et al., 2012).

To explore the gene regulatory network involved in fiber initiation and development, we performed weighted gene co-expression network analysis (WGCNA), which identified eight gene modules (Supplemental Figure 19A; Supplemental Table 18). We primarily focused on fiber cells and found that they clustered into the black module (Figure 4I). GO enrichment analysis revealed that the black module was enriched in "lipid binding," "lipid transport," "fatty acid biosynthetic process," and "sucrose transport," consistent with our previous findings that lipid and sucrose metabolism are associated with fiber initiation and development (Supplemental Figure 19B). Genes from the black module were used to construct a gene co-expression regulatory network (Figure 4J; Supplemental Table 19). This network included several key TFs, including *GhMYB25*, *GhHD-1*, *GhPDF1*, and *GhHOX3*, which have been reported to participate in fiber initiation and development (Machado et al., 2009; Deng et al., 2012; Shan et al., 2014; Ding et al., 2020). It also contained lipid transfer proteins (*GhLTP1* and *GhLTP3*), enzymes related to lipid metabolism (*GhCER1*, *GhCER3*, *GhE6*, and *GhGSDL*), and a sucrose transporter protein (*GhSWEET10*). The GhE6 protein has been reported to participate in regulating the diffuse growth of fiber cells, and silencing of *GhSWEET10* leads to shorter fibers, whereas overexpression of *GhSWEET10* leads to longer fibers (Qin et al., 2022; Du et al., 2024). In addition, the network included *GhMYB60* and *GhMML8*, TFs that also appeared in the bulk RNA-seq data. Other genes found in the regulatory network included *SVB-LIKE* (*GH_A13G2046*/*GH_D13G2020*), whose loss of function in *Arabidopsis* leads to reduced trichome numbers and abnormal branching development (Yu et al., 2021) (Figure 4J). The gene regulatory network thus provides important candidate genes for the study of fiber initiation and development.

Guided by the newly constructed gene regulatory network, we next focused on specific DEGs within cell clusters. Differentially expressed TFs in the fiber cell clusters included the fiber-initiation genes *GhMYB25* and *GhHD-1*, as well as *GhMYB60* and *GhMML8* (Supplemental Figures 17K and 17L and 19C and 19D). The fatty acid biosynthesis-related genes *GhCER1* and *GhCER3* were differentially expressed in Cluster 3

(D) Proportions of WT and #*mml3s* cells in each cluster.
(E) SCORPIUS trajectory analysis showing the relationships between the WT and #*mml3s* (Clusters 0, 1, and 2 from #*mml3s* and Clusters 0, 1, 2, and 3 from the WT).
(F) Monocle2 trajectory analysis showing the relationships between the WT and #*mml3s* (Clusters 0, 1, and 2 from #*mml3s* and Clusters 0, 1, 2, and 3 from the WT).
(G and H) Gene-expression trends of cells based on different differentiation states along the developmental trajectory. On the right, significantly enriched GO terms for each cluster are shown.
(I) UMAP plots showing the Module Eigengene (ME) coloring of gene co-expression modules in WGCNA.
(J) Gene regulatory network of the black module related to fiber cell development. Nodes are colored differently to distinguish the genes.

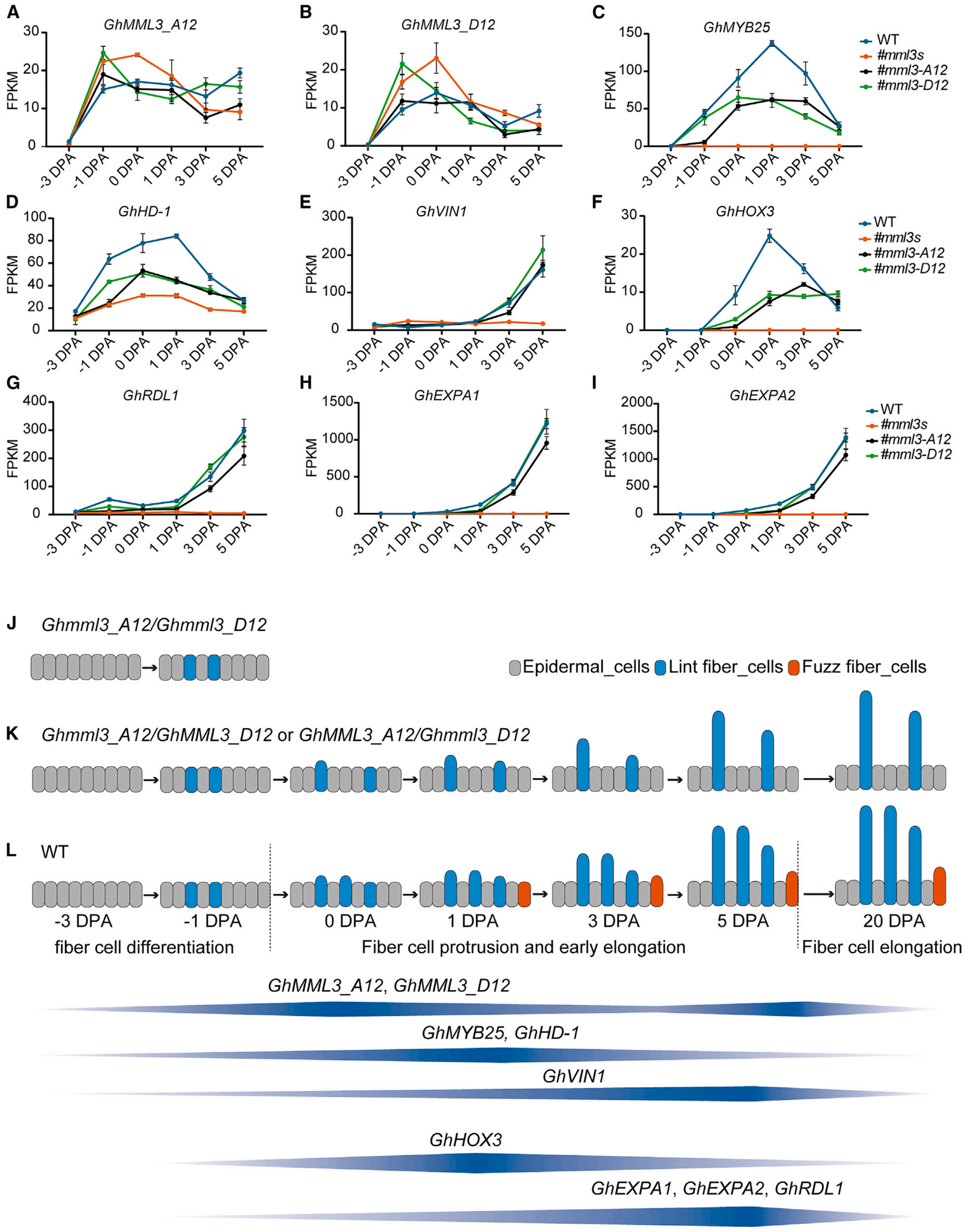

Figure 5. GhMML3-mediated regulation of fiber cell initiation.
(A–I) Expression trends of genes related to fiber initiation and elongation in WT and *GhMML3* gene-edited materials from −3 to 5 DPA. Data are presented as means ± SEM of three biological replicates.

(legend continued on next page)

(Supplemental Figure 19E and 19F). The sugar-metabolism gene *GhVIN1* was specifically expressed in fiber cells (Supplemental Figure 17M), and its differential expression at the fiber-initiation stage was verified in the bulk RNA-seq data (Figure 3E). The fiber elongation-related genes *GhHOX3*, *GhRDL1*, *GhEXPA1*, and *GhEXPA2* were also differentially expressed in Cluster 3 (Supplemental Figures 17N and 18G–18I). *GhHOX3* is reportedly a key factor in the regulation of fiber elongation, and *GhRDL1* and *GhEXPA* function downstream of *GhHOX3* (Shan et al., 2014). Our data showed that *GhHOX3* expression and function began after the development of fiber cell protrusions at 0 DPA (Supplemental Figures 13C and 14F). We speculated that GhMML3 might regulate fiber development by influencing the expression of these genes, and we therefore analyzed their expression patterns during fiber initiation and development. *GhMML3* expression increased from −1 to 0 DPA, then began to decrease, only to increase again from 3 to 5 DPA (Figure 5A and 5B; Supplemental Figure 14). This expression pattern corresponded to the peak periods of fiber cell protrusion. *GhMYB25* and *GhHD-1* both increased in expression from −1 to 1 DPA and then decreased, whereas *GhVIN1* showed an increasing trend from −1 to 5 DPA (Figure 5C–5E; Supplemental Figure 14). *GhMML8* and *GhCER3* had expression trends similar to that of *GhMYB25*, and the expression profiles of *GhMYB60* and *GhCER1* were similar to that of *GhVIN1* (Supplemental Figure 20). Expression of *GhRDL1*, *GhEXPA1*, and *GhEXPA2* increased from 0 to 5 DPA (Figure 5G–5I; Supplemental Figure 14). In the *GhMML3* mutants (both single and double), expression of these genes was reduced to different extents (Supplemental Figures 13 and 14). Bulk RNA-seq data revealed that expression of *GhHOX3*, a key gene for early fiber elongation, began at 0 DPA, reached its peak at 1 DPA, and then decreased continuously until 5 DPA (Figure 5F; Supplemental Figure 14). According to our previously reported RNA-seq data (Hu et al., 2019), its expression continued to decrease until 20 DPA (Supplemental Figure 21). The gradually attenuated expression of genes that contribute to early fiber elongation, such as *GhHOX3*, from 3 DPA onward may promote the formation of fuzz fibers but may simultaneously restrict their elongation, assuming that the early elongation stages of both lint and fuzz fibers are regulated by the same set of genes, including *GhHOX3*.

GhMML3_D12 directly binds to the promoters of *GhHD-1* and *GhMYB25*

Our transcriptome and single-cell data confirmed that *GhHD-1* and *GhMYB25* are specifically expressed in fiber cells and are potential downstream targets of *GhMML3*. We therefore performed experiments to investigate the transcriptional activation of *GhMYB25 and GhHD-1* by GhMML3_D12. We analyzed the promoter regions of *GhHD-1* and *GhMYB25* and identified at least one MYB binding *cis*-element within the 2-kb upstream regions of their transcription start sites. We then examined the binding of GhMML3_D12 in an electrophoretic mobility shift assay (EMSA). The EMSA showed that GhMML3_D12 formed stable complexes with promoter probes of *GhMYB25* and *GhHD-1*. By contrast, when GhMML3_D12 was incubated with a mutated biotinylated probe, this binding was almost completely abolished (Figure 6A and 6B). Next, we performed a dual-luciferase transient expression assay in which a reporter gene was co-transformed with 35S::GhMML3_D12 into *Nicotiana benthamiana* leaves. Co-transfection with GhMML3_D12 enhanced the expression of the luciferase reporter gene driven by the *GhHD-1* or *GhMYB25* promoter sequence, generating a stronger fluorescence signal (Figure 6C). Recent studies have shown that RNAi-mediated silencing of *GhHD-1* or *GhMYB25* inhibits cotton fiber initiation and development (Machado et al., 2009; Walford et al., 2012). Taken together, these results indicate that GhMML3_D12 directly binds to the promoters of *GhMYB25* and *GhHD-1*, positively regulating their expression and thus controlling fiber initiation and development.

DISCUSSION

Duplicated *GhMML3s* coordinately regulate lint and fuzz fiber development

Cotton seeds have two types of fiber: lint and fuzz. Lint fiber holds significant value as a basic material in the textile industry (Stewart, 1975; Haigler et al., 2012), whereas the commercial value of fuzz fiber is less important. Both lint and fuzz develop from seed epidermal cells, with protrusions at −1–0 DPA forming lint fibers and protrusions at 3–5 DPA forming fuzz fibers. After initiation, the lint fiber progressively elongates, a developmental step that overlaps with the initiation of fuzz fiber. Fuzzless cotton has several commercial advantages, including easier fiber processing, reduced pathogen transmission by seeds, suitability for machine planting, rapid water absorption, high germination rates, and ease of purity assessment. The fuzzless trait is primarily controlled by two independent loci, the dominant gene N_1 and the recessive gene n_2. The N_1 gene has been identified as *GhMML3_A12* (Wan et al., 2016). Crossing N_1 with n_2 produces fiberless progeny (Turley and Kloth, 2002). Recently, a new n_3 locus has been identified, and the N_3 locus was found to have an epistatic effect on expression of the n_2 locus (Turley and Kloth, 2002). However, there is currently no consensus on the identity of the recessive gene n_2. Here, the n_2 gene was identified as encoding the MML TF GhMML3_D12 through map-based cloning, a result further verified by knockout of *GhMML3_D12* using gene editing and overexpression of *GhMML3_D12* in n_2NSM. The *GhMML3* mutants generated by CRISPR-Cas9-mediated gene editing revealed that *GhMML3_A12* and *GhMML3_D12* control lint and fuzz fiber development in a dose-dependent manner and that the fiberless phenotype is observed only when both *GhMML3_A12* and *GhMML3_D12* are mutated, consistent with the results of previous

(J–L) Epidermal cells first differentiate into precursor fiber cells, which protrude in response to increased expression of *GhMML3.* Precursor fiber cells primarily undergo continuous protrusion from −1 to 5 DPA. Expression of *GhMML3* increases at −1 to 0 DPA and 3 to 5 DPA, in line with the first (lint) and second (fuzz) waves of fiber cell protrusion. *GhMYB25*, *GhHD-1*, and *GhVIN1* are regulated by *GhMML3* and participate in regulating the initiation of fiber cells. *GhHOX3* expression begins after 0 DPA, regulating the early post-protrusion elongation of fiber cells. *GhEXPA1*, *GhEXPA2*, and *GhRDL1* function downstream of *GhHOX3* to promote further fiber elongation. Knockout of either *GhMML3_A12* or *GhMML3_D12* in the WT results in insufficient *GhMML3* expression during the 3-to-5-DPA period, failing to promote protrusion and ultimately leading to a fuzzless seed phenotype.

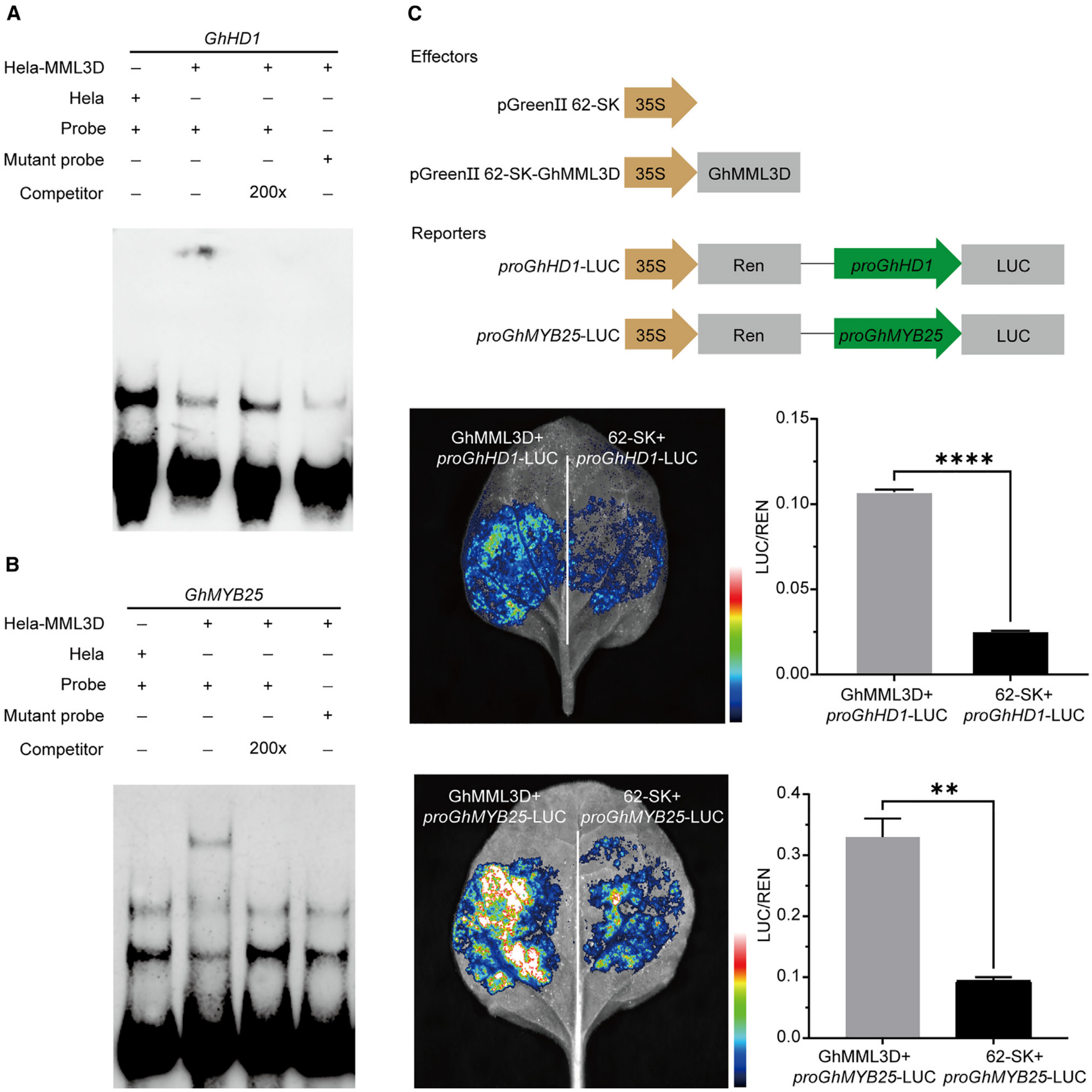

Figure 6. GhMML3_D12 directly regulates the expression of *GhHD-1* and *GhMYB25*.
(A and B) EMSAs of the interactions between the HeLa-GhMML3_D12 recombinant protein and the promoter fragments. Probe, biotin-labeled probe with an intact binding motif; competitor, unlabeled DNA probe with an intact binding motif; mutant probe, biotin-labeled probe with a mutated binding motif.
(C) Transient dual-LUC assay showing that GhMML3_D12 induces transcription of the *GhHD-1* and *GhMYB25* promoters. The expression level of REN was used as an internal control. The LUC/REN ratio represents the relative activity of the promoter. The data are presented as the means ± SEM of three biological replicates. ****$p < 0.0001$ and **$p < 0.01$; Student's *t*-test.

genetic analysis using natural fiber mutants. The results also confirm the epistatic interaction between *GhMML3_A12* and *GhMML3_D12* in the determination of lint and fuzz fiber development (Zhang and Pan, 1991; Wu et al., 2018). That is, if either *GhMML3_A12* or *GhMML3_D12* is functional, cotton seeds will always produce lint fiber (Figure 7).

Cotton is an allotetraploid species that originated approximately 1–2 million years ago from hybridization of A-genome and D-genome diploid cotton species (Flagel et al., 2012; Bao et al., 2019). In allotetraploid species, duplicated genes have multiple fates: defunctionalization, subfunctionalization, or neofunctionalization. Many duplicated genes, including TF genes, are dose sensitive (Grover et al., 2012; Birchler and Yang, 2022). Hypofunction involves reducing the expression of both copies of a duplicate pair to a threshold level, such that both copies are required for the specific function of the gene and are therefore both maintained (You et al., 2023). In cotton,

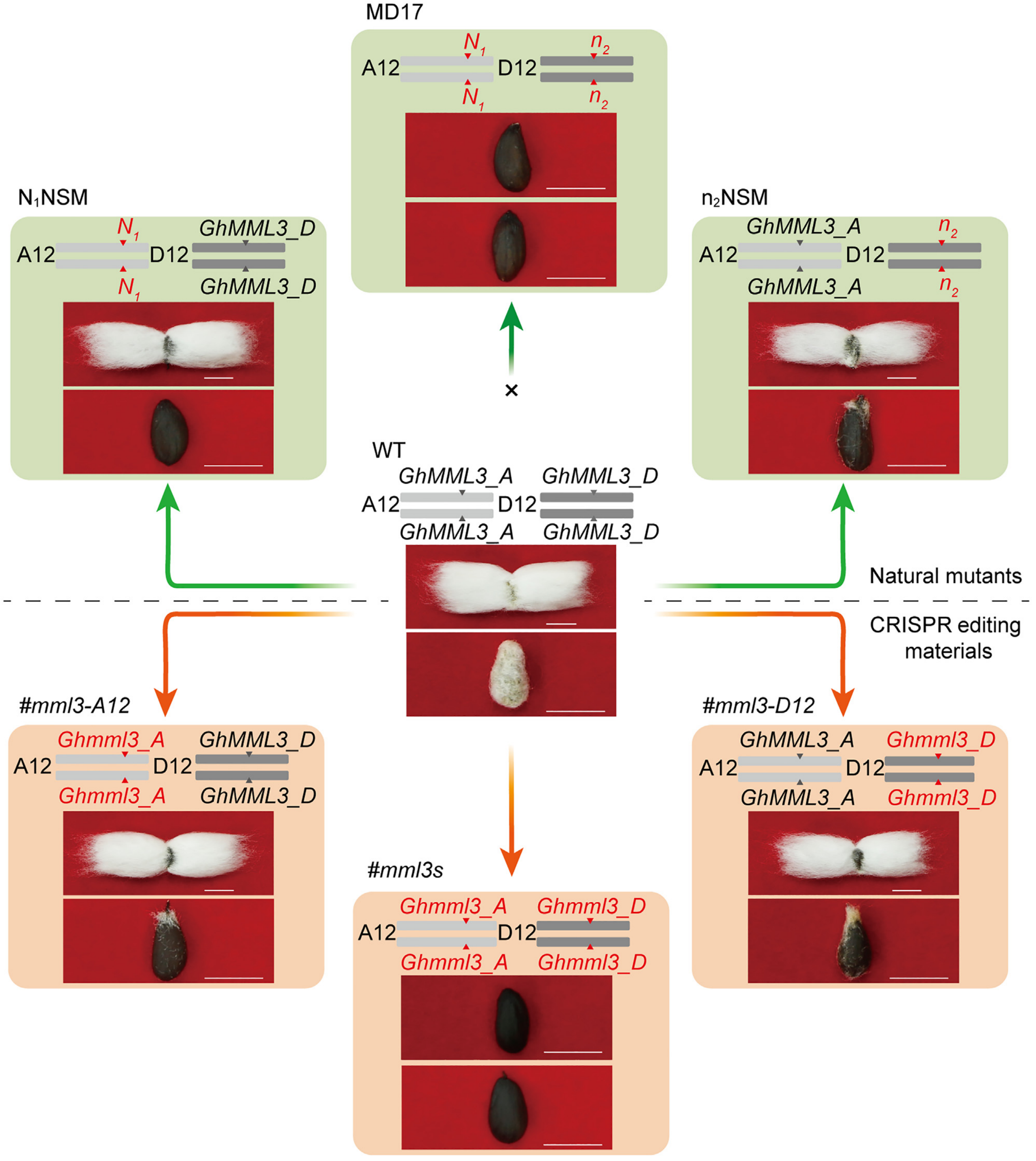

Figure 7. Gene-edited mutants of *GhMML3* mimic natural fuzzless and fiberless mutants.
Mutation of both copies of *GhMML3_A12* (*#mml3-A12*) mimics the dominant fuzzless mutant (N_1NSM), whereas mutation of both copies of *GhMML3_D12* (*#mml3-D12*) mimics the recessive fuzzless mutant (n_2NSM). Mutation of both *GhMML3_A12* and *GhMML3_D12* (*#mml3s*) mimics the fiberless mutant MD17. WT alleles are shown in black and mutated alleles in red.

72 761 and 75 071 high-confidence protein-coding genes have been predicted for *Gossypium hirsutum* acc. TM-1 and *G. barbadense* cv. Hai7124, respectively. Most of these genes, 93.6% in TM-1 and 93.8% in Hai7124, have been retained as duplicated copies, largely owing to the species' allotetraploid nature (Hu et al., 2019). That is, duplicated genes mutate in different regulatory regions and are then stabilized through random genetic drift, causing the gene pair to complement each other and maintain their preduplication expression level, thereby fulfilling the functions once fulfilled by the diploid ancestral gene.

Multiple pairs of duplicated genes have been reported and cloned in cotton, including the open bud genes ob_1ob_2, green mutations (v_5v_6, $v_{16}v_{17}$), the nectary genes Ne_1Ne_2, the axillary cluster flowering genes Cl_1Cl_2, and the male sterility genes ms_5ms_6 (Kohel, 1973a, 1983; Qian et al., 2009; Pei et al., 2021; Mao et al., 2023; Zhang et al., 2023). In the present study, the duplicated genes *GhMML3_A12* and *GhMML3_D12* were found to jointly regulate development of lint and fuzz fiber, exhibiting a pattern similar to that of the duplicated genes underlying the absence of gland pigments (gl_2gl_3) (Ma et al., 2016). One interesting observation was that the seed fiber phenotype of F_2 individuals with heterozygous mutations in both *GhMML3_A12* and *GhMML3_D12* (AaDd) appeared to differ from that of individuals with homozygous mutations in either *GhMML3_A12* or *GhMML3_D12* (aaDD or AAdd): the former had more fuzz fiber remaining on the seed surface than the latter (Figure 2E). This implies that *GhMML3_A12* and *GhMML3_D12* may have subtle functional differences that warrant further investigation. Further cloning and study of these duplicate genes will improve our understanding of the genetic and molecular mechanisms that govern gene expression in allopolyploid cotton.

Unraveling the genetic basis and regulatory networks related to fiber development

Many genes have been reported to participate in the regulation of cotton fiber initiation, such as *GhHD-1* and *GhMYB25*, as well as in the regulation of fiber elongation, such as *GhHOX3*, *GhEXPA1*, and *GhRDL1*. The expression of these genes was reduced in the *GhMML3* gene-edited materials (Supplemental Figure 13; Supplemental Tables 7 and 8). RNAi-mediated inhibition of *GhHD-1* delays fiber initiation, whereas its overexpression increases the number of fiber initials (Walford et al., 2012). Recent studies link the fiberless trait of SL1-7-1 to dysfunctional *GhMYB25-like_At* (*GhMML3_A12*) and *GhHD-1_At* loci (Sun et al., 2024). Overexpression of *GhMYB25* increases the number of fiber initials, whereas suppression of *GhMYB25* leads to shorter fibers (Machado et al., 2009). EMSA and luciferase reporter assays demonstrated that *GhMYB25-like_At* directly binds to the promoters of *GhHD-1* and *GhMYB25*, thereby regulating their expression. Our results demonstrate that *GhHD-1* and *GhMYB25* are directly regulated by *GhMML3* and are key genes in the *GhMML3* regulatory network that governs fiber initiation. Fatty acid biosynthesis was the most down-regulated pathway during fiber initiation and elongation, and genes related to this process also showed reduced expression in #*mml3s* (Supplemental Figure 13G–13J). *GhVIN1* exhibited an unusual expression pattern in the gene-edited materials, increasing during lint fiber initiation and decreasing during fuzz fiber initiation, rather than exhibiting a uniform upward or downward trend. There may be complex feedback regulatory relationships between *GhVIN1* and *GhMML3*. These results suggest that *GhMML3* is a hub gene located upstream in the fiber-development network, ultimately controlling fiber initiation by regulating multiple downstream pathways, including fatty acid biosynthesis and sugar metabolism.

Using scRNA-seq data generated from #*mml3s* and WT plants, we examined the specific expression of the above genes in fiber cells at the cellular level. Expression analyses revealed that the expression pattern of *GhMML3* was consistent with the protrusion of fiber cells. Differential expression of *GhHOX3* in #*mml3s* and WT fiber cells was also observed at 0 DPA. Gene-edited mutants of *GhHOX3* have been reported to show fuzzless–lintless phenotypes, but protrusions of fiber cells can be observed at 0 DPA (Qin et al., 2022). This indicates that *GhHOX3* is the key in determining the early elongation of fiber cell protrusions. *GhHOX3* expression reaches its peak at 1 DPA, then decreases rapidly during the subsequent fiber elongation period, an expression pattern that may be related to the formation of fuzz fibers. Scanning electron microscopy showed an absence of fiber cell protrusions in #*mml3s* at 0 DPA. Analysis of the developmental trajectory of scRNA-seq data revealed that #*mml3s* cells were arrested at an intermediate stage, and genes highly expressed in #*mml3s* epidermal cells were also found in WT fiber cells. These results suggest that epidermal cells in #*mml3s* may have differentiated as precursor fiber cells but were unable to protrude and elongate owing to loss of *GhMML3* function, implying that *GhMML3* is not involved in the differentiation of fiber cells. We constructed a gene regulatory network using WGCNA and identified several previously reported TF genes, such as *GhMYB25*, *GhHD-1*, and *GhHOX3*, in the regulatory network. We also identified genes such as *GhCER1* and *GhMYB60*, which appeared in the bulk RNA-seq data. These may serve as important candidate genes for the study of fiber initiation and development.

Genetic model for *GhMML3* regulation of fiber cell initiation

On the basis of the phenotypes of *GhMML3* mutants (single and double) and the results of bulk and single-cell RNA-seq, we propose a model for the initiation and development of fiber cells in which *GhMML3* acts as a hub gene (Figure 5J–5L). Precursor fiber cells begin to continuously protrude and then start their preliminary elongation during the period from −1 to 5 DPA. Expression of *GhMML3* increases at −1 to 0 DPA and 3 to 5 DPA, concurrent with the peaks of lint and fuzz fiber cell protrusion, respectively. When either *GhMML3_A12* or *GhMML3_D12* is knocked out, the overall expression of *GhMML3* decreases in the WT during these key periods, particularly at 3–5 DPA, falling below the minimum required for precursor fiber cell protrusion. Consequently, fiber cells are unable to protrude, leading to the fuzzless–linted fiber phenotype. When both *GhMML3_A12* and *GhMML3_D12* are knocked out, *GhMML3* expression significantly decreases throughout the entire −1 to 5 DPA period in the WT, preventing precursor fiber cells from protruding and resulting in a fiberless phenotype. *GhMYB25*, *GhHD-1*, and *GhVIN1* are crucial downstream genes of *GhMML3* and are required for fiber initiation. Silencing their functions reduces fiber cell protrusion between 3 and 5 DPA, as well as at 0 DPA. *GhHOX3* expression begins at 0 DPA and peaks at 1 DPA, reflecting its key role in the early post-protrusion elongation of fiber cells. *GhRDL1*, *GhEXPA*, and *GhEXPA2* act downstream of *GhHOX3*. Fiber cells that complete protrusion before 3 DPA are regulated by *GhHOX3* to undergo early elongation and become lint fibers, whereas those that protrude from 3 to 5 DPA are hindered by the reduced expression of *GhHOX3* from 5 to 20 DPA, causing them to become fuzz rather than lint fibers.

METHODS

Plant materials

As the standard genetic line of upland cotton (*G. hirsutum*), TM-1 has abundant fuzz fiber on the seed surface (Kohel et al., 1970). The

recessive fuzzless–linted mutant n_2NSM was obtained from the USDA–ARS in College Station, Texas, and has very little fuzz fiber on its seed surface (Ware et al., 1947). These two genotypes were crossed to produce an F_1 generation with n_2NSM as the female parent. Three F_2 mapping populations (1168, 2155, and 1029 individuals, respectively) were created through F_1 self-pollination. To establish the additional BC_1 mapping population (1005 individuals), F_1 plants were crossed with the recurrent parent n_2NSM. All materials and populations were developed at Jiangpu Experimental Station (Jiangsu, China), Dangtu (Anhui, China), and Hainan Base (Hainan, China). DNA was extracted from new leaves. The transgenic recipient J668 was obtained from Huazhong Agricultural University (Supplemental Table 20).

Map-based cloning of the n_2 gene

We generated large F_2 and BC_1 populations to fine-map the n_2 gene on the basis of previous results from our laboratory on the positioning of this gene (Song et al., 2010). The n_2 gene was mapped to a 75.36-kb region using 155 simple sequence repeat (SSR) and InDel primers. The coding sequences of the candidate genes in TM-1 and n_2NSM were amplified using the primers listed in Supplemental Table 20, and the PCR products were confirmed by sequencing.

Quantitative reverse transcription PCR

Total RNA was extracted from ovules using the liquid nitrogen grinding technique according to the instructions of the EASYspin Plus Plant RNA Rapid Extraction Kit (#RK16, Molfarming). Reverse transcription was performed with HiScript II QRT SuperMix (Vazyme) to synthesize cDNA. Transcript analysis was performed using ChamQ Universal SYBR qPCR Master Mix (Vazyme) on a fluorescence quantitative reverse transcription PCR instrument (iQ5 model, Bio-Rad, USA). Each experimental group comprised three biological replicates, each with three technical replicates, to ensure the robustness and reliability of the results. *GhUBI1* (EU604080) was used as the normalization control (Li et al., 2005). The gene-specific primer sequences are provided in Supplemental Table 20.

Scanning electron microscopy analysis

Ovule samples were fixed overnight in 2.5% (v/v) glutaraldehyde in phosphate buffer (pH 5.2) and then post-fixed with 1% (v/v) OsO_4 in phosphate buffer for 1 h. The specimens were then sequentially dehydrated in 30%, 50%, 70%, 80%, 90%, and 95% (v/v) ethanol solutions. The dehydrated samples were then dried using a Hitachi HCP-2. To observe the initiation of fuzz fiber cells in 3–5-DPA ovules, adhesive tape was used to separate the lint fibers from the surface of the ovules. The sample surfaces were coated with silver powder using an E-1010/E-1020 ion sputter (Japan), and the samples were observed at 3.0 kV using a GeminiSEM 300 scanning electron microscope (Germany).

Creation and verification of transgenic plants

To create the CRISPR-Cas9 vector, we designed four pairs of sgRNAs to target the exons of *GhMML3* (Liu et al., 2017). The tRNA–gRNA complex was constructed by PCR amplification and ligated into the pRGEB32-GhU6.7-NPT II plasmid (Wang et al., 2018). To generate overexpression cotton plants, the full-length *GhMML3_D12* gene was cloned into the pWMV062 vector (WIMI, Jiangsu) under the control of the *CaMV35S* promoter. Transgenic cotton plants were produced by transforming the vector into *Agrobacterium* strain GV3101 and then infecting the hypocotyls of J668 (for gene editing) and n_2NSM (for overexpression). DNA was extracted from fresh leaves of the transgenic plants, and specific primers were used to confirm the presence of the transgenes in the overexpression and gene-edited plants. The primers listed in Supplemental Table 20 were used to amplify the sequence segment containing the sgRNA target site for high-throughput sequencing (HI-TOM) sequencing (Liu et al., 2019). The sequencing data were analyzed to determine the precise editing outcomes (Jin et al., 2006; Li et al., 2019).

RNA transcriptome sequencing and data analysis

Cotton bolls were collected from the transgenic materials (*#mml3-D12*, *#mml3-A12*, and *#mml3s*), J668, n_2NSM, and TM-1 at −3, −1, 0, 1, 3, and 5 DPA. The ovules were dissected from the cotton bolls, immediately frozen in liquid nitrogen, and stored at −70°C. We collected three biological replicates for each sample, for a total of 108 samples. Total RNA was extracted using the EASYspin Plus Plant RNA Rapid Extraction Kit (#RK16, Molfarming) and sequenced on the Illumina NovaSeq 6000 platform. The resulting clean reads were aligned to the TM-1 reference genome v.2.1 using HISAT2 (v.2.2.1) (Kim et al., 2015; Chen et al., 2018; Hu et al., 2019). featureCounts software (v.2.0.2) was used to build the count matrix (Liao et al., 2014), and fragments per kilobase of transcript per million mapped reads were then calculated using StringTie (v.2.1.4) with the TM-1 (v.2.1) genome annotation file (Pertea et al., 2015). Principal-component analysis and Pearson correlations were plotted using the R (v.4.2.3) correlation function (Pearson, 1901, 1909). Differential gene expression was analyzed using the R package DESeq2 (v.1.38.3) (Love et al., 2014) with the criteria of $|\log_2(\text{FoldChange})| \geq 1$ and $p \leq 0.05$ after correction for false discovery rate. Gene expression heatmaps were created using the R package pheatmap (v.1.0.12), and GO enrichment analysis of DEGs was performed using the R package clusterProfiler (v.4.6.2) (Yu et al., 2012).

Protoplast preparation and scRNA-seq library construction

A fresh enzymatic solution was prepared, comprising 1.5% (w/v) cellulase R10, 0.75% (w/v) Macerozyme R-10, 1% (w/v) hemicellulase, 0.4 M mannitol, 20 mM KCl, 20 mM MES (pH 5.7), 10 mM $CaCl_2$, and 0.1% (w/v) BSA. Thin slices of 0-DPA ovules were immersed in this solution and incubated in the dark at 28°C on a constant-temperature shaker for 2 h. After digestion, the protoplasts were sequentially filtered through 75-μm and 40-μm cell sieves, then precipitated by centrifugation at 200 rcf for 2 min at room temperature. The resulting protoplast pellet was resuspended in DPBS containing 8% mannitol and 0.04% BSA. This resuspension was layered onto a solution containing 20% and 40% Percoll and centrifuged at 400 rcf for 20 min. The intermediate layer was collected, filtered through a 40-μm cell filter, and centrifuged at 200 rcf for 2 min to remove the supernatant. Cell viability was evaluated using a 0.002% (w/v) fluorescein diacetate (FDA) solution, and cell concentration was determined using a cell counter and a microscope. The final concentration was adjusted to 1200 cells/μl. scRNA-seq libraries were prepared using 10× Genomics microfluidic technology and sequenced using the HiSeq PE150 strategy on an Illumina NovaSeq 6000 sequencer.

scRNA-seq data preprocessing

Raw sequencing data were exported in FASTQ format and demultiplexed using CellRanger software. The standard cotton reference genome TM-1 v.2.1 (ZJU) was used as the reference for comparison (Hu et al., 2019). The R package Seurat (v.4.0.1) was used to process the unique molecular identifiers (UMI) matrix and perform dimensionality reduction, clustering, and analysis of the data. After removal of genes expressed in fewer than three cells, the number of expressed genes per cell ranged from 400 to 5000, UMI counts were <150, and mtDNA-derived gene expression comprised <10%. DoubletFinder (v.2.0.3) was used to detect doublets in each scRNA-seq dataset, with principal components = 1:20, the maximum pK value taken as the optimal pK parameter, and the doublet formation rate assumed to be 7.5%. Gene expression values were calculated using the LogNormalize method of the "Normalization" function in Seurat. We normalized cell-cycle genes, mitochondrial genes, and protoplast-enzyme-induction genes using the "vars.to.regress" function of ScaleData. Principal-component analysis was performed using the normalized expression values. Marker genes for each cluster were identified using the FindAllMarkers function in Seurat. Genes expressed in more than 25% of the cells in a cluster and with an average $\log_2$(FoldChange) greater than 0.25 were selected as markers. The differential GeneTest function in the Monocle 2 v2.22.0 package (Qiu et al., 2017) was used to construct single-cell developmental trajectories, and the plot_cell_trajectory function

was used for visualization. Developmental trajectory reconstruction was performed using the R package SCORPIUS (v.1.0.8) (Cannoodt et al., 2016). The gene-expression heatmap of cells in different states along the developmental trajectory was visualized using the plot-genes-branched-heatmap function. WGCNA was performed following the official procedure of the WGCNA R package (1.70-3), using default parameters to construct a signed network (Langfelder and Horvath, 2008). Genes with consistent expression profiles were grouped into modules using average linkage hierarchical clustering, with topological overlap as the distance metric. Module gene centrality was defined as the sum of the intramodular connectivity measures and was used to rank genes within each module to determine their centrality. Finally, the gene regulatory network was visualized using Cytoscape.

EMSA

cis-elements in the promoters of *GhHD-1* and *GhMYB25* were predicted using the PlantRegMap website, and biotin-labeled probes ranging from 40 to 50 bp were designed accordingly (Supplemental Table 20). EMSA was performed using the EMSA/Gel-Shift Kit (GS009, Beyotime, China) according to the manufacturer's instructions. The GhMML3_D12-HeLa fusion protein was incubated with biotin-labeled probes in binding buffer at room temperature for 30 min. The reaction was then subjected to electrophoresis for 1 h on a 6% native polyacrylamide gel. DNA was transferred onto a nylon membrane and cross-linked using a UV-light cross-linker for 60 s. The signal was detected using BeyoECL Moon A and B liquids (5 ml each) for chemiluminescent detection.

Dual-LUC transient expression assay

The coding sequence of *GhMML3_D12* was cloned into the pGreen II-62-SK vector as an effector construct, and the *proGhHD-1*::LUC, *proGhMYB25*::LUC, and pGreen II-0800-LUC vectors were used as reporter constructs. These reporter and effector constructs were introduced into *Agrobacterium* strain GV3101. The *Agrobacteria* were resuspended in 10 mM $MgCl_2$, 10 mM MES (pH 5.6), and 20 μM acetosyringone and then used to infiltrate tobacco leaves. The plants were incubated for 72 h, followed by fluorescence imaging after a 10-min dark treatment with luciferin.

The activities of LUC (firefly luciferase) and REN (*Renilla* luciferase) in the leaves were quantified using the Dual-Luciferase Reporter Assay System (E1910, Promega, USA).

Accession numbers

The gene sequence data used in this article can be found in the Cotton Functional Genomics Database (http://cotton.zju.edu.cn) under the accession numbers in Supplemental Table 20.

DATA AND CODE AVAILABILITY

All sequencing data generated in this study have been deposited in the NCBI SRA database under BioProject IDs PRJNA1138945 and PRJNA869296.

FUNDING

This study was financially supported by grants from STI 2030-Major Projects (2023ZD0403802), the Fundamental Research Funds for the Central Universities (226-2022-00100), the NSFC (32130075), the Xinjiang Production and Construction Corps (2023AA008), and Research Startup Funding from Hainan Institute of Zhejiang University (0202-6602-A12301).

ACKNOWLEDGMENTS

We thank the Agricultural Experiment Station at Zhejiang University for their support in the greenhouse material planting and management. We also extend our gratitude to the Bio-ultrastructure Analysis Lab of the Analysis Center of Agrobiology and Environmental Sciences at Zhejiang University for their support with scanning electron microscopy and transmission electron microscopy. No conflict of interest is declared.

AUTHOR CONTRIBUTIONS

T.Z. conceived and designed the project. R.C., J.Z., and Y.H. performed the research. R.C., J.L., and J.C. prepared and analyzed the RNA-seq data. J.Z. provided the scRNA-seq data. R.C., J.Z., and F.D. analyzed the scRNA-seq data. T.Z., R.C., J.Z., Y.T., and Q.-H.Z. participated in writing and revising the manuscript.

SUPPLEMENTAL INFORMATION

Supplemental information is available at *Plant Communications Online*.

Received: August 22, 2024
Revised: January 1, 2025
Accepted: February 10, 2025
Published: February 12, 2025

REFERENCES

Bao, Y., Hu, G., Grover, C.E., Conover, J., Yuan, D., and Wendel, J.F. (2019). Unraveling cis and trans regulatory evolution during cotton domestication. Nat. Commun. **10**:5399.

Beasley, C.A., and Ting, I.P. (1973). The effects of plant growth substances on in vitro fiber development from fertilized cotton ovules. Am. J. Bot. **60**:130–139.

Bechere, E., Turley, R.B., Auld, D.L., and Zeng, L. (2012). A new fuzzless seed locus in an upland cotton (*Gossypium hirsutum* L.) mutant. Am. J. Plant Sci. **3**:799–804.

Birchler, J.A., and Yang, H. (2022). The multiple fates of gene duplications: deletion, hypofunctionalization, subfunctionalization, neofunctionalization, dosage balance constraints, and neutral variation. Plant Cell **34**:2466–2474.

Cannoodt, R., Saelens, W., Sichien, D., Tavernier, S., Janssens, S., Guilliams, M., Lambrecht, B., Preter, K.D., and Saeys, Y. (2016). SCORPIUS improves trajectory inference and identifies novel modules in dendritic cell development. Preprint at bioRxiv. https://doi.org/10.1101/079509.

Chen, S., Zhou, Y., Chen, Y., and Gu, J. (2018). fastp: an ultra-fast all-in-one FASTQ preprocessor. Bioinformatics **34**:884–890.

Chen, W., Li, Y., Zhu, S., Fang, S., Zhao, L., Guo, Y., Wang, J., Yuan, L., Lu, Y., Liu, F., et al. (2020a). A retrotransposon insertion in GhMML3_D12 is likely responsible for the lintless locus li3 of tetraploid cotton. Front. Plant Sci. **11**:593679.

Chen, Z.J., Sreedasyam, A., Ando, A., Song, Q., De Santiago, L.M., Hulse-Kemp, A.M., Ding, M., Ye, W., Kirkbride, R.C., Jenkins, J., et al. (2020b). Genomic diversifications of five *Gossypium allopolyploid* species and their impact on cotton improvement. Nat. Genet. **52**:525–533.

Deng, F., Tu, L., Tan, J., Li, Y., Nie, Y., and Zhang, X. (2012). *GbPDF1* is involved in cotton fiber initiation via the core cis-element HDZIP2ATATHB2. Plant Physiol. **158**:890–904.

Ding, M., Cao, Y., He, S., Sun, J., Dai, H., Zhang, H., Sun, C., Jiang, Y., Paterson, A.H., and Rong, J. (2020). GaHD1, a candidate gene for the Gossypium arboreum SMA-4 mutant, promotes trichome and fiber initiation by cellular H 2 O 2 and Ca 2+ signals. Plant Mol. Biol. **103**:409–423.

Du, C., Sun, W., Song, Q., and Zuo, K. (2024). GhDOFD45 promotes sucrose accumulation in cotton seeds by transcriptionally activating *GhSWEET10* expression. Plant J. **120**:2468–2484.

Endrizzi, J., Turcotte, E., and Kohel, R. (1985). Genetics, cytology, and evolution of *Gossypium*. Adv. Genet. **23**:271–375.

Flagel, L.E., Wendel, J.F., and Udall, J.A. (2012). Duplicate gene evolution, homoeologous recombination, and transcriptome characterization in allopolyploid cotton. BMC Genom. **13**:1–13.

Grover, C.E., Gallagher, J.P., Szadkowski, E.P., Yoo, M.J., Flagel, L.E., and Wendel, J.F. (2012). Homoeolog expression bias and expression level dominance in allopolyploids. New Phytol. **196**:966–971.

Guan, X.Y., Li, Q.J., Shan, C.M., Wang, S., Mao, Y.B., Wang, L.J., and Chen, X.Y. (2008). The HD-Zip IV gene *GaHOX1* from cotton is a functional homologue of the *Arabidopsis GLABRA2*. Physiol. Plantarum **134**:174–182.

Haigler, C.H., Betancur, L., Stiff, M.R., and Tuttle, J.R. (2012). Cotton fiber: a powerful single-cell model for cell wall and cellulose research. Front. Plant Sci. **3**:104.

Hu, Y., Chen, J., Fang, L., Zhang, Z., Ma, W., Niu, Y., Ju, L., Deng, J., Zhao, T., Lian, J., et al. (2019). *Gossypium barbadense* and *Gossypium hirsutum* genomes provide insights into the origin and evolution of allotetraploid cotton. Nat. Genet. **51**:739–748.

Huang, G., Huang, J.Q., Chen, X.Y., and Zhu, Y.X. (2021). Recent advances and future perspectives in cotton research. Annu. Rev. Plant Biol. **72**:437–462.

Huang, G., Wu, Z., Percy, R.G., Bai, M., Li, Y., Frelichowski, J.E., Hu, J., Wang, K., Yu, J.Z., and Zhu, Y. (2020). Genome sequence of *Gossypium herbaceum* and genome updates of *Gossypium arboreum* and *Gossypium hirsutum* provide insights into cotton A-genome evolution. Nat. Genet. **52**:516–524.

Jin, S., Zhang, X., Nie, Y., Guo, X., Liang, S., and Zhu, H. (2006). Identification of a novel elite genotype for in vitro culture and genetic transformation of cotton. Biol. Plant. (Prague) **50**:519–524.

Kearney, T.H., and Harrison, R.J. (1927). Inheritance of smooth seed in cotton. J. Agric. Res. **35**:193.

Kim, D., Langmead, B., and Salzberg, S.L. (2015). HISAT: a fast spliced aligner with low memory requirements. Nat. Methods **12**:357–360.

Kohel, R.J. (1973a). Analysis of irradiation induced virescent mutants and the identification of a new virescent mutant (v_5v_5, v_6v_6) in *Gossypium hirsutum* L. Crop Sci. **13**:86–88.

Kohel, R.J. (1973b). Genetic nomenclature in cotton. J. Hered. **64**:291–295.

Kohel, R.J. (1983). Genetic analysis of virescent mutants and the identification of virescents v_{12}, v_{13}, v_{14}, v_{15} and $v_{16}v_{17}$ in Upland cotton. Crop Sci. **23**:289–291.

Kohel, R.J., Richmond, T.R., and Lewis, C.F. (1970). Texas Marker-1. Description of a Genetic Standard for Gossypium hirsutum L. 1. Crop Sci. **10**:670–671.

Langfelder, P., and Horvath, S. (2008). WGCNA: an R package for weighted correlation network analysis. BMC Bioinf. **9**:559.

Li, J., Wang, M., Li, Y., Zhang, Q., Lindsey, K., Daniell, H., Jin, S., and Zhang, X. (2019). Multi-omics analyses reveal epigenomics basis for cotton somatic embryogenesis through successive regeneration acclimation process. Plant Biotechnol. J. **17**:435–450.

Li, X.B., Fan, X.P., Wang, X.L., Cai, L., and Yang, W.C. (2005). The cotton *ACTIN1* gene is functionally expressed in fibers and participates in fiber elongation. Plant Cell **17**:859–875.

Liao, W.B., Ruan, M.b., Cui, B.m., Xu, N.f., Lu, J.j., and Peng, M. (2009). Isolation and characterization of a *GAI/RGA-like* gene from *Gossypium hirsutum*. Plant Growth Regul. **58**:35–45.

Liao, Y., Smyth, G.K., and Shi, W. (2014). featureCounts: an efficient general purpose program for assigning sequence reads to genomic features. Bioinformatics **30**:923–930.

Liu, H., Ding, Y., Zhou, Y., Jin, W., Xie, K., and Chen, L.L. (2017). CRISPR-P 2.0: an improved CRISPR-Cas9 tool for genome editing in plants. Mol. Plant **10**:530–532.

Liu, Q., Wang, C., Jiao, X., Zhang, H., Song, L., Li, Y., Gao, C., and Wang, K. (2019). Hi-TOM: a platform for high-throughput tracking of mutations induced by CRISPR/Cas systems. Sci. China Life Sci. **62**:1–7.

Love, M.I., Huber, W., and Anders, S. (2014). Moderated estimation of fold change and dispersion for RNA-seq data with DESeq2. Genome Biol. **15**:1–21.

Ma, D., Hu, Y., Yang, C., Liu, B., Fang, L., Wan, Q., Liang, W., Mei, G., Wang, L., Wang, H., et al. (2016). Genetic basis for glandular trichome formation in cotton. Nat. Commun. **7**:10456.

Ma, Z., Zhang, Y., Wu, L., Zhang, G., Sun, Z., Li, Z., Jiang, Y., Ke, H., Chen, B., Liu, Z., et al. (2021). High-quality genome assembly and resequencing of modern cotton cultivars provide resources for crop improvement. Nat. Genet. **53**:1385–1391.

Machado, A., Wu, Y., Yang, Y., Llewellyn, D.J., and Dennis, E.S. (2009). The MYB transcription factor *GhMYB25* regulates early fibre and trichome development. Plant J. **59**:52–62.

Mao, Y., Dai, F., Si, Z., Fang, L., and Zhang, T. (2023). Duplicate mutations of GhCYP450 lead to the production of ms5m6 male sterile line in cotton. Theor. Appl. Genet. **136**:2.

Pattanaik, S., Patra, B., Singh, S.K., and Yuan, L. (2014). An overview of the gene regulatory network controlling trichome development in the model plant, *Arabidopsis*. Front. Plant Sci. **5**:259.

Pearson, K. (1901). On lines and planes of closest fit to systems of points in space. Philos. Mag. **2**:559–572.

Pearson, K. (1909). Determination of the coefficient of correlation. Science **30**:23–25.

Pei, Y., Zhang, J., Wu, P., Ye, L., Yang, D., Chen, J., Li, J., Hu, Y., Zhu, X., Guo, X., and Zhang, T. (2021). *GoNe* encoding a class VIIIb *AP2/ERF* is required for both extrafloral and floral nectary development in *Gossypium*. Plant J. **106**:1116–1127.

Pertea, M., Pertea, G.M., Antonescu, C.M., Chang, T.C., Mendell, J.T., and Salzberg, S.L. (2015). StringTie enables improved reconstruction of a transcriptome from RNA-seq reads. Nat. Biotechnol. **33**:290–295.

Qian, N., Zhang, X.W., Guo, W.Z., and Zhang, T.Z. (2009). Fine mapping of open-bud duplicate genes in homoelogous chromosomes of tetraploid cotton. Euphytica **165**:325–331.

Qin, Y., Sun, M., Li, W., Xu, M., Shao, L., Liu, Y., Zhao, G., Liu, Z., Xu, Z., You, J., et al. (2022). Single-cell RNA-seq reveals fate determination control of an individual fibre cell initiation in cotton (*Gossypium hirsutum*). Plant Biotechnol. J. **20**:2372–2388.

Qin, Y.M., Hu, C.Y., Pang, Y., Kastaniotis, A.J., Hiltunen, J.K., and Zhu, Y.X. (2007). Saturated very-long-chain fatty acids promote cotton fiber and *Arabidopsis* cell elongation by activating ethylene biosynthesis. Plant Cell **19**:3692–3704.

Qiu, X., Mao, Q., Tang, Y., Wang, L., Chawla, R., Pliner, H.A., and Trapnell, C. (2017). Reversed graph embedding resolves complex single-cell trajectories. Nat. Methods **14**:979–982.

Rerie, W.G., Feldmann, K.A., and Marks, M.D. (1994). The *GLABRA2* gene encodes a homeo domain protein required for normal trichome development in *Arabidopsis*. Genes Dev. **8**:1388–1399.

Rong, J., Pierce, G.J., Waghmare, V.N., Rogers, C.J., Desai, A., Chee, P.W., May, O.L., Gannaway, J.R., Wendel, J.F., Wilkins, T.A., et al. (2005). Genetic mapping and comparative analysis of seven mutants related to seed fiber development in cotton. Theor. Appl. Genet. **111**:1137–1146.

Serna, L., and Martin, C. (2006). Trichomes: different regulatory networks lead to convergent structures. Trends Plant Sci. **11**:274–280.

Shan, C.M., Shangguan, X.X., Zhao, B., Zhang, X.F., Chao, L.M., Yang, C.Q., Wang, L.J., Zhu, H.Y., Zeng, Y.D., Guo, W.Z., et al. (2014). Control of cotton fibre elongation by a homeodomain transcription factor *GhHOX3*. Nat. Commun. **5**:5519.

Simin, L., Dongyun, Z., Hailiang, C., Youping, Z., Qiaolian, W., Ke, L., Xiaoxu, F., Hongwei, G., and Guoli, S. (2019). Fine mapping of the fuzzless gene n_2 in cotton. Cotton Sci **31**:114–120.

Song, L., Guo, W., Qin, H., Ding, Y., and Zhang, T. (2010). Genetic analysis and molecular validation of chromosome assignment for fuzzless genes N_1 and n_2 in cotton. J. Nanjing Agric. Univ. **33**:21–26.

Stewart, J.M. (1975). Fiber initiation on the cotton ovule (*Gossypium hirsutum*). Am. J. Bot. **62**:723–730.

Stracke, R., Werber, M., and Weisshaar, B. (2001). The *R2R3-MYB* gene family in *Arabidopsis thaliana*. Curr. Opin. Plant Biol. **4**:447–456.

Sun, Y., Yuan, Y., He, S., Stiller, W., Wilson, I., Du, X., and Zhu, Q.H. (2024). Dissecting the major genetic components underlying cotton lint development. Genetics **226**:iyad219.

Tian, Y., and Zhang, T. (2021). MIXTAs and phytohormones orchestrate cotton fiber development. Curr. Opin. Plant Biol. **59**:101975.

Turley, R.B., and Kloth, R.H. (2002). Identification of a third fuzzless seed locus in upland cotton (*Gossypium hirsutum* L.). J. Hered. **93**:359–364.

Turley, R.B., and Kloth, R.H. (2008). The inheritance model for the fiberless trait in upland cotton (*Gossypium hirsutum* L.) line SL1-7-1: variation on a theme. Euphytica **164**:123–132.

Walford, S.A., Wu, Y., Llewellyn, D.J., and Dennis, E.S. (2011). *GhMYB25-like*: a key factor in early cotton fibre development. Plant J. **65**:785–797.

Walford, S.A., Wu, Y., Llewellyn, D.J., and Dennis, E.S. (2012). Epidermal cell differentiation in cotton mediated by the homeodomain leucine zipper gene, *GhHD-1*. Plant J. **71**:464–478.

Wan, Q., Guan, X., Yang, N., Wu, H., Pan, M., Liu, B., Fang, L., Yang, S., Hu, Y., Ye, W., et al. (2016). Small interfering RNAs from bidirectional transcripts of *GhMML3A_12* regulate cotton fiber development. New Phytol. **210**:1298–1310.

Wang, L., Kartika, D., and Ruan, Y.L. (2021a). Looking into 'hair tonics' for cotton fiber initiation. New Phytol. **229**:1844–1851.

Wang, L., Cook, A., Patrick, J.W., Chen, X.Y., and Ruan, Y.L. (2014). Silencing the vacuolar invertase gene *GhVIN1* blocks cotton fiber initiation from the ovule epidermis, probably by suppressing a cohort of regulatory genes via sugar signaling. Plant J. **78**:686–696.

Wang, M., Tu, L., Yuan, D., Zhu, D., Shen, C., Li, J., Liu, F., Pei, L., Wang, P., Zhao, G., et al. (2019). Reference genome sequences of two cultivated allotetraploid cottons, *Gossypium hirsutum* and *Gossypium barbadense*. Nat. Genet. **51**:224–229.

Wang, P., Zhang, J., Sun, L., Ma, Y., Xu, J., Liang, S., Deng, J., Tan, J., Zhang, Q., Tu, L., et al. (2018). High efficient multisites genome editing in allotetraploid cotton (*Gossypium hirsutum*) using CRISPR/Cas9 system. Plant Biotechnol. J. **16**:137–150.

Wang, X., Shen, C., Meng, P., Tan, G., and Lv, L. (2021b). Analysis and review of trichomes in plants. BMC Plant Biol. **21**:70.

Ware, J., Benedict, L., and Rolfe, W. (1947). A recessive naked-seed character in upland cotton. J. Hered. **38**:313–320.

Wu, H., Tian, Y., Wan, Q., Fang, L., Guan, X., Chen, J., Hu, Y., Ye, W., Zhang, H., Guo, W., et al. (2018). Genetics and evolution of MIXTA genes regulating cotton lint fiber development. New Phytol. **217**:883–895.

Yang, Z., Ge, X., Yang, Z., Qin, W., Sun, G., Wang, Z., Li, Z., Liu, J., Wu, J., Wang, Y., et al. (2019). Extensive intraspecific gene order and gene structural variations in upland cotton cultivars. Nat. Commun. **10**:2989.

Yang, Z., Liu, Z., Ge, X., Lu, L., Qin, W., Qanmber, G., Liu, L., Wang, Z., and Li, F. (2023). Brassinosteroids regulate cotton fiber elongation by modulating very-long-chain fatty acid biosynthesis. Plant Cell **35**:2114–2131.

You, J., Liu, Z., Qi, Z., Ma, Y., Sun, M., Su, L., Niu, H., Peng, Y., Luo, X., Zhu, M., et al. (2023). Regulatory controls of duplicated gene expression during fiber development in allotetraploid cotton. Nat. Genet. **55**:1987–1997.

Yu, C.-Y., Sharma, O., Nguyen, P.H.T., Hartono, C.D., and Kanehara, K. (2021). A pair of DUF538 domain-containing proteins modulates plant growth and trichome development through the transcriptional regulation of *GLABRA1* in *Arabidopsis thaliana*. Plant J. **108**:992–1004.

Yu, G., Wang, L.G., Han, Y., and He, Q.Y. (2012). clusterProfiler: an R package for comparing biological themes among gene clusters. OMICS A J. Integr. Biol. **16**:284–287.

Zhang, F., Zuo, K., Zhang, J., Liu, X., Zhang, L., Sun, X., and Tang, K. (2010). An L1 box binding protein, GbML1, interacts with GbMYB25 to control cotton fibre development. J. Exp. Bot. **61**:3599–3613.

Zhang, J., Si, Z., Chen, R., Liu, W., Shi, Y., Shi, Z., Mei, H., Hu, Y., Fang, L., and Zhang, T. (2023). A new model system for cotton indoor genetic and genomic research. Sci. China Life Sci. **66**:1444–1446.

Zhang, M., Zheng, X., Song, S., Zeng, Q., Hou, L., Li, D., Zhao, J., Wei, Y., Li, X., Luo, M., et al. (2011). Spatiotemporal manipulation of auxin biosynthesis in cotton ovule epidermal cells enhances fiber yield and quality. Nat. Biotechnol. **29**:453–458.

Zhang, T., and Pan, J. (1991). Genetic analysis of a fuzzless-lintless mutant in upland cotton. Jiangsu J Agric Sci **3**:13–16.

Zhang, T., Hu, Y., Jiang, W., Fang, L., Guan, X., Chen, J., Zhang, J., Saski, C.A., Scheffler, B.E., Stelly, D.M., et al. (2015). Sequencing of allotetraploid cotton (*Gossypium hirsutum* L. acc. TM-1) provides a resource for fiber improvement. Nat. Biotechnol. **33**:531–537.

Zhao, G., Le, Y., Sun, M., Xu, J., Qin, Y., Men, S., Ye, Z., Tan, H., Hu, H., You, J., et al. (2024). A dominant negative mutation of *GhMYB25-like* alters cotton fiber initiation, reducing lint and fuzz. Plant Cell **36**:2759–2777.

Zhao, M., Morohashi, K., Hatlestad, G., Grotewold, E., and Lloyd, A. (2008). The TTG1-bHLH-MYB complex controls trichome cell fate and patterning through direct targeting of regulatory loci. Development **135**:1991–1999.

Zhu, Q.H., Stiller, W., Moncuquet, P., Gordon, S., Yuan, Y., Barnes, S., and Wilson, I. (2021). Genetic mapping and transcriptomic characterization of a new fuzzless-tufted cottonseed mutant. G3 (Bethesda). **11**:1–14.

Zhu, Q.H., Yuan, Y., Stiller, W., Jia, Y., Wang, P., Pan, Z., Du, X., Llewellyn, D., and Wilson, I. (2018). Genetic dissection of the fuzzless seed trait in *Gossypium barbadense*. J. Exp. Bot. **69**:997–1009.

Plant Communications, Volume *6*

Supplemental information

Two duplicated *GhMML3* genes coordinately control development of lint and fuzz fibers in cotton

Rui Chen, Jun Zhang, Jun Li, Jinwen Chen, Fan Dai, Yue Tian, Yan Hu, Qian-Hao Zhu, and Tianzhen Zhang

Supplemental information for

Duplicated *GhMML3s* coordinately control cotton lint and fuzz fiber development

Rui Chen[1,2#], Jun Zhang[1,3#], Jun Li[2], Jinwen Chen[1], Fan Dai[1], Yue Tian[4], Yan Hu[1,2], Qian-Hao Zhu[5], Tianzhen Zhang[1,2*]

1. Zhejiang Provincial Key Laboratory of Crop Genetic Resources, Institute of Crop Science, Plant Precision Breeding Academy, College of Agriculture and Biotechnology, Zhejiang University, Hangzhou, China
2. Hainan Institute of Zhejiang University, Sanya, Hainan, 572025, China
3. Institute of Horticulture, Zhejiang Academy of Agricultural Sciences, Hangzhou, Zhejiang, China.
4. College of Biotechnology, Jiangsu University of Science and Technology, Zhenjiang, China.
5. CSIRO Agriculture and Food, GPO Box 1700, Canberra 2601, Australia

[#] These authors contributed equally to this work.

[*]Correspondence and requests for materials should be addressed to Dr. Tianzhen Zhang (cotton@zju.edu.cn)

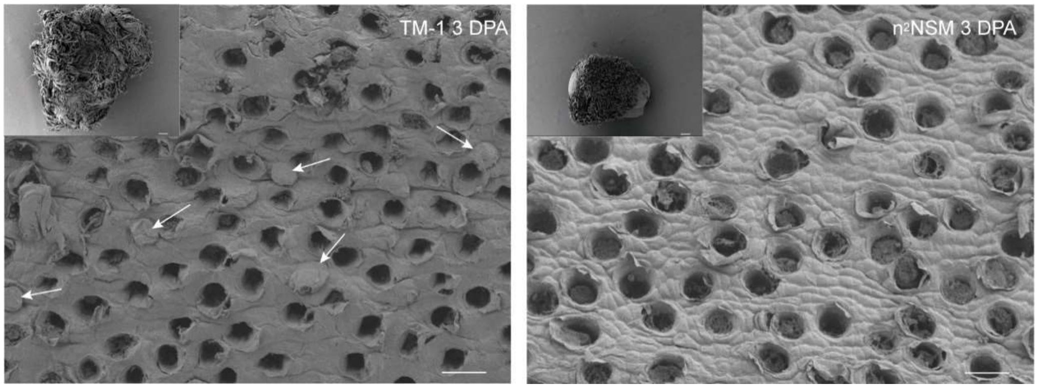

Supplemental Figure 1. Scanning electronic micrographs of TM-1 and n_2NSM at 3 DPA.

Arrows indicate fuzz cells. Scale bar = 20 μm.

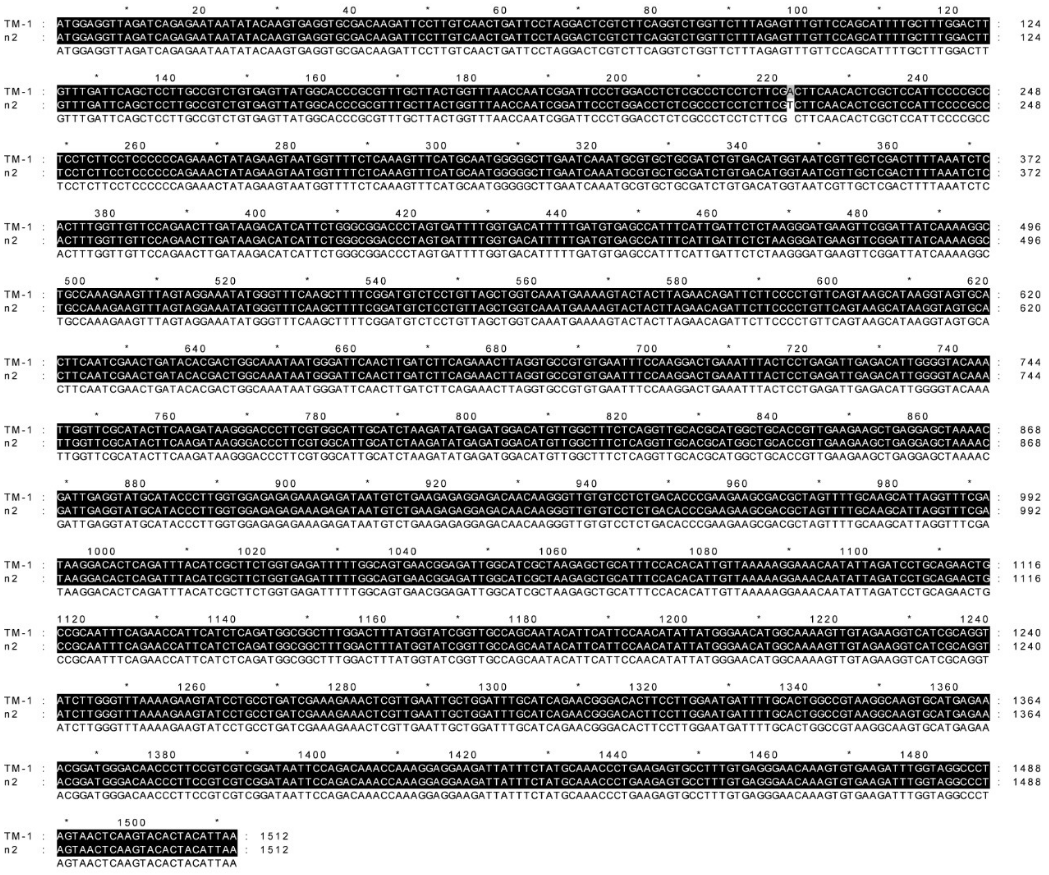

Supplemental Figure 2. Alignment of *ORF1* coding sequences from the accessions used in this study.

Black shading indicates coding sequences from n_2NSM and TM-1. On the gray backdrop, there is a single-base difference at 222 bp, but this difference has no effect on amino acid sequence.

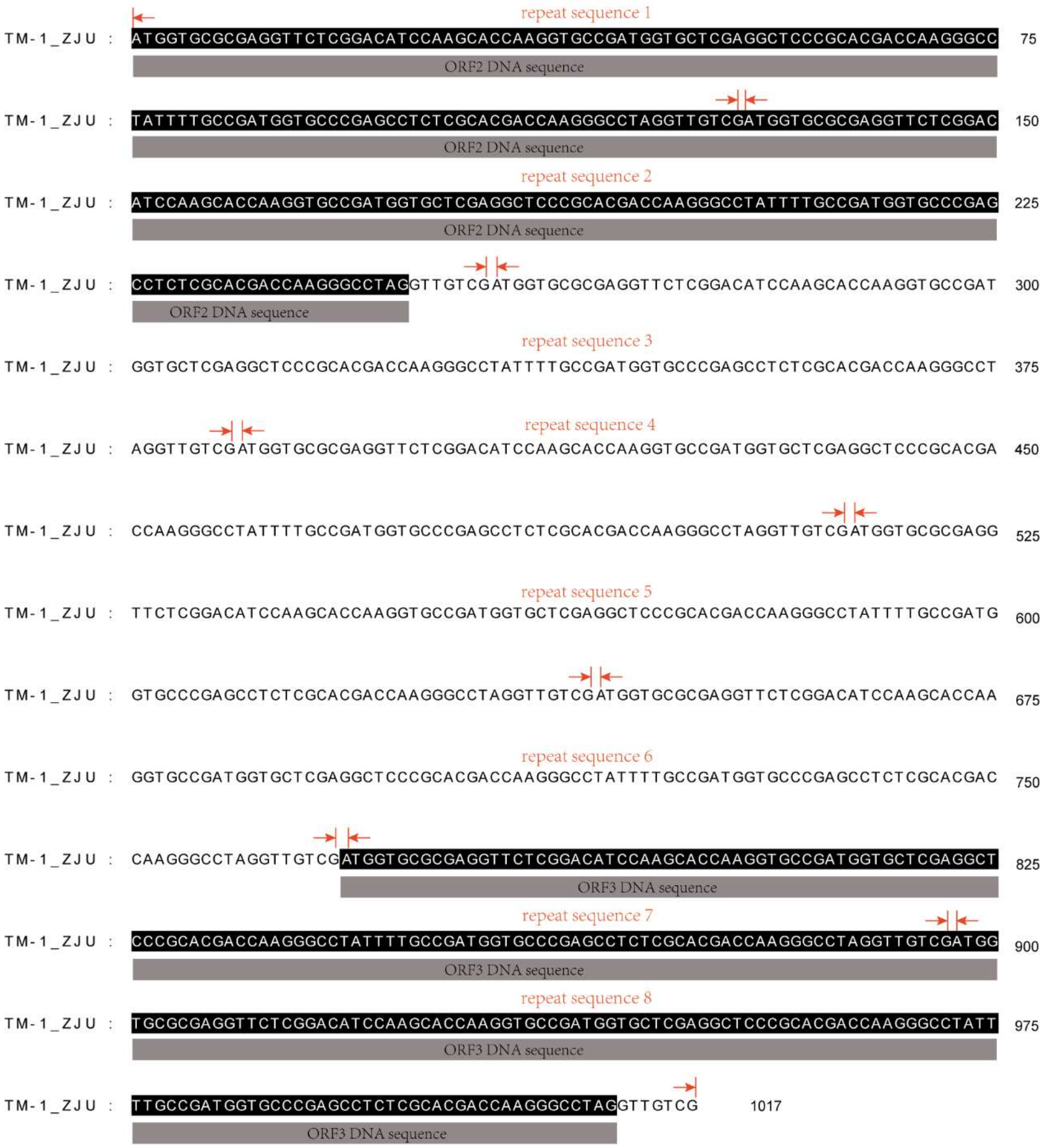

Supplemental Figure 3. The genome sequences of *ORF2* and *ORF3* from the accessions used in this study.

Black shading indicates DNA sequences from *Gossypium hirsutum* (ZJU assembly). The eight red rounded arrows in this region indicate the same repeated sequence (ATGGTG….TTGTCG).

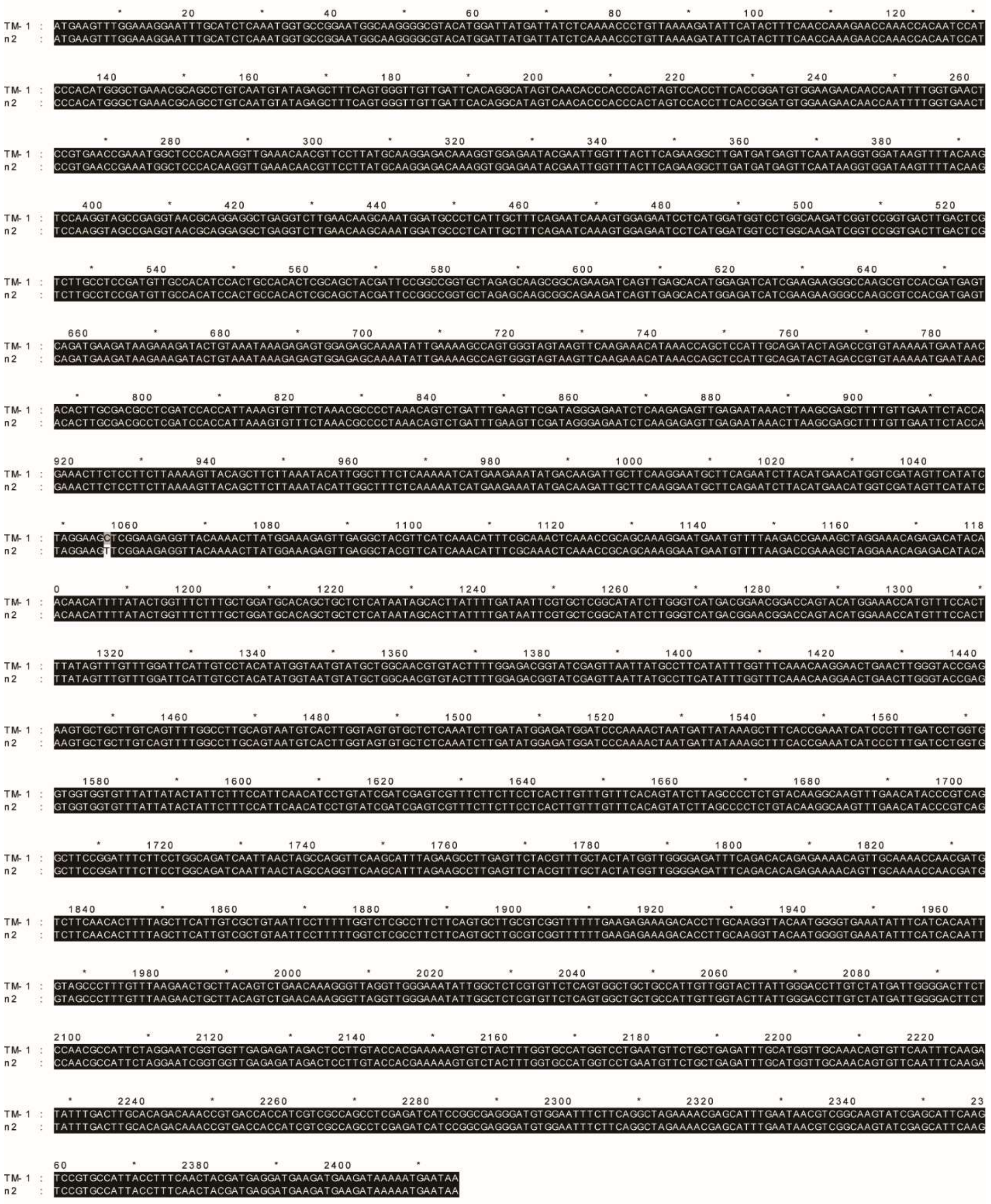

Supplemental Figure 4. Alignment of *ORF4* coding sequences from the accessions used in this study.

Black shading indicates coding sequences from TM-1 and n_2NSM. On the gray backdrop, there is a single-base difference at 1056 bp, but this difference has no effect on amino acid sequence.

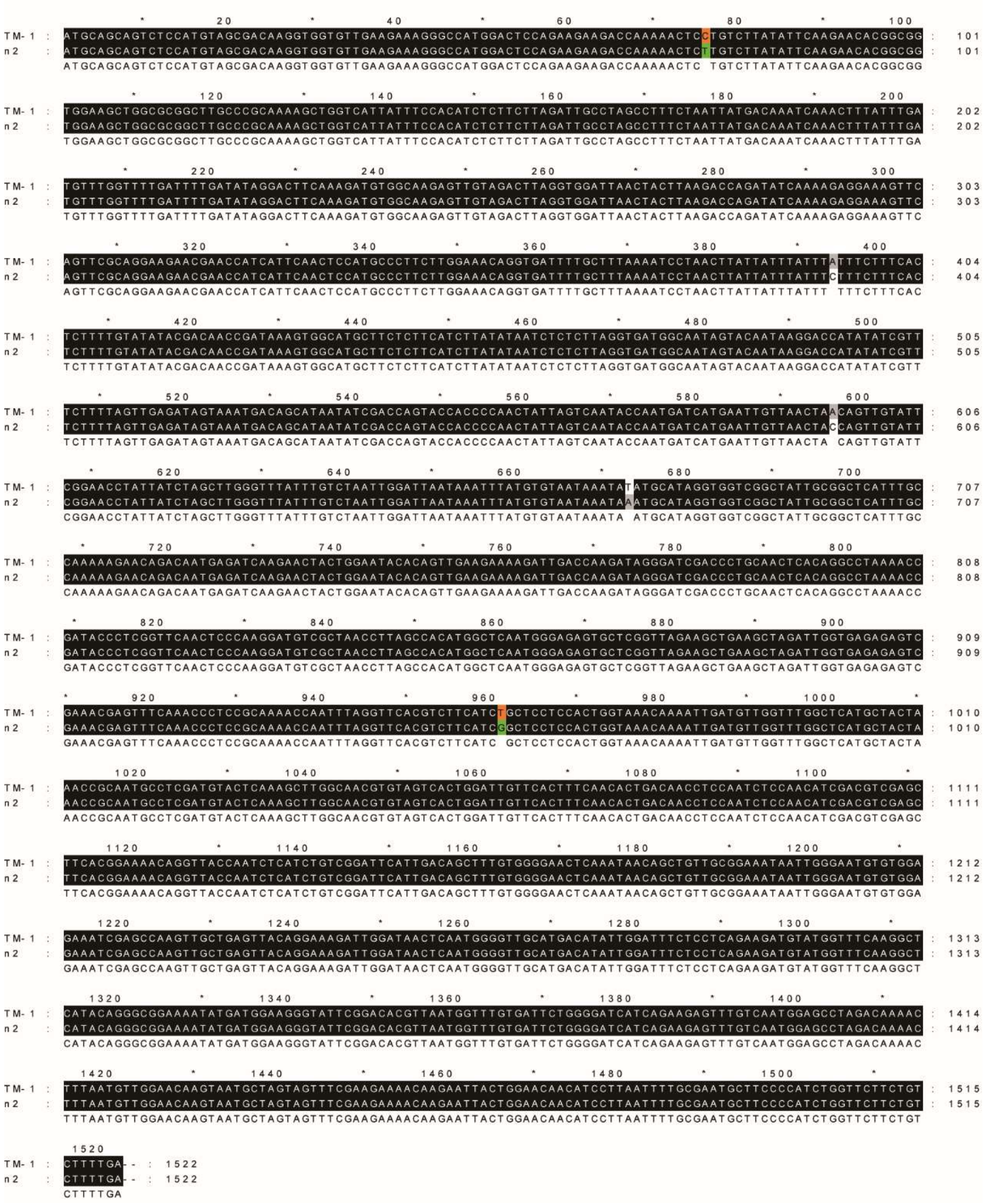

Supplemental Figure 5. Alignment of *GhMML3_D12* genome sequences from the accessions used in this study.

Black shading indicates coding sequences from TM-1 and n_2NSM. Orange and green backgrounds indicate SNP differences. There are two SNPs in the exons, at 76 bp and 961 bp respectively, but neither causes an amino acid difference.

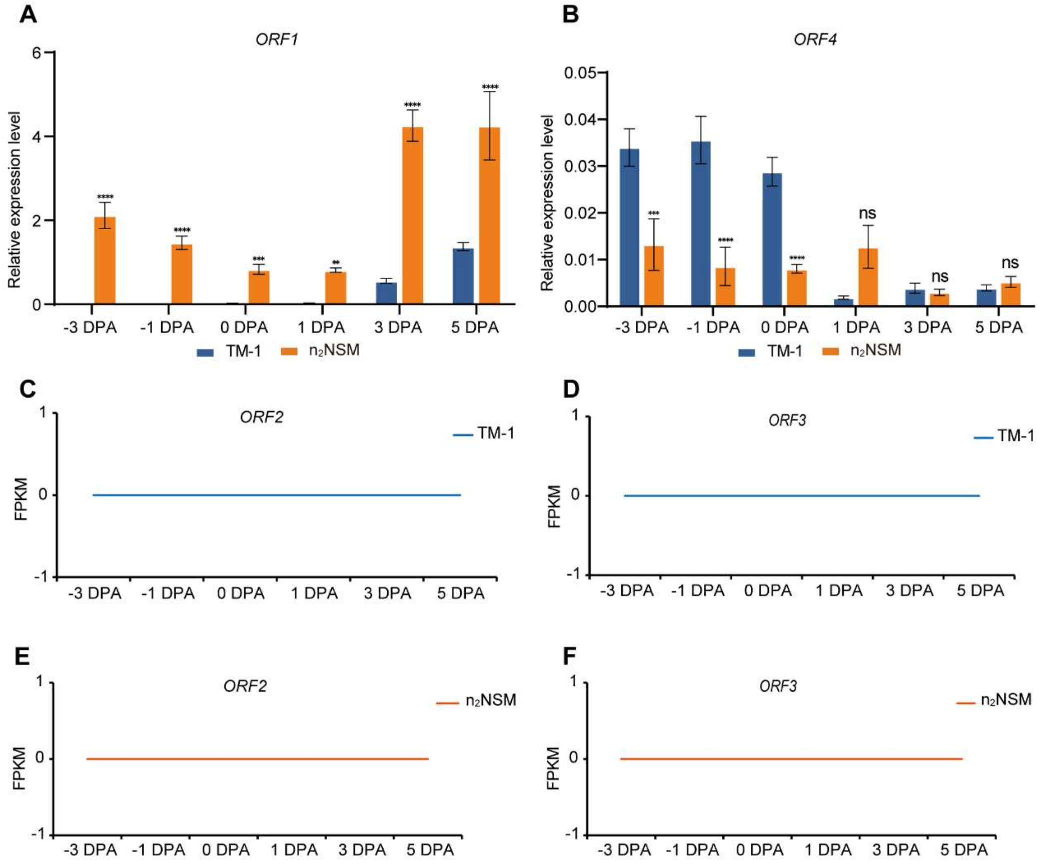

Supplemental Figure 6. Expression of *ORF1-4* in TM-1 and n_2NSM.
(A–B) Quantitative reverse transcription PCR of *ORF1* and *ORF4* in ovules of TM-1 and n_2NSM. Statistical significance was determined using one-way ANOVA. Data are represented as means ± SEM of three biological replicates. ****, $P < 0.0001$; ***, $P < 0.001$; **, $P < 0.01$. **(C–D)** Expression of *ORF2* and *ORF3* (as FPKM) in RNA-seq data from different developmental stages (including ovules at -3, -1, 0, 1, 3, 5 DPA) of TM-1 and n_2NSM.

```
                              sgRNA1
GhMM3_A12 : ATGCAGCAGTCTCCATGTAGCGACAAGGTGGGGTTGAAGAAAGGGCCATGGACTCCAGAAGAAGACCAAAAACTCTTGT :   79
GhMM3_D12 : ATGCAGCAGTCTCCATGTAGCGACAAGGTGGTGTTGAAGAAAGGGCCATGGACTCCAGAAGAAGACCAAAAACTCCTGT :   79

GhMM3_A12 : CTTATATTCAAGAACACGGCGGTGGAAGCTGGCGAGGCTTGCCCGCAAAAGCTGGACTTCAAAGATGTGGCAAGAGTTG :  158
GhMM3_D12 : CTTATATTCAAGAACACGGCGGTGGAAGCTGGCGCGGCTTGCCCGCAAAAGCTGGACTTCAAAGATGTGGCAAGAGTTG :  158

GhMM3_A12 : TAGACTTAGGTGGATTAACTACTTAAGACCAGATATCAAAAGAGGAAAGTTCAGTTCGCAGGAAGAACGAACCATCATT :  237
GhMM3_D12 : TAGACTTAGGTGGATTAACTACTTAAGACCAGATATCAAAAGAGGAAAGTTCAGTTCGCAGGAAGAACGAACCATCATT :  237

GhMM3_A12 : CAACTCCACGCCCTTCTTGGAAACAGGTGGTCGGCTATTGCGGCTCATTTGCCAAAAAGAACAGACAATGAGATCAAGA :  316
GhMM3_D12 : CAACTCCATGCCCTTCTTGGAAACAGGTGGTCGGCTATTGCGGCTCATTTGCCAAAAAGAACAGACAATGAGATCAAGA :  316

                                    sgRNA3
GhMM3_A12 : ACTACTGGAATACACAGTTGAAGAAAAGGTTGACGACGATAGGGATCGACCCTGCAACTCACAGGCCTAAAACCGATAC :  395
GhMM3_D12 : ACTACTGGAATACACAGTTGAAGAAAAGATTGACCAAGATAGGGATCGACCCTGCAACTCACAGGCCTAAAACCGATAC :  395

GhMM3_A12 : CCTCGGTTCAACTCCCAAGGATGCCGCTAACCTTAGCCACATGGCTCAATGGGAGAGTGCTCGGTTAGAAGCTGAAGCT :  474
GhMM3_D12 : CCTCGGTTCAACTCCCAAGGATGTCGCTAACCTTAGCCACATGGCTCAATGGGAGAGTGCTCGGTTAGAAGCTGAAGCT :  474

GhMM3_A12 : AGATTGGTGAGAGAGTCGAAACGAGTTTCAAACCCTCCGCAAAACCAATTTAGGTTCACGTCTTCATCGGCTCCTCCAC :  553
GhMM3_D12 : AGATTGGTGAGAGAGTCGAAACGAGTTTCAAACCCTCCGCAAAACCAATTTAGGTTCACGTCTTCATCGGCTCCTCCAC :  553

GhMM3_A12 : TGGTAAGCAAAATTGATGTTGGTTTGGCTCATGCTACTAAACCGCAATGCCTCGATGTACTCAAAGCTTGGCAACGTGT :  632
GhMM3_D12 : TGGTAAACAAAATTGATGTTGGTTTGGCTCATGCTACTAAACCGCAATGCCTCGATGTACTCAAAGCTTGGCAACGTGT :  632

                                                  sgRNA2
GhMM3_A12 : AGTCACTGGATTGTTCACTTTCAACACTGACAACCTCCAATCTCCAACATCGACGTCGAGCTTCACGGAAAACACGTTA :  711
GhMM3_D12 : AGTCACTGGATTGTTCACTTTCAACACTGACAACCTCCAATCTCCAACATCGACGTCGAGCTTCACGGAAAACAGGTTA :  711
                                                                      sgRNA4

GhMM3_A12 : CCAATCTCATCTGTCGGGTTCATTGACAGCTTTGTGGGGAACTCAAATAACAGCTGTTGCGGAAATAATTGGGAATGTG :  790
GhMM3_D12 : CCAATCTCATCTGTCGGATTCATTGACAGCTTTGTGGGGAACTCAAATAACAGCTGTTGCGGAAATAATTGGGAATGTG :  790

GhMM3_A12 : TGGAGAAATCGAGCCAAGTTGCTGAATTACAGGAAATATTGGATAACTCAATGGGGTTGCATGACATATTGGATCTCTC :  869
GhMM3_D12 : TGGAGAAATCGAGCCAAGTTGCTGAGTTACAGGAAAGATTGGATAACTCAATGGGGTTGCATGACATATTGGATTTCTC :  869

GhMM3_A12 : CTCAGAAGATGTATGGTTTCAAGGCTCATACAGGGCGGAAAATATGATGGAAGGGTATTCGGACACGTTAATGGTTTGT :  948
GhMM3_D12 : CTCAGAAGATGTATGGTTTCAAGGCTCATACAGGGCGGAAAATATGATGGAAGGGTATTCGGACACGTTAATGGTTTGT :  948

GhMM3_A12 : GATTCTGGGGATCATCCGAAGAGTTTGTCAATGGAGCCTAGACAAAACTTTAATGTTGGAACAAGTAATGCTAGTAGTT : 1027
GhMM3_D12 : GATTCTGGGGATCATCAGAAGAGTTTGTCAATGGAGCCTAGACAAAACTTTAATGTTGGAACAAGTAATGCTAGTAGTT : 1027

GhMM3_A12 : TCGAAGAAAACAAGAATTACTGGAACAACATCCTTAATTTTGCGAATGCTTCCCCTTCTGGTTCTTCTGTCTTTTGA : 1104
GhMM3_D12 : TCGAAGAAAACAAGAATTACTGGAACAACATCCTTAATTTTGCGAATGCTTCCCCATCTGGTTCTTCTGTCTTTTGA : 1104
```

Supplemental Figure 7. Location of sgRNAs in *GhMML3* genes.

sgRNA1 and sgRNA2 were used for simultaneous editing of *GhMML3_A12* and *GhMML3_D12* (#*mml3s*), while sgRNA3 targeted *GhMML3_A12* alone (#*mml3-A12*) and sgRNA4 *GhMML3_D12* alone (#*mml3-D12*).

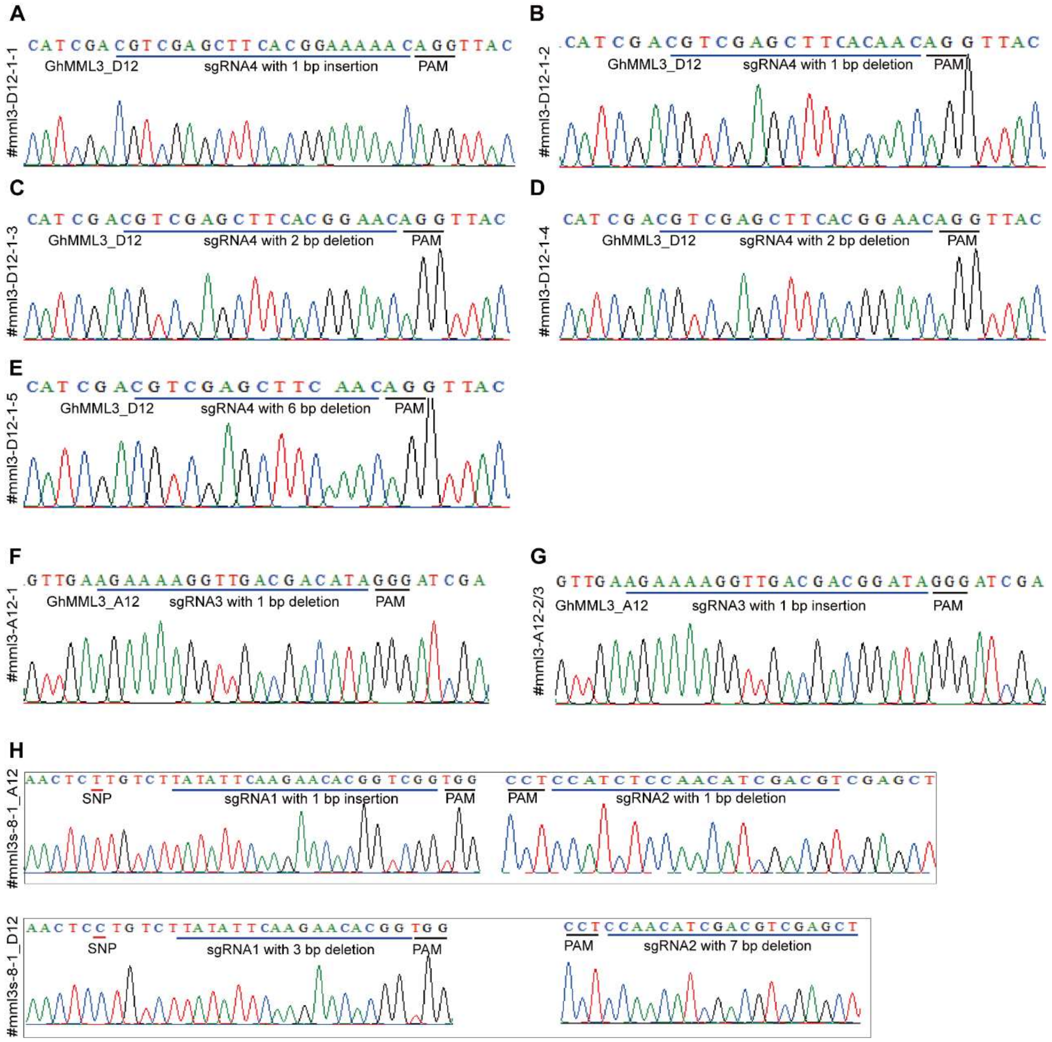

Supplemental Figure 8. Sequencing peak traces at sgRNA target sites in transgenic plants.

(A–E) Five sequencing traces showing *GhMML3_D12* gene editing status in *#mml3-D12* plants. **(F–G)** Two sequencing traces showing *GhMML3_A12* status in *#mml3-A12* plants. **(H)** Sequencing traces showing *GhMML3_A12* and *GhMML3_D12* status in *#mml3s-8-1* plants. PAM regions and sgRNAs sites are underlined with black and blue lines, respectively.

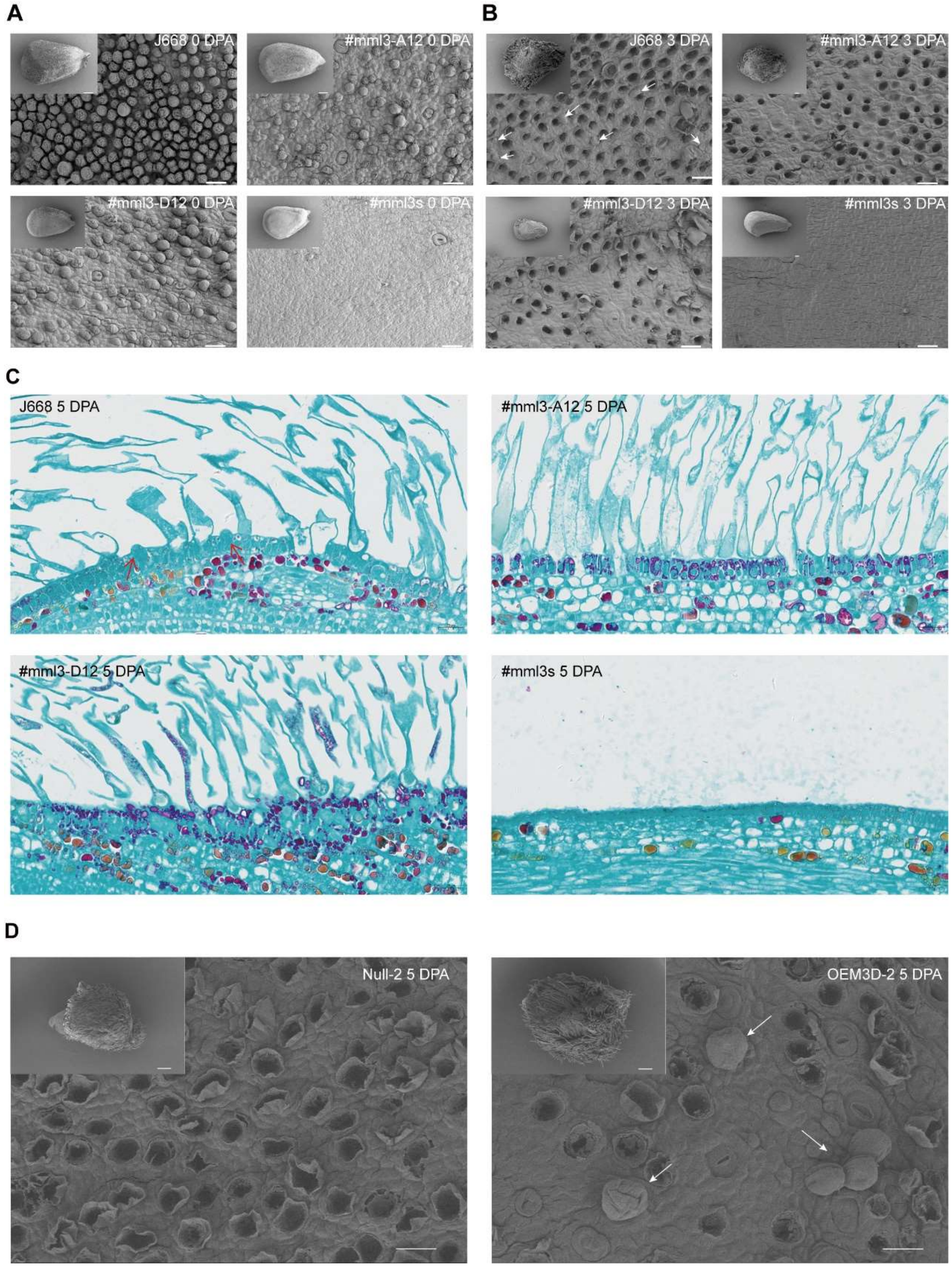

Supplemental Figure 9. Scanning electron micrographs and paraffin sections of *GhMML3* transgenic plants.

(A–B) Scanning electronic micrographs of *#mml3-A12*, *#mml3-D12*, and *#mml3-8-1* of ovules at 0 and 3 DPA. Arrows indicate initiating fuzz fiber cells. Scale bar = 20 µm (bottom), 200 µm (top). **(C)** paraffin sections of ovules of transgenic plants at 5 DPA. Red arrows indicate fuzz cells. Scale bar = 25 µm. **(D)** Scanning electronic micrographs of Null-2 and *OEM3D-2* seed epidermises at 5 DPA. Arrows indicate initiating fuzz fiber cells. Scale bar = 20 µm (bottom), 200 µm (top).

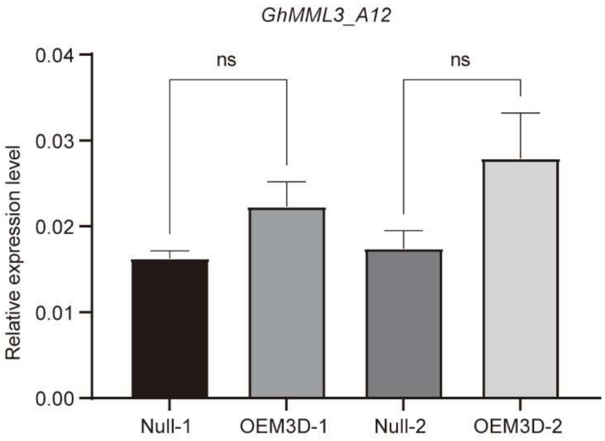

Supplemental Figure 10. Quantitative reverse transcription PCR of *GhMML3_A12* in plants over-expressing *GhMML3_D12*.

Statistical significance was determined using one-way ANOVA. Data are represented as means ± SEM of three biological replicates.

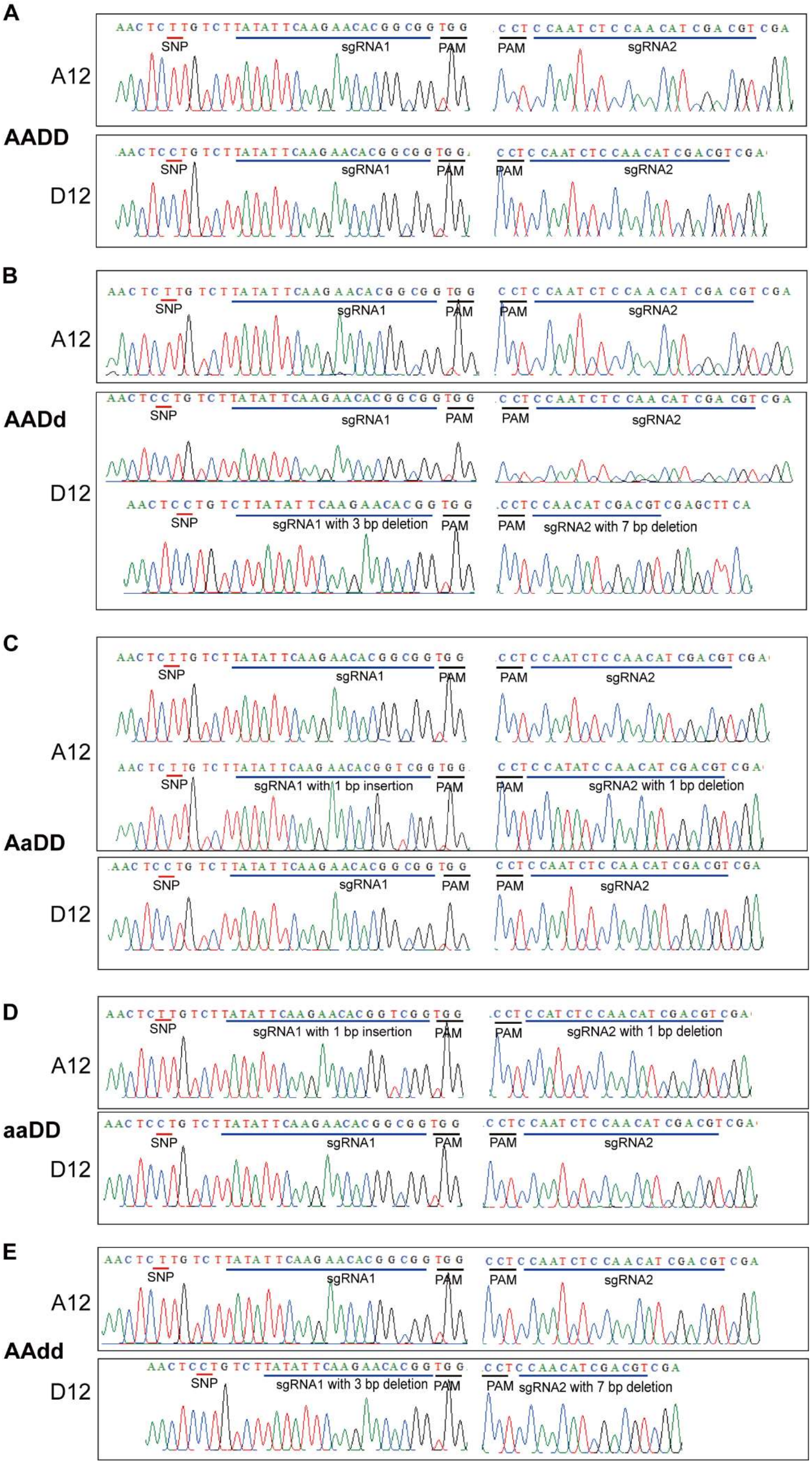

A
A12
AADD
D12
SNP
sgRNA1
PAM
sgRNA2
B
AADd
sgRNA1 with 3 bp deletion
sgRNA2 with 7 bp deletion
C
AaDD
sgRNA1 with 1 bp insertion
sgRNA2 with 1 bp deletion
D
aaDD
E
AAdd

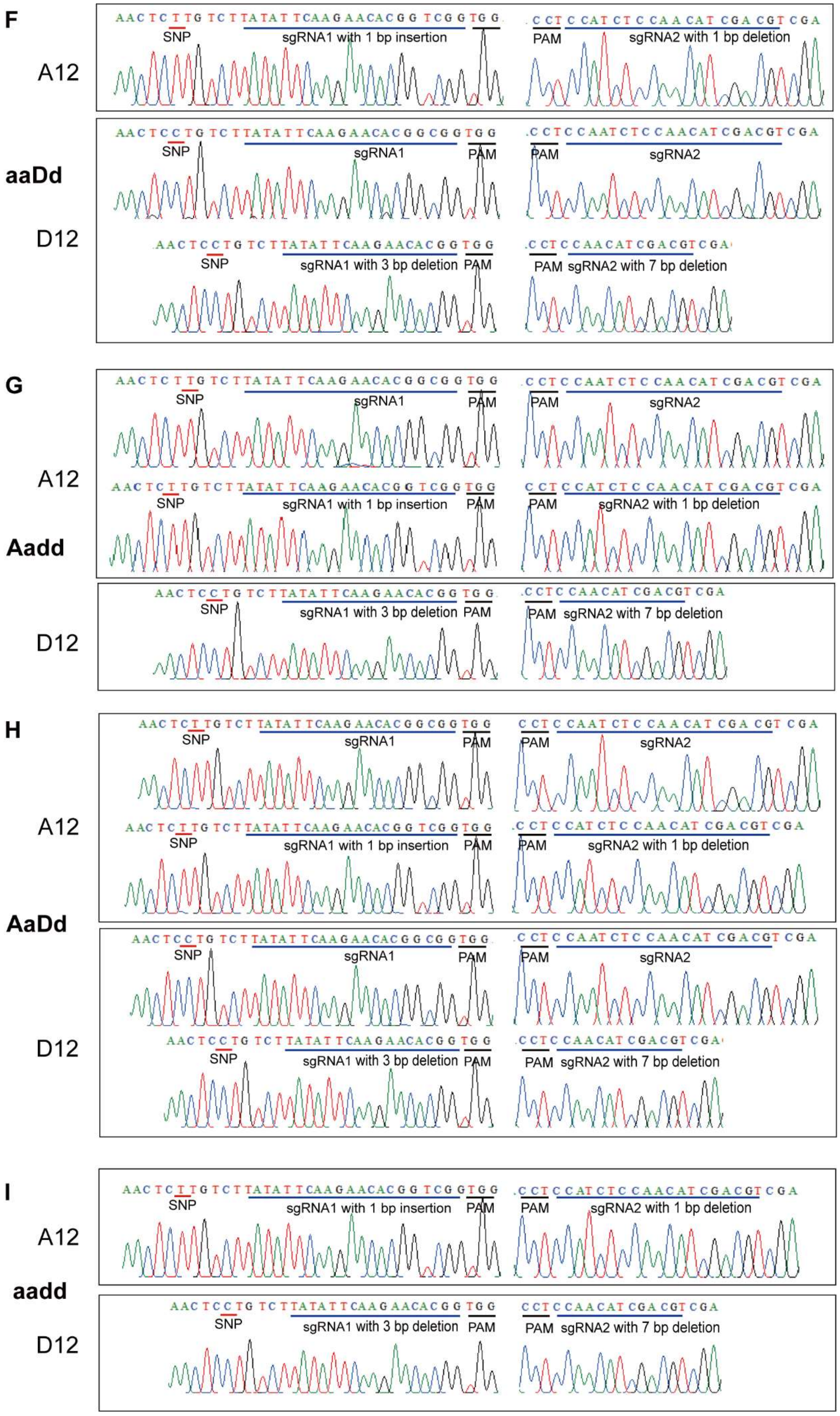
F
A12
SNP
sgRNA1 with 1 bp insertion
PAM
sgRNA2 with 1 bp deletion
aaDd
sgRNA1
sgRNA2
D12
sgRNA1 with 3 bp deletion
sgRNA2 with 7 bp deletion
G
A12
Aadd
D12
H
A12
AaDd
D12
I
A12
aadd
D12

Supplemental Figure 11. Sequencing peak traces of sgRNA target sites in (#*mml3s-8-1* × J668)F_2.

(A–I) Wild-type *GhMML3_A12* and *GhMML3_D12* are denoted as “A” and “D”, and the corresponding edited versions as “a” and “d”. Red lines indicate the SNPs used to distinguish chromosomes A12 and D12. PAM regions and sgRNAs sites are underlined with black and blue lines, respectively.

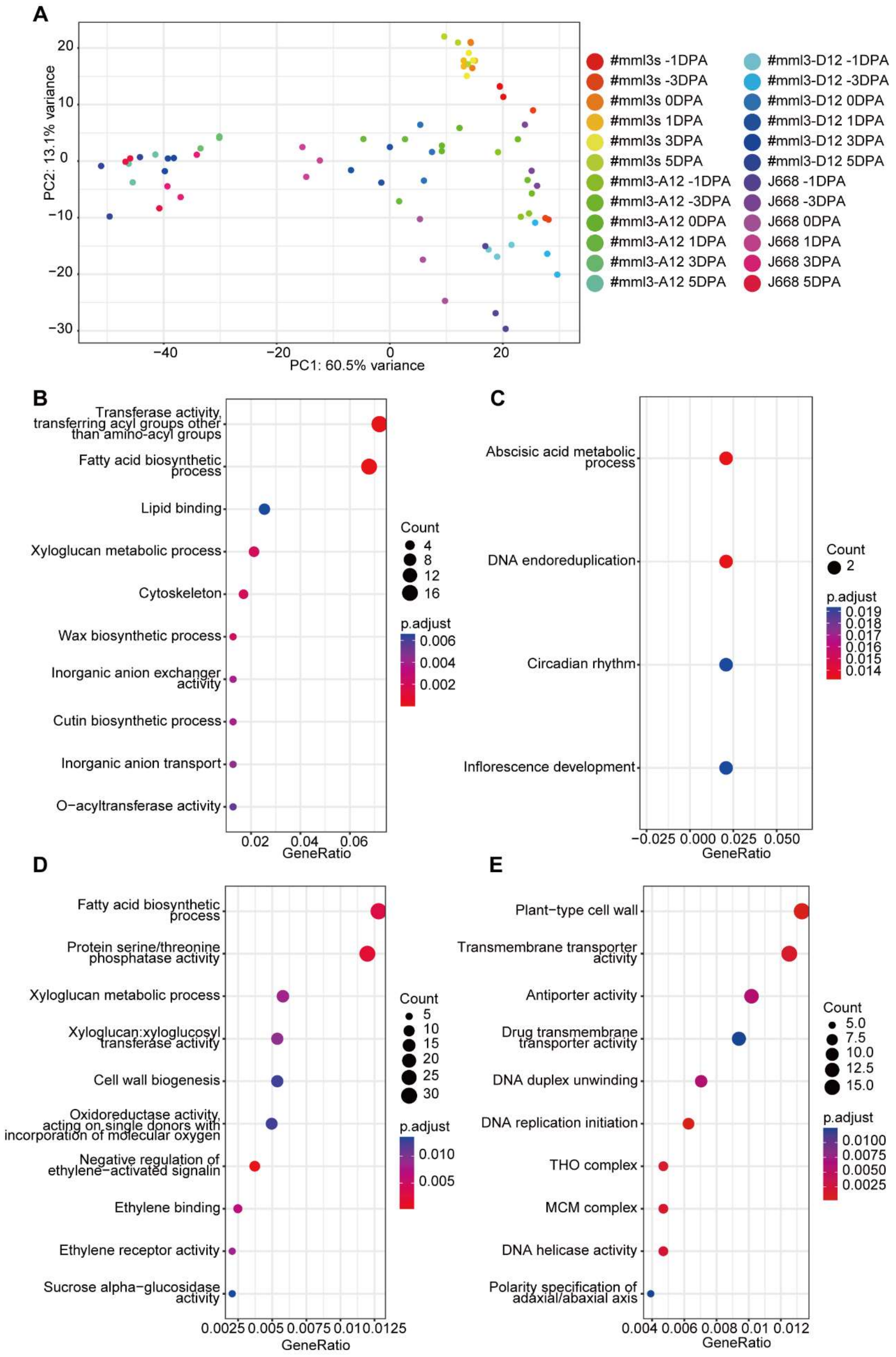

Supplemental Figure 12. RNA-seq analysis of *GhMML3* gene-edited materials. **(A)** PCA analysis of RNA data from J668, *#mml3s*, *#mml3-A12*, and *#mml3-D12* plants. **(B–C)** Gene Ontology (GO) enrichment analysis of down- and up-regulated

DEGs in fuzzless-linted mutants (*#mml3-A12*/*#mml3-D12*) vs J668. **(D–E)** GO enrichment analysis of down- and up-regulated DEGs in *#mml3s* vs J668.

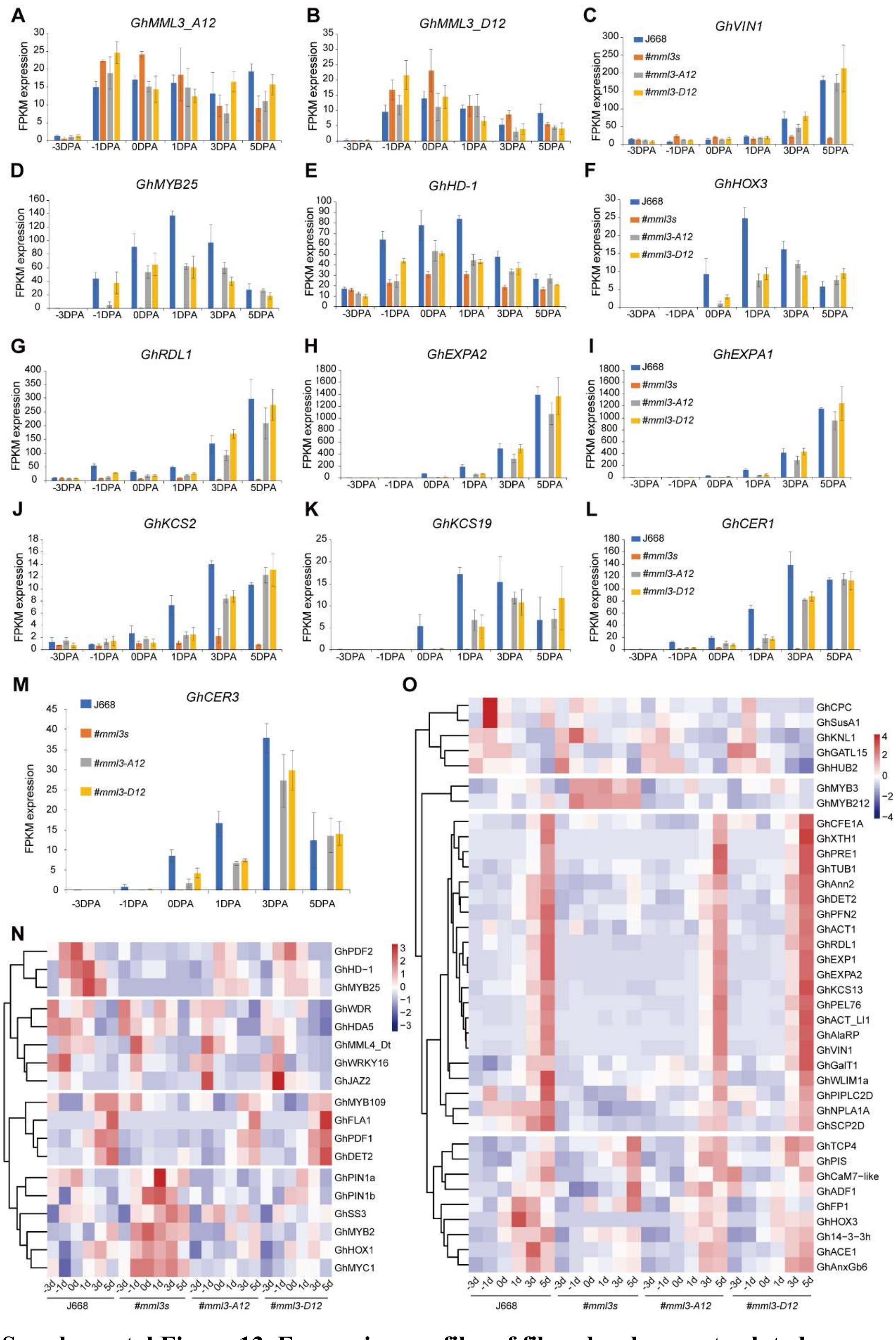

Supplemental Figure 13. Expression profiles of fiber development-related genes in *GhMML3* gene-edited materials.

(A–M) Expression profiles of important fiber development-related genes in J668, *#mml3s*, *#mml3-A12*, and *#mml3-D12*. Expression is presented as FPKM values.

Error bars represent SD of three biological replicates. **(N)** RNA-seq expression heatmap of known fiber initiation-related genes in J668, *#mml3s*, *#mml3-A12*, and *#mml3-D12*. **(O)** RNA-seq expression heatmap of known fiber elongation-related genes in J668, *#mml3s*, *#mml3-A12*, and *#mml3-D12*.

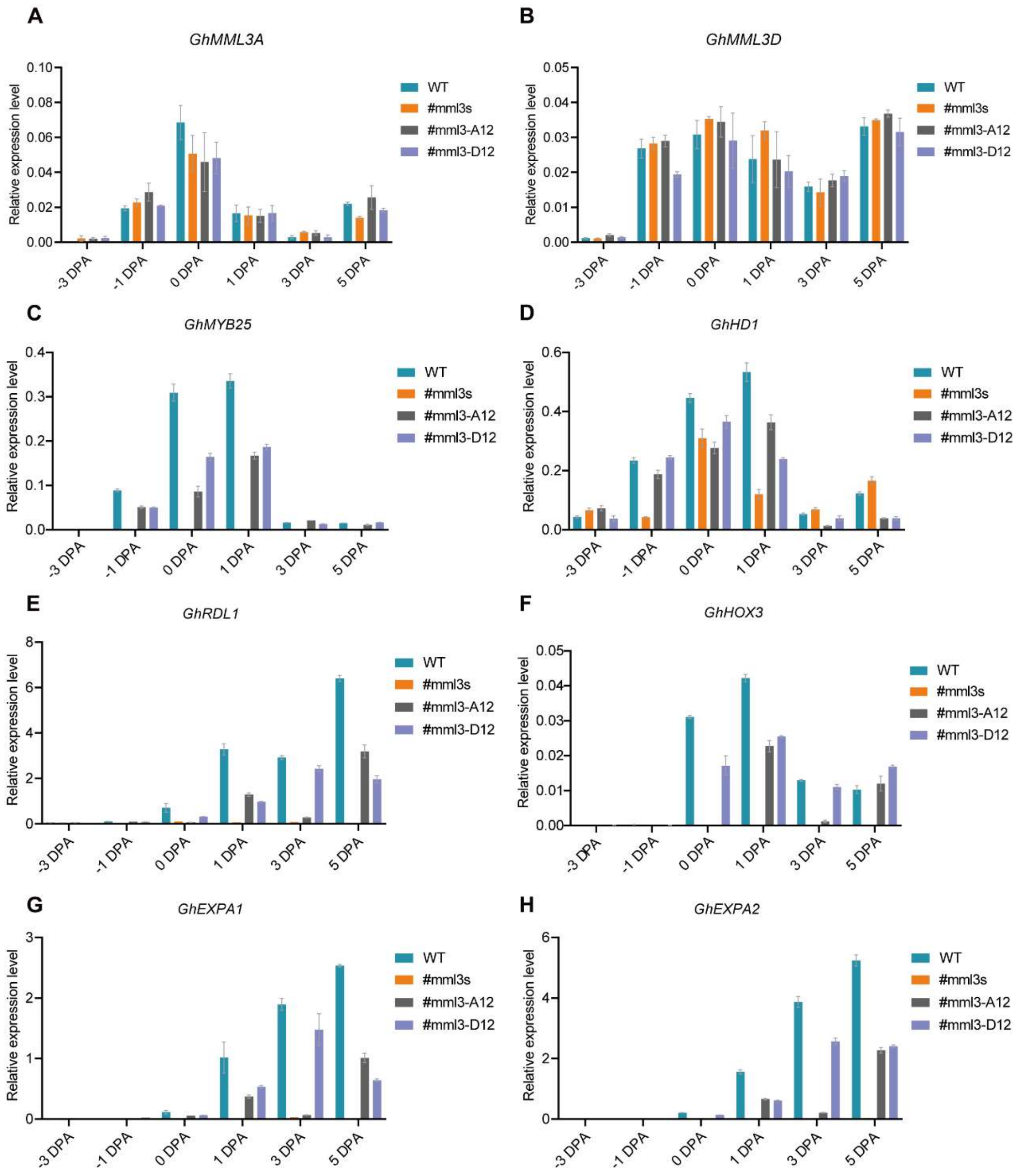

Supplemental Figure 14. Quantitative reverse transcription PCR determination of fiber development-related genes expression in the ovules of gene editing lines. The data are presented as the mean ± SEM of three biological replicates.

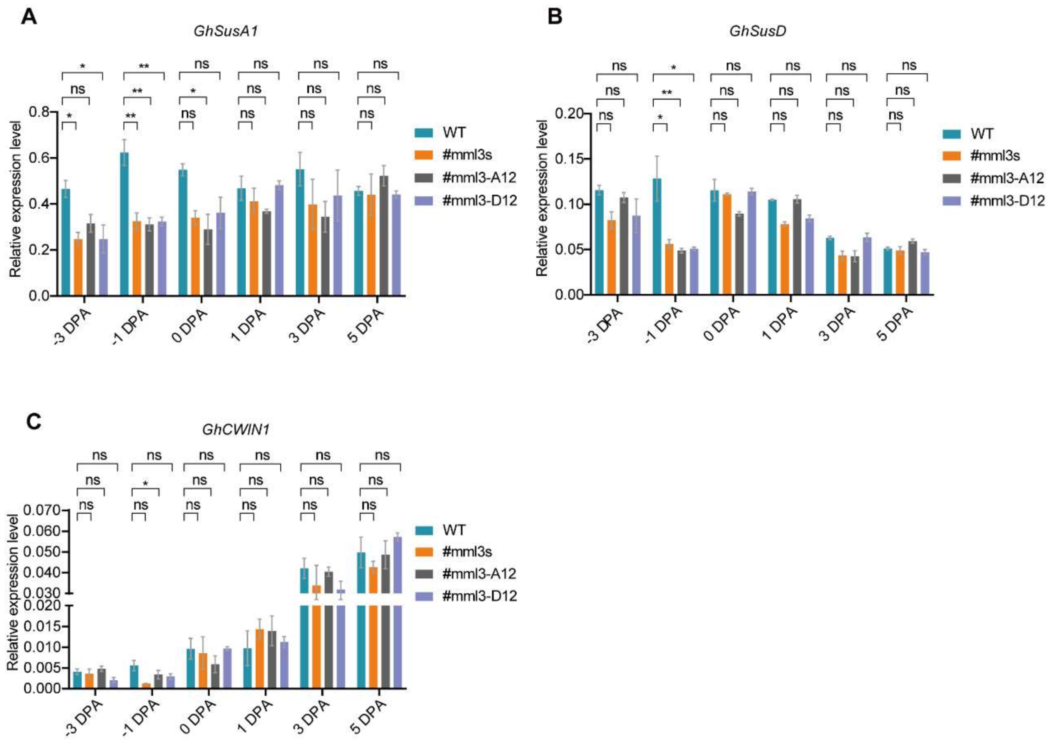

Supplemental Figure 15. Quantitative reverse transcription PCR determination of *GhSusA1*, *GhSusD* and *GhCWIN1* expression in the ovules of gene editing lines.
(A-C) Statistical significance was determined using one-way ANOVA. Data are represented as means ± SEM of three biological replicates.

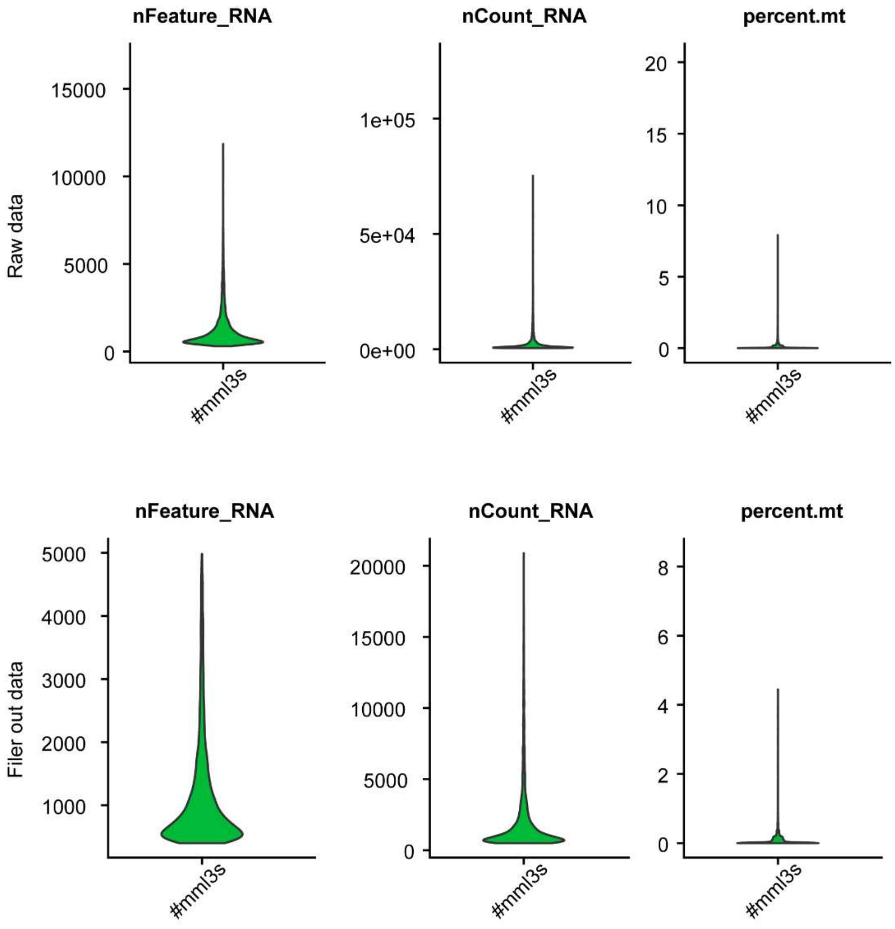

Supplemental Figure 16. Data processing for single-cell.

Raw sequencing data and filter out data of scRNA data of *#mml3s*.

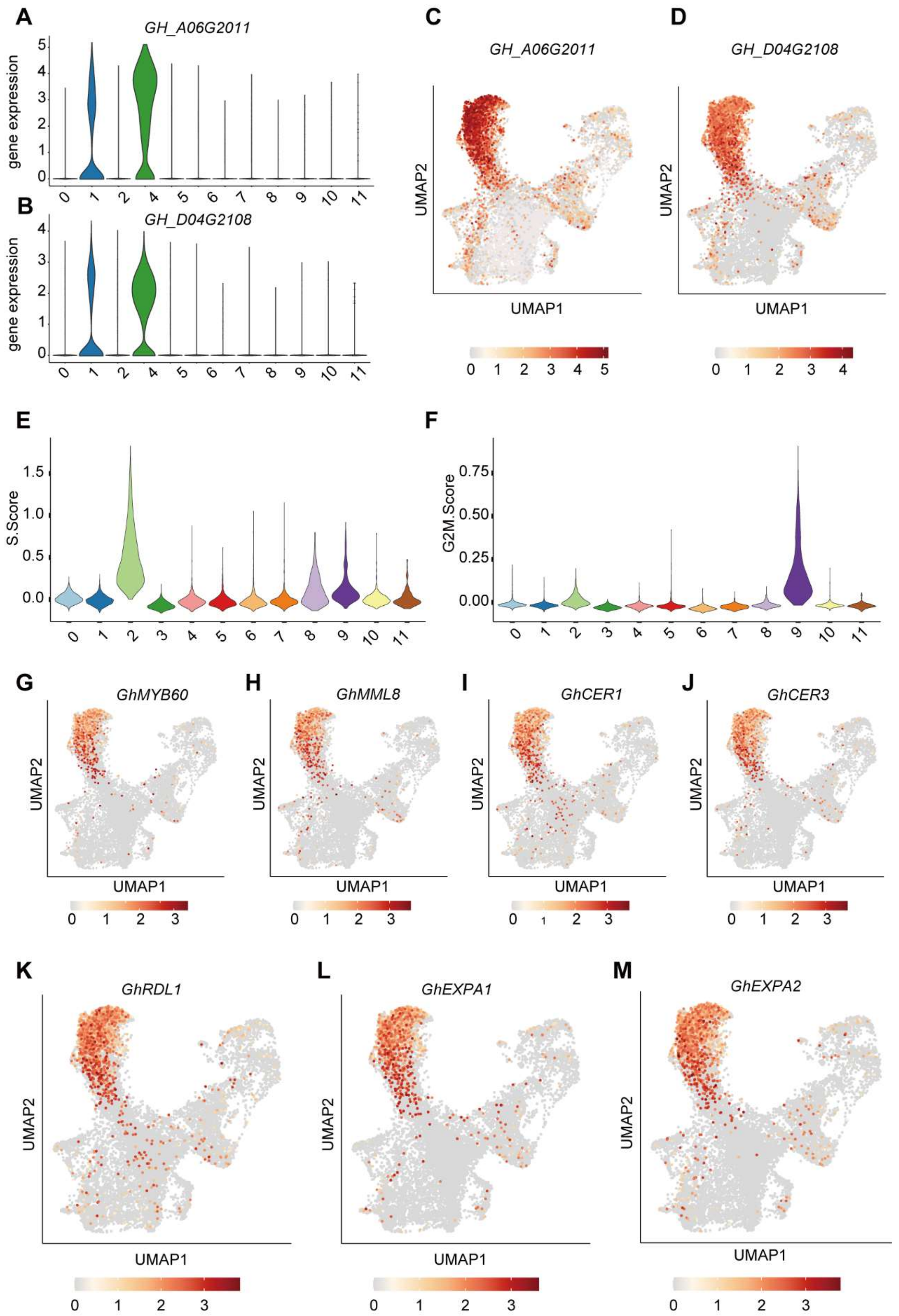

Supplemental Figure 17. Annotation of fiber cells and proliferating cell types. **(A–B)** Violin plots depicting the expression of *GhMYB25* (*GH_D04G2108*) and *GhPPO7* (*GH_A06G2011*). **(C–D)** UMAP plots representing the expression patterns of *GhMYB25* (*GH_D04G2108*) and *GhPPO7* (*GH_A06G2011*). **(E–F)** Plot of cell

cycle fractions suggesting that Cluster 2 and Cluster 9 are respectively associated with the S and G2M phases. **(G–J)** Expression profiles of *GhMML3_D12* in different clusters of *#mml3s* and WT. **(K–N)** Respective UMAP plots representing the expression patterns of *GhMYB25*, *GhHD-1*, *GhVIN1*, and *GhHOX3*.

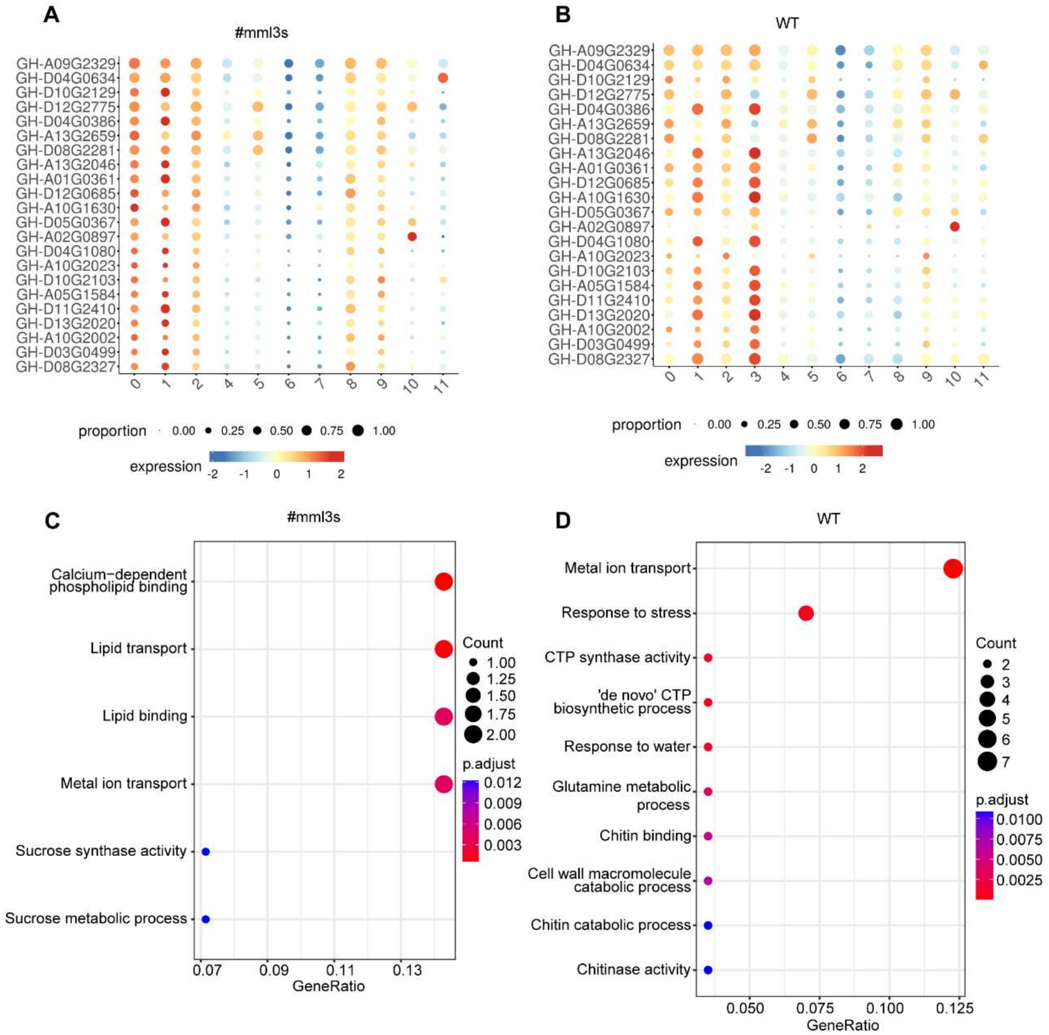

Supplemental Figure 18. Comparative expression of highly-expressed *#mml3s* Cluster 0 genes in *#mml3s* and WT samples.

(A) Expression of highly-expressed *#mml3s* Cluster 0 genes in *#mml3s* clusters. **(B)** Expression of the same genes in WT clusters. **(C)** GO enrichment of highly-expressed *#mml3s* Cluster 0 genes. **(D)** GO enrichment of highly-expressed WT Cluster 0 genes.

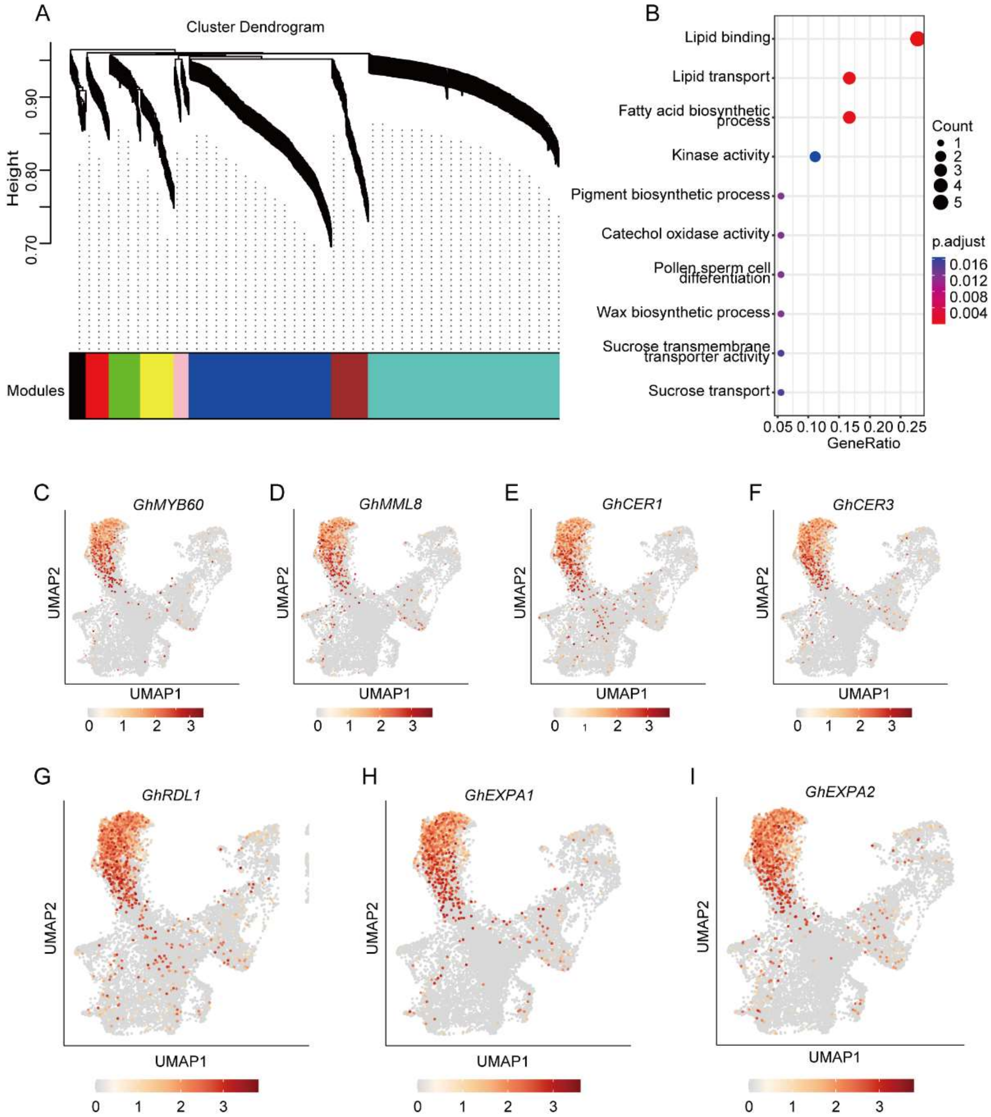

Supplemental Figure 19. Hierarchical cluster tree by WGCNA and expression profiles of fiber-related genes in #*mml3s* and WT samples.

(A) Hierarchical cluster tree showing co-expression modules identified by WGCNA. **(B)** GO enrichment analysis of genes in black module of WGCNA. **(C–F)** UMAP expression patterns of the potential fiber development-related genes *GhMYB60*, *GhMML8*, *GhCER1*, and *GhCER3* in WT and #*mml3s*. **(G–J)** UMAP plots for the fiber elongation-related genes *GhRDL1*, *GhEXPA1*, and *GhEXPA2* in WT and #*mml3s.*

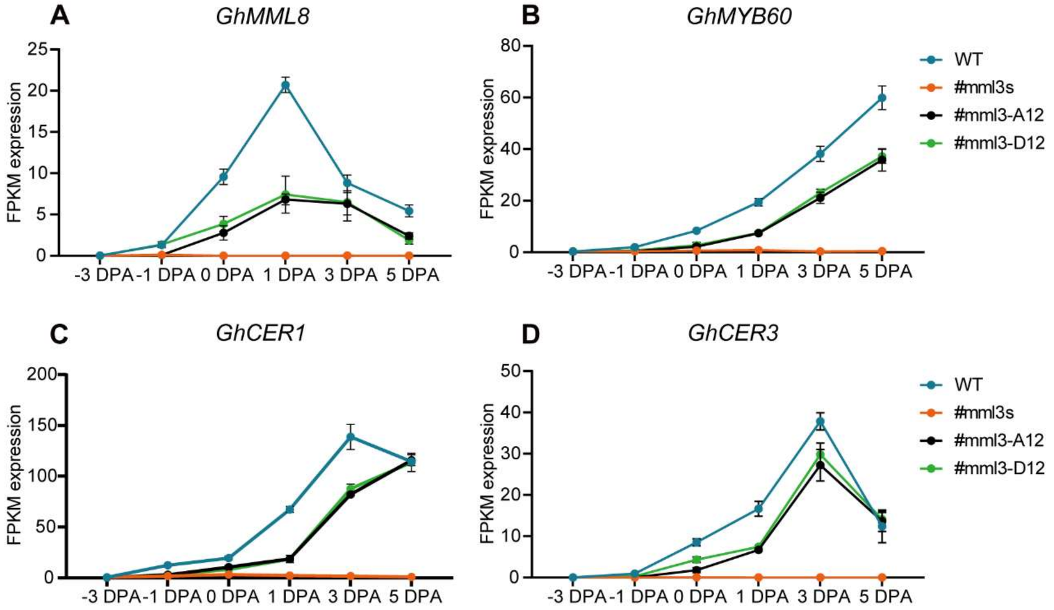

Supplemental Figure 20. Expression trends of potential fiber-related genes during fiber initiation and development.

Data are represented as means ± SEM of three biological replicates.

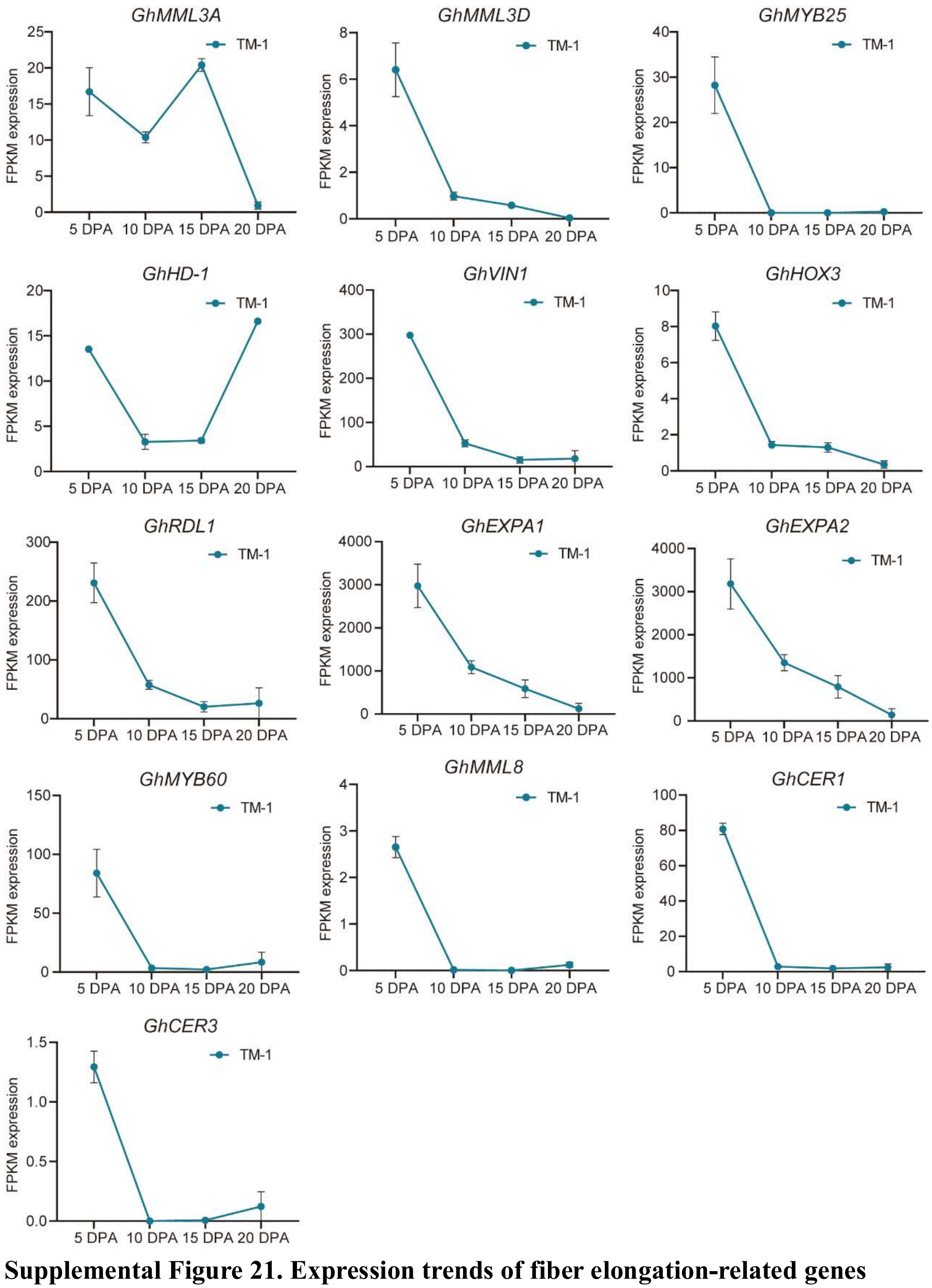

Supplemental Figure 21. Expression trends of fiber elongation-related genes during the rapid elongation period (5~20 DPA).

Data are represented as means ± SEM of three biological replicates.
